# Extended O‐Doped Polycyclic Aromatic Hydrocarbons

**DOI:** 10.1002/anie.201509517

**Published:** 2016-04-08

**Authors:** Daphné Stassen, Nicola Demitri, Davide Bonifazi

**Affiliations:** ^1^ Namur Research College (NARC)Department of ChemistryUniversity of Namur (UNamur)Rue de Bruxelles 61Namur5000Belgium; ^2^ Department of Pharmaceutical and Chemical Sciences and INSTM UdR TriesteUniversity of TriestePiazzale Europa 1Trieste34127Italy; ^3^ Elettra—Sincrotrone TriesteS.S. 14 Km163.5 in Area Science Park34149Basovizza, TriesteItaly; ^4^ School of ChemistryCardiff UniversityMain BuildingPark PlaceCardiffCF10 3ATUK

**Keywords:** copper, cyclization, macromolecules, nanostructures, supramolecular chemistry

## Abstract

The synthesis of O‐doped benzorylenes, in which peripheral carbon atoms have been replaced by oxygen atoms, has been achieved for the first time. This includes key high‐yielding ring‐closure steps which, through intramolecular C−O bond formation, allow stepwise planarization of oligonaphthalenes. Single‐crystal X‐ray diffraction showed that the tetraoxa derivative forms remarkable face‐to‐face π–π stacks in the solid state, a favorable solid‐state arrangement for organic electronics.

Discrete and extended polycyclic all‐carbon aromatic hydrocarbons (PAHs)^1^, ^2^ have polarized great interest^3^, ^4^, ^5^, ^6^, ^7^ as ultralight materials for engineering flexible optoelectronic devices. Replacing the carbon atoms with other isostructural atoms at given positions^8^, ^9^ is now developing as a versatile functionalization strategy to control the chemical, charge‐carrier, and self‐assembly behaviors of PAHs.^10^ Specifically, in the last years, we took note of the renaissance of O‐doped aromatics such as peri‐xanthenoxanthene (PXX; Scheme 1). These molecules are in fact characterized by excellent carrier‐transport and injection properties, as well as easy processability, chemical inertness, and high‐thermal stability.^11^ Because of these properties, PXX has proven exceptional performance when used as an active organic semiconductor (OSC) in transistors for rollable OLEDs.^12^, ^13^, ^14^ However, the expansion of PXX into larger O‐doped frameworks has so far remained unexplored (Figure 1), although understanding and controlling the O‐doping ratio could provide the conceptual basis to engineer a new family of OSCs with tunable optoelectronic properties.

**Figure 1:**
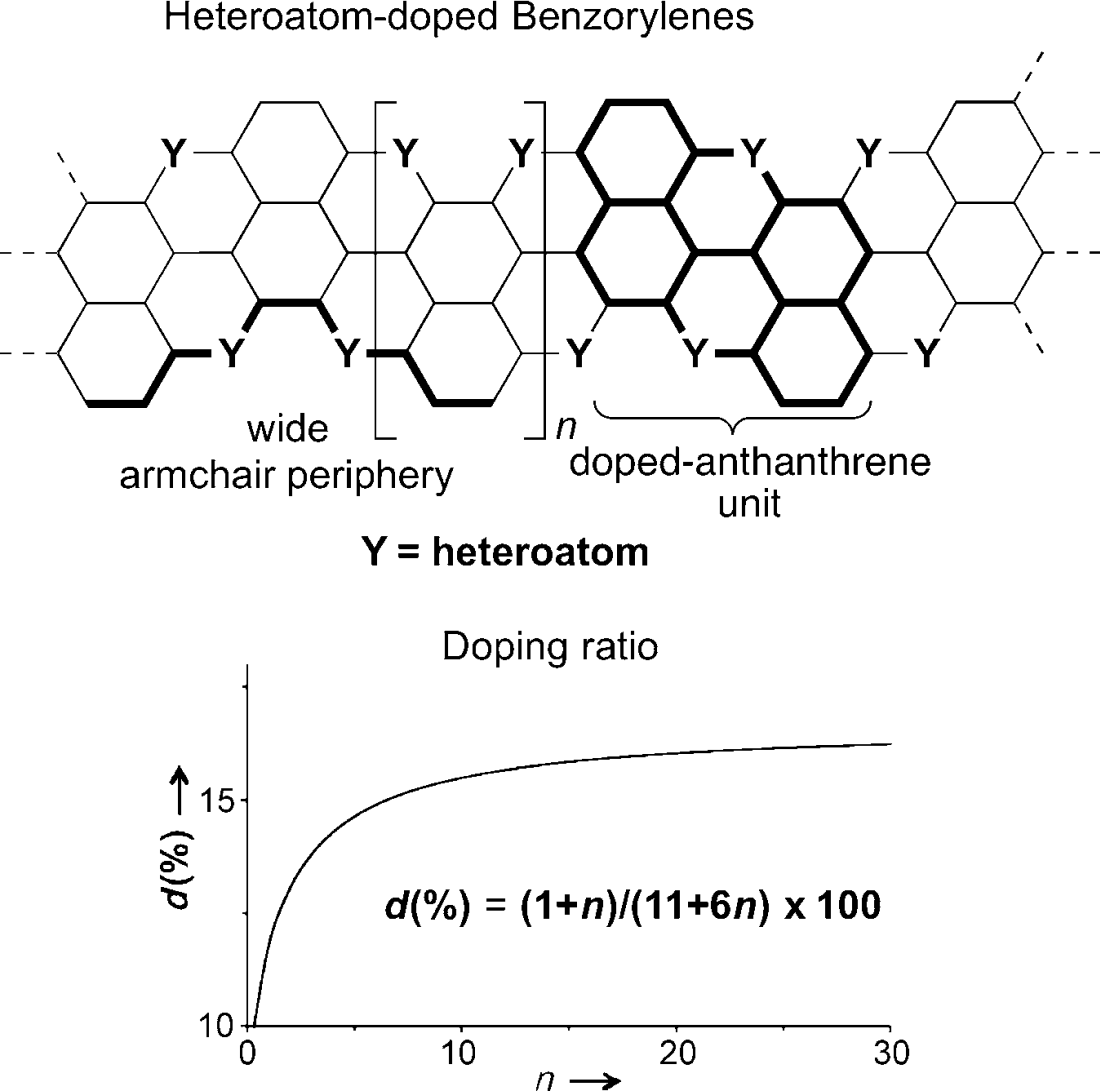
Heteroatom‐doped benzorylenes and its distinctive doping ratio (*d*) as function of the number (*n*) of dihydroxynaphthalenyl units.

**Scheme 1:**
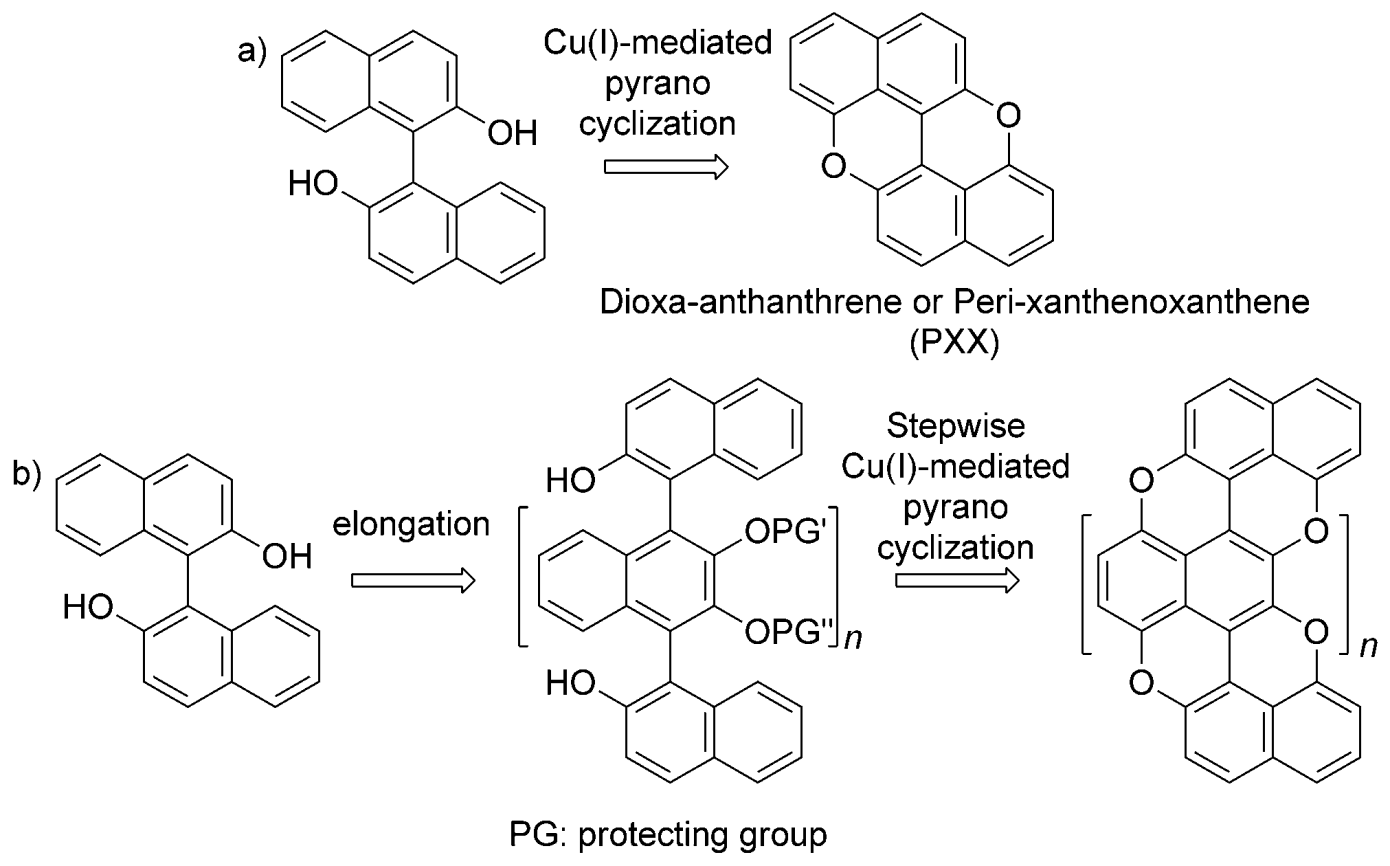
a) Pyranopyran‐fusing approach for preparing a PXX core. b) Synthetic strategy toward the O‐doped benzorylenes.

Herein we describe the synthesis of unprecedented O‐doped benzoryles (Scheme 1), like pentaphenopentaphene and napthotetraphenopyranthrene (*n=*1 and 2, respectively), featuring a tailored topological periphery and doping ratio, *d*, with the latter being controlled by the number of the monomeric units. Generally, controlled doping patterns in discrete graphene substructures are obtained through bottom‐up synthesis involving monomeric aromatic heterocyles which are preorganized in a covalent scaffold and successively planarized through oxidative C−C bond formation.^10^, ^15^, ^16^, ^17^ In our approach, we instead considered the O‐doped benzoryles derived from oligonaphthalenes with 2,3‐dihydroxynaphthalene and 2‐hydroxynaphthalene moieties as the key monomeric and capping units, respectively (Scheme 1 b). At the synthetic planning level, this consideration guided us to contemplate the oxidative metal‐mediated formation of C−O bonds in a pyranopyran motif (Scheme 1 a) as the planarization reaction. As we anticipated potential susceptibility of the 2,3‐dihydroxynaphthalenyl moieties under oxidative conditions, a decision was made to protect the hydroxy groups and to follow a two‐step planarization protocol (Scheme 1 b).

Specifically, two classes of molecules were prepared (Scheme 2): one bearing only 3,5‐di(*tert*‐butyl)phenyl substituents (**13**, **17^H^**, and **21^H^**) and another with extra 4‐*tert*‐butylphenyl side groups (**17** ^***t*****BuPh**^ and **21** ^***t*****BuPh**^) to favor solubility. The key 1,4‐linked oligonaphthalene skeletons (**12**, **14**, and **18**) were synthesized by sequential oxidative coupling reactions in the presence of racemic phenylethylamine and CuCl_2_.^18^ It should be noted that the intermediates **9**–**12** were prepared and used as racemates. The same applies for the molecules **14**–**16** and **18**–**20**.

**Scheme 2:**
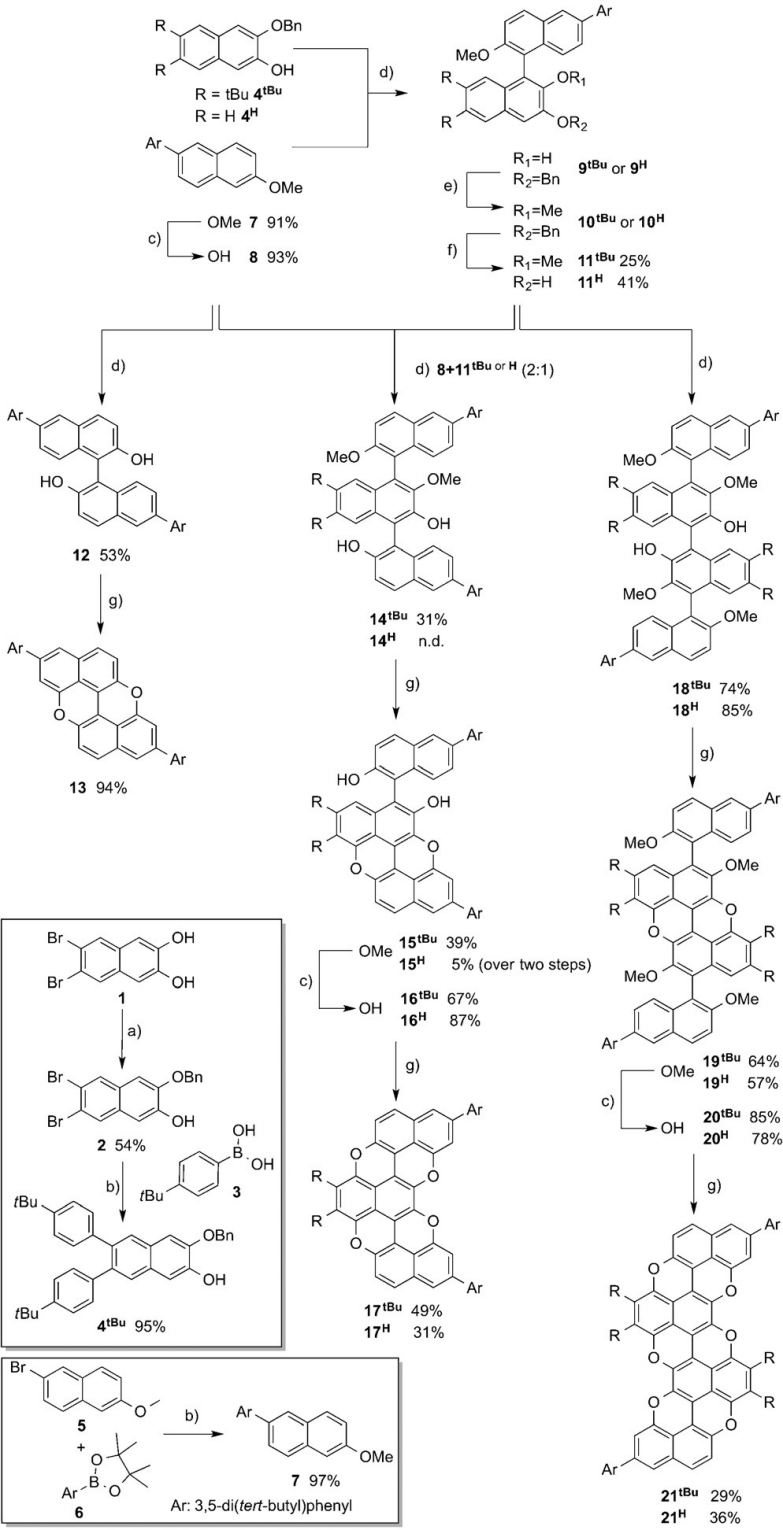
Synthetic path for **13**, **17^X^**, and **21^X^** (X=H or *t*BuPh). In the frames: synthesis of the naphthol building blocks. a) NaHCO_3_, BnBr, DMF, 100 °C; b) Cs_2_CO_3_, [Pd(PPh_3_)_4_], toluene/DMF/EtOH, microwave, 100 °C; c) BBr_3_, CH_2_Cl_2_, 0 °C; d) CuCl_2_, (±)‐1‐phenylethylamine, MeOH, CH_2_Cl_2_, 0 °C; e) K_2_CO_3_, MeI, acetone, reflux; f) H_2_, Pd/C, CHCl_3_, AcOH; g) CuI, PivOH, DMSO, 130–145 °C.

To commence, the naphthol **8** (prepared starting from 6‐bromo methoxynaphthalene **5** by a Suzuki cross‐coupling reaction followed by cleavage of the methoxy group with BBr_3_) was dimerized by copper(II)‐mediated oxidative coupling into binaphthyl **12**, which was used as model substrate. Despite numerous works describing the preparation of dibenzofurans,^19^ only a few synthetic strategies have been developed to date for the formation of benzopyranes.^20^ Amongst those, the modified protocol described by Pummerer and co‐workers^21^ with CuO allowed the transformation of **12** into the PXX derivative **13** in 42 % yield. However, when CuI was used in the presence of O_2_ and PivOH in DMSO at 140 °C,^22^ we exceptionally improved the yield to 94 %. In any circumstances, CuOAc gave inferior yields while other transition metals gave either low yields or no conversion.

For preparing the quarternaphthalene derivatives, **8** was cross‐coupled with either the monobenzyl dihydroxynaphthalene **4^H^** or **4** ^***t*****BuPh**^ (the latter prepared from the 6,7‐dibromo precursor, **1**,^23^ through double Suzuki cross‐coupling and monobenzylation reactions) by copper(II)‐promoted cross‐coupling to yield the monohydroxy binaphthalenes **11^H^** and **11** ^***t*****BuPh**^, respectively, after methylation and cleavage of the benzyl ether. Subsequent oxidative dimerization of **11^H^** and **11^tBu^** gave the quarternaphthalenes **18^H^** (85 %) and **18** ^***t*****BuPh**^ (74 %), respectively, as isomeric mixtures. Intramolecular etherification of **18^H^** and **18** ^***t*****BuPh**^ afforded the intermediates **19^H^** and **19** ^***t*****BuPh**^, respectively, as diastereoisomeric mixtures where the *cis*‐ (*cis*‐**19^H^** and *cis*‐**19** ^***t*****BuPh**^) and *trans* (*trans*‐**19^H^** and *trans*‐**19** ^***t*****BuPh**^) isomers could be easily separated. Small transparent crystals of both isomers of **19** ^***t*****BuPh**^ were obtained by vapor diffusion. X‐ray analysis confirmed the presence of the central pyranopyran cycle with two naphthalenyl substituents in the *cis* and *trans* configurations (Figure 2). Removal of the methyl protecting groups by BBr_3_ and subsequent the CuI‐mediated ring‐closure reaction led to the formation of the tripyranopyran derivatives **21^H^** and **21** ^***t*****BuPh**^ in 36 % and 29 % yield, respectively. The fully conjugated tetramers **21^H^** and **21** ^***t*****BuPh**^ were unambiguously identified by HR‐MALDI through the detection of the peaks corresponding to the molecular ions (*M* ^+^) at *m*/*z* 966.3935 (C_68_H_54_O_6_^+^, calc.: 966.3920) and 1494.7526 (C_108_H_102_O_6_^+^, calc.: 1494.7556), respectively.

**Figure 2:**
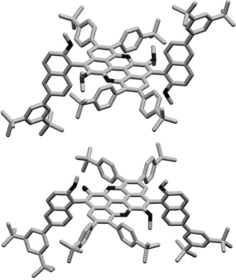
Crystal structures of *trans*‐**19** ^***t*****BuPh**^ (above) and *cis*‐**19** ^***t*****BuPh**^ (below).^25^ Space groups *P*21/*c* and $P\bar{1}$ , respectively. C gray, O black.

In parallel, the fused bispyranopyran **17^H^** and **17** ^***t*****BuPh**^ were also prepared. The naphthol **8** was cross‐coupled with **11^H^** and **11** ^***t*****BuPh**^ to afford the dihydroxyternaphthalenes **14^H^** and **14** ^***t*****BuPh**^, respectively, as isomeric mixtures (dimer **12** and tetramers **18^H^** and **18** ^***t*****BuPh**^ were also obtained as side‐products). Successive pyranopyran fusion led to the corresponding intermediates **15^H^** and **15** ^***t*****BuPh**^. BBr_3_‐promoted cleavage of the methyl groups followed by the oxidative cyclization yielded **17^H^** and **17** ^***t*****BuPh**^ in 31 % and 49 % yield, respectively. Again, **17^H^** and **17** ^***t*****BuPh**^ were clearly identified by HR‐MALDI through detection of the peaks related to the molecular ions (M^+^) at *m*/*z* 812.3848 (C_58_H_52_O_4_^+^, calc.: 812.3866) and 1076.5760 (C_78_H_76_O_4_^+^, calc.: 1076.5744), respectively. Surprisingly, ^1^H NMR investigations of the final molecules were inconclusive as only broad peaks were observed after planarization.

However, upon addition of a few drops of NH_2_NH_2_ to a [D_8_]THF solution of some samples of **17** ^***t*****BuPh**^, a sharpening of the proton resonances was observed. As shown in Figure 3, the ^1^H NMR spectrum of the sample containing NH_2_NH_2_ features well‐resolved peaks, thus perfectly integrating the 22 aromatic proton resonances expected for **17** ^***t*****BuPh**^. This spectrum could suggest that a fraction of the compound is present as a radical cation. Notably, for **17^H^**, **21^H^** and **21** ^***t*****BuPh**^ the addition of NH_2_NH_2_ was fruitless, and only broad resonances in the ^1^H NMR spectra were observed. To further corroborate the chemical structure of the bis(pyranopyran) derivative, crystals suitable for X‐ray diffraction analysis were obtained by vapor diffusion of *i*PrOH into a CH_2_Br_2_ solution of **17^H^** (Figure 4). The X‐ray structure confirms the nearly flat boomerang‐like shape of the pentaphenopentaphene framework, in which four oxygen atoms have replaced four carbon atoms at the peripheries.

**Figure 3:**
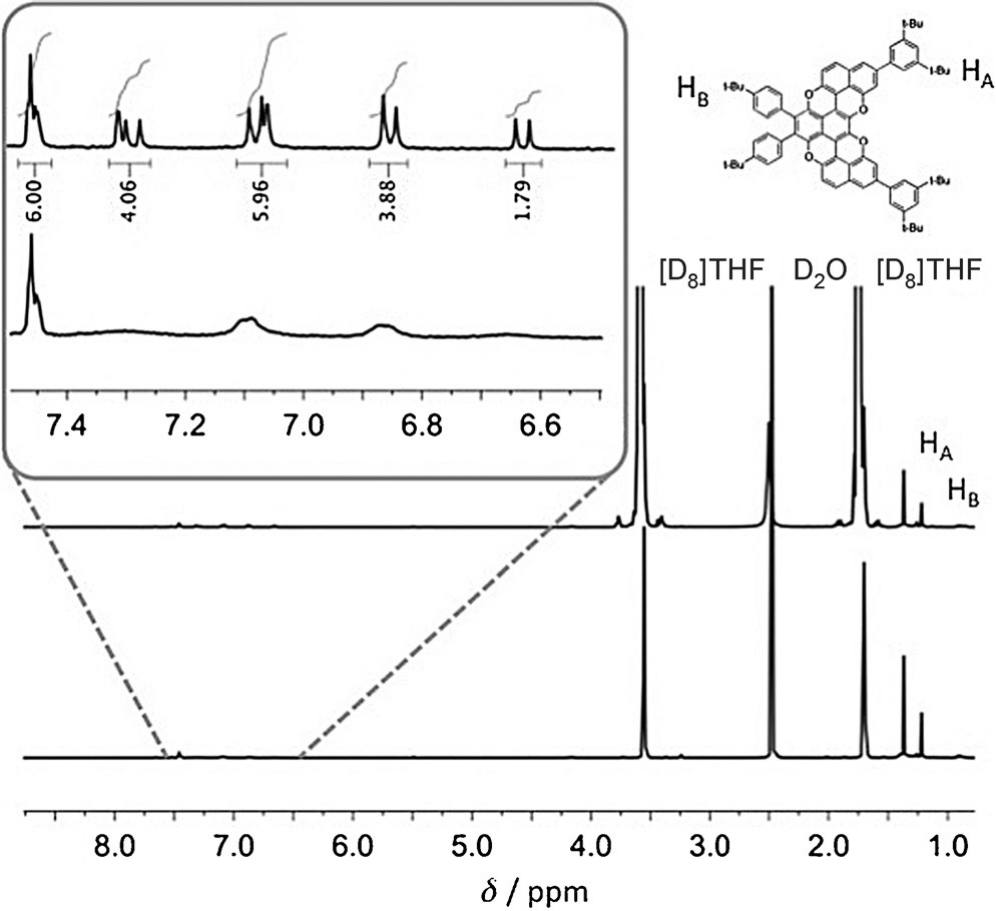
^1^H‐NMR spectra (400 MHz, [D_8_]THF) of **17** ^***t*****BuPh**^ before (below) and after addition of NH_2_NH_2_ (above).

**Figure 4:**
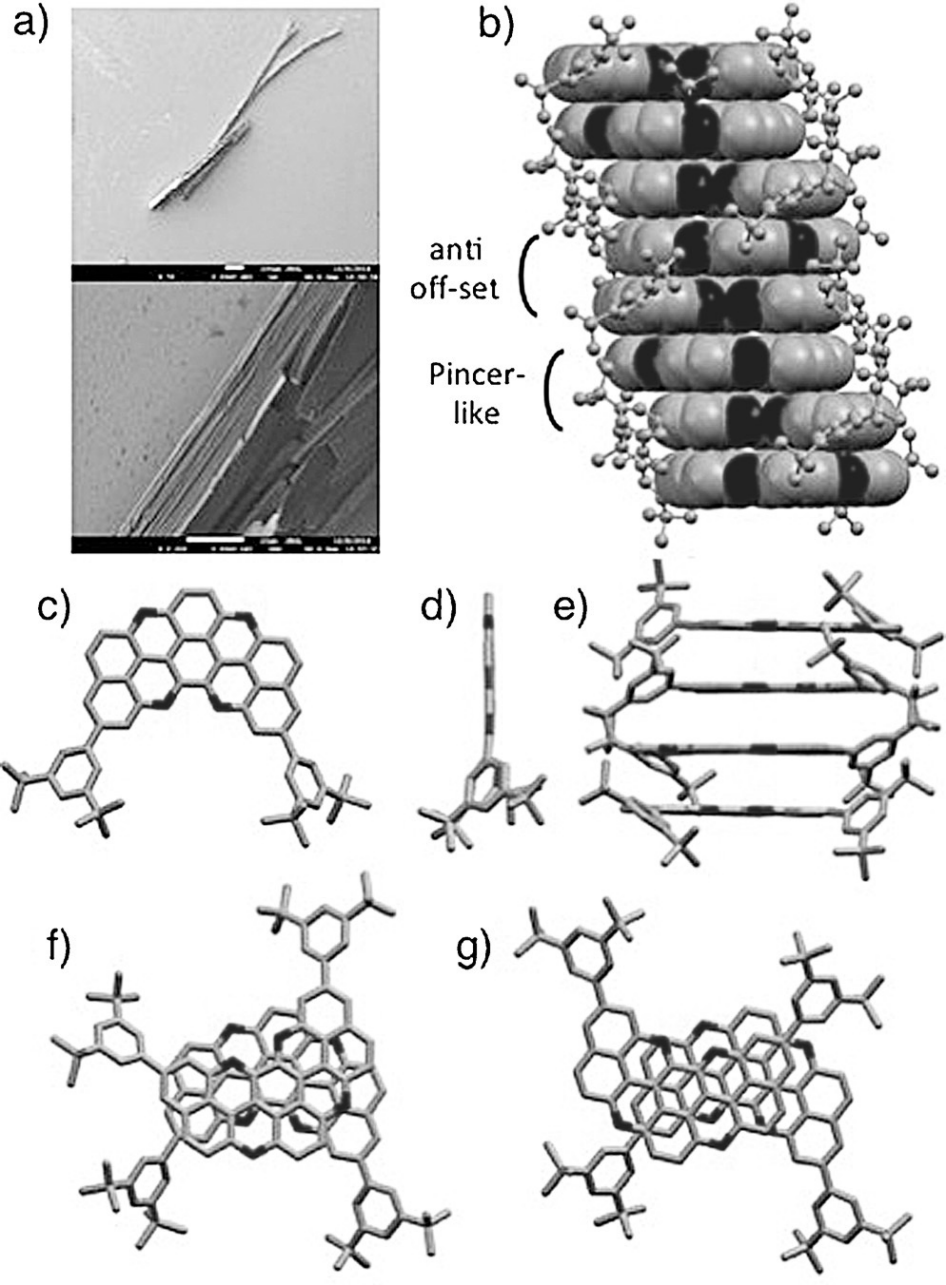
a) SEM images of the crystals of **17^H^** displaying a lamellar‐like texture. b–e) Solid‐state columnar π–π stacks with an interplanar distance of 3.3 Å. c,d) Top‐ and side‐view of the crystal structure. f) Pincerlike and g) *anti* off‐set π–π stacking arrangements. Space group: $P\bar{1}$ . C gray, O black.

Looking at its organization at the solid state (Figures 4 b and e), one can clearly evidence the presence of a columnar arrangement in which the molecules are organized in π–π stacks, with an average interplanar spacing of 3.3 Å. Two face‐to‐face stacking modes are apparent: a pincerlike stack (Figure 4 f), in which two crystallographically independent molecules are facing each other in an antiparallel fashion with a relative angle of about 36°, and an *anti* offset shift (Figure 4 g), where two molecules stack in an antiparallel arrangement with a lateral offset of about 3.4 Å and 0.8 Å for the other crystallographically independent molecule.

UV‐vis steady‐state absorption spectra of **13**, **17^X^**, **21^X^** are shown in Figure 5. While the spectrum of the PXX derivative features the typical electronic transitions at *λ*=392, 421 and 449 nm, the spectra for conjugates **17^X^** and **21^X^** appear much broader. In particular, the absorption spectra of **17^H^** and **17** ^***t*****BuPh**^ display unstructured low‐intensity red‐shifted bands at *λ*=412, 509 and 552 nm, whereas only a long absorption tail reaching *λ*=650 nm is observed for both **21^H^** and **21** ^***t*****BuPh**^. Variable‐temperature measurements did not display any significant sharpening of the electronic transitions even at elevated temperatures (80 °C; see Figure SI5 in the Supporting Information). Together with the ^1^H NMR results, the intense broadening of the electronic transitions suggests that these O‐doped molecules most likely undergo strong aggregation. This aggregation can possibly occur either by simple π‐stacking interactions between neutral molecules or between a radical‐cation with its neutral counterparts in mixed‐valence complexes,^24^ or through the formation of covalent oligomers possibly deriving from a radical recombination followed by proton elimination.

**Figure 5:**
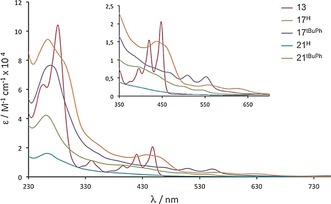
Steady‐state UV‐vis absorption spectra of **13**, **17^X^**, and **21^X^** in CH_2_Cl_2_.

While the formation of mixed‐valence species can be realistically excluded because of the absence of electronic transitions fingerprinting a charge transfer in the NIR spectral region (see Figure SI2),^24^ HRMS‐MALDI and tandem mass (MSMS) measurements unambiguously displayed the presence of oligomeric species, and are supportive of the presence of aggregates even in the gas phase. In particular, peaks at *m*/*z* 1624 and 3247 could be discerned, thus suggesting the presence of dimeric and tetrameric species for **17^H^** (Figure SI8 a). MSMS analysis at *m*/*z* 3247 and 1624 suggest that the dimers are formed by a combination of covalent [(**17^H^**)_2_−2 H] and noncovalent [(**17^H^**)_2_] complexes (Figure SI8 c,d,e,f,h). In contrast, the tetrameric species (Figure SI8 b,g,h) are constituted by noncovalent complexes of covalent dimers, [(**17^H^**)_2_−2 H]_2_.

In conclusion, we have described the first methodology to prepare unprecedented O‐doped benzorylenes by using a stepwise planarization strategy. This approach involves the simultaneous formation of C−O bonds through an intramolecular copper(I)‐mediated oxidative reaction originating pyranopyran rings. First X‐ray diffraction showed that the tetraoxa derivative undergoes strong π‐stacking in the solid state to form lamellar‐like microstructures. The remarkable propensity of this class of molecules to undergo self‐aggregation is intriguing in view of the design of organic materials to be used in optoelectronic devices. Detailed electron paramagnetic resonance, electrochemical, and conductivity studies are under investigation to fully understand the chemical behavior of this class of O‐doped π‐conjugated framework, as well as their potential for engineering transistors.

## Supporting information

As a service to our authors and readers, this journal provides supporting information supplied by the authors. Such materials are peer reviewed and may be re‐organized for online delivery, but are not copy‐edited or typeset. Technical support issues arising from supporting information (other than missing files) should be addressed to the authors.

Supplementary
