# Supplementary material for: Extended O‐Doped Polycyclic Aromatic Hydrocarbons
Source: Angew Chem Int Ed Engl. 2016 Apr 8;55(20):5947–51. doi: 10.1002/anie.201509517 (PMC5071652; doi:10.1002/anie.201509517)
Supplement: Supplementary file 1 — Supplementary [file ANIE-55-5947-s001.pdf]

Angewandte Chemie
Eine Zeitschrift der Gesellschaft Deutscher Chemiker

Supporting Information

Extended O-Doped Polycyclic Aromatic Hydrocarbons

*Daphné Stassen, Nicola Demitri, and Davide Bonifazi**

anie_201509517_sm_miscellaneous_information.pdf

Materials, methods and characterizations

1.1. Intrumentation 1

1.2. Materials and methods 3

1.3. Emission and absorption analysis 4

1.4. Mass spectrometry analysis 6

1.5. X-Ray Analysis 9

1.6. Synthesis. 12

1.7. NMR Analysis 38

References 61

Materials, methods and characterizations

1.1. Intrumentation

Thin layer chromatography (TLC) was conducted on pre-coated aluminum sheets with 0.20 mm Machevery-Nagel Alugram SIL G/UV254 with fluorescent indicator UV254. Column chromatography was carried out using Merck Gerduran silica gel 60 (particle size 63-200 μm). **Microwave reactions** were performed on a Biotage AB Initiator microwave instrument producing controlled irradiation at 2.450 GHz. **Melting points** (M.p.) were measured on a Büchi Melting Point B-545 in open capillary tubes and have not been corrected. **Nuclear magnetic resonance** (NMR) ^{1}H, ^{13}C and ^{19}F spectra were obtained on a 400 MHz NMR (Jeol JNM EX-400) or 270 MHz (Jeol JNM EX-270) at rt otherwise stated. Chemical shifts were reported in ppm according to tetramethylsilane using the solvent residual signal as an internal reference ($CDCl_3$: δH = 7.26 ppm, δC = 77.16 ppm; DMSO-d6: δH = 2.50 ppm, δC = 39.52 ppm). Coupling constants (J) were given in Hz. Resonance multiplicity was described as s (singlet), d (doublet), t (triplet), dd (doublet of doublets), m (multiplet) and broad (broad signal). Carbon spectra were acquired with a complete decoupling for the proton. **Infrared spectra** (IR) were recorded on (i) a BIO-RAD FTS-165 apparatus between 4000 and 600 cm^{-1}. Liquid samples have been prepared as film between two sodium chloride cells (NaCl) and solid samples were spread out in potassium bromide (KBr) before being pressed as a transparency 0.2 mm tablet or (ii) on a Perkin-Elmer Spectrum II FT-IR System with

Specac Silver Gate Evolution single-reflection ATR mounted with a diamond mono-crystal. **Mass spectrometry** was generally performed by the Centre de spectrométrie de masse at (i) the Université de Mons in Belgium were they performed ESI-MS and MALDI-MS, on using the following instrumentation. ESI-MS measurements were performed on a Waters QToF2 mass spectrometer operating in positive mode. The analyte solutions were delivered to the ESI source by a Harvard Apparatus syringe pump at a flow rate of 5 μL/min. Typical ESI conditions were, capillary voltage 3.1 kV; cone voltage 20-50 V; source temperature 80 °C; desolvation temperature 120°C. Dry nitrogen was used as the ESI gas. For the recording of the single-stage ESI-MS spectra, the quadrupole (rf-only mode) was set to pass ions from 50 to 1000 Th, and all ions were transmitted into the pusher region of the time-of-flight analyzer where they were mass analyzed with 1 s integration time. MALDI-MS were recorded using a Waters QToF Premier mass spectrometer equipped with a nitrogen laser, operating at 337 nm with a maximum output of 500 mW delivered to the sample in 4 ns pulses at 20 Hz repeating rate. Time-of-flight analyses were performed in the reflectron mode at a resolution of about 10,000. The matrix solution (1 μL) was applied to a stainless steel target and air-dried. Analyte samples were dissolved in a suitable solvent to obtain 1 mg/mL solutions. 1 μL aliquots of those solutions were applied onto the target area already bearing the matrix crystals, and air-dried. For the recording of the single-stage MS spectra, the quadrupole (rf-only mode) was set to pass ions from 100 to 1000 Th, and all ions were transmitted into the pusher region of the time-of-flight analyzer where they were analyzed with 1 s integration time or (ii) The "Fédération de Recherche" ICOA/CBM (FR2708) platform of Orléans in France. High-resolution ESI mass spectra (HRMS) were performed on a Bruker maXis Q-TOF in the positive ion mode. The analytes were dissolved in a suitable solvent at a concentration of 1 mg/mL and diluted 200 times in methanol (≈ 5 ng/mL). The diluted solutions (1μL) were delivered to the ESI source by a Dionex Ultimate 3000

RSLC chain used in FIA (Flow Injection Analysis) mode at a flow rate of 200 μL/min with a mixture of CH_3CN/H_2O+0.1% of HCO_2H (65/35). ESI conditions were as follows: capillary voltage was set at 4.5 kV; dry nitrogen was used as nebulizing gas at 0.6 bars and as drying gas set at 200°C and 7.0 L/min. ESI-MS spectra were recorded at 1 Hz in the range of 50-3000 m/z. Calibration was performed with ESI-TOF Tuning mix from Agilent and corrected using lock masses at m/z 299.294457 (methyl stearate) and 1221.990638 (HP-1221). Data were processed using Bruker DataAnalysis 4.1 software. **Electron Paramagnetic resonance** (EPR) spectra were recorded using a Bruker Elexsys E500 CW spectrometer at a frequency of 9.414 GHz. The spectra were recorded on CH_2Cl_2 solution. Magnetic meaurements as a function of the field (0-5 T) and of the temperature (2-300 K) were performed using a QD MPMS SQUID Magnetometer. Raw data were corrected for diamagnetic contribution of the sample holder and the intrinsic diamagnetism of the sample estimated by Pascal's constants.

1.2. Materials and methods

Chemicals were purchased from Sigma Aldrich, Acros Organics, TCI and ABCR and were used as received. C60 was purchased from Bucky-USA (99 %). Solvents were purchased from Sigma Aldrich, while deuterated solvents from Eurisotop. Diethyl ether and THF were distilled from sodium-benzophenone-cetyl, toluene was refluxed over calcium hydride and dichloromethane (CH_2Cl_2) was refluxed over phosphorous pentoxide. Anhydrous DMF was purchased from Acros Organics. Sulfuric acid (H_2SO_4 95%) and hydrochloridic acid (HCl 32%) were purchased from Fischer Scientific. Pyridine was purchased from Acros Organics. MeOH, $CHCl_3$ and acetone were purchased as reagent-grade and used without further purification. Low temperature baths were prepared using different solvent mixtures depending on the desired temperature: -78°C with acetone/dry ice, -40 °C with CH_3CN/liquid N_2, -10 °C with ice-H_2O/NaCl, and 0 °C with ice/H_2O. Anhydrous conditions were achieved

by drying Schlenk tubes or 2-neck flasks by flaming with a heat gun under vacuum and then purging with Argon. The inert atmosphere was maintained using Argon-filled balloons equipped with a syringe and needle that was used to penetrate the silicon stoppers used to close the flasks' necks. Additions of liquid reagents were performed using dried plastic or glass syringes. 6,7-Dibromonaphthalene-2,3-diol **1**[1S] and 2-(3,5-di-*tert*-butylphenyl)-4,4,5,5-tetramethyl-1,3,2-dioxaborolane **6**[2S] were prepared according to literature procedure.

1.3. Emission and absorption analysis

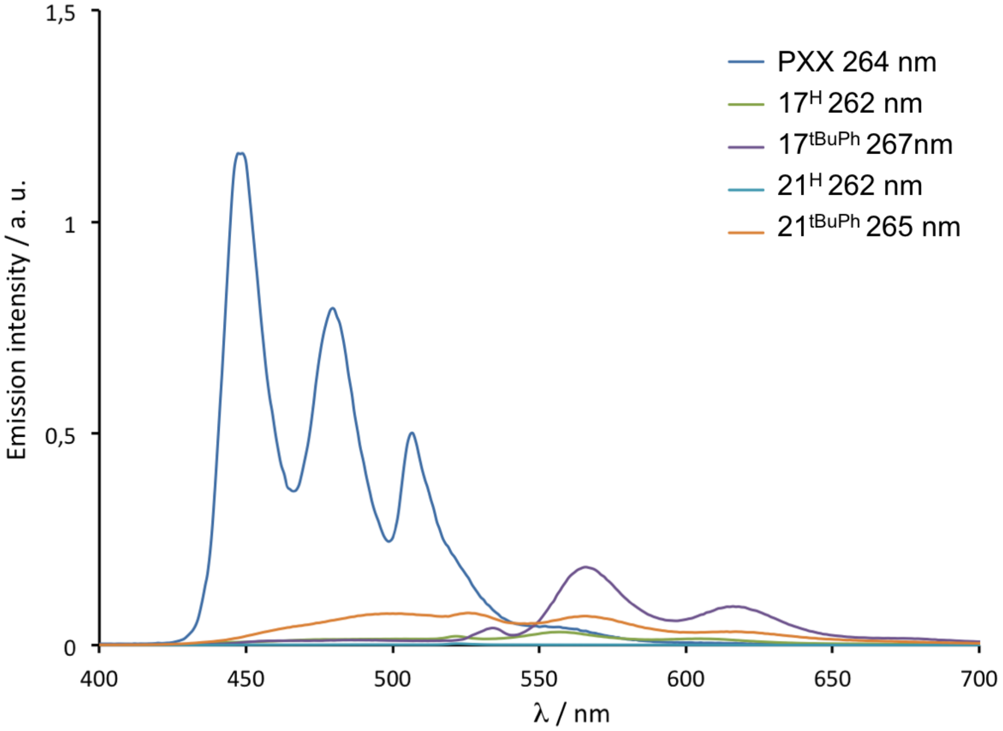

Fig. SI1. Normalised emission spectra of fused system PXX, **17**$^{\mathbf{x}}$ and **21**$^{\mathbf{x}}$ (X = H or tBuPh) recorded in CH_2Cl_2 solution at room temperature.

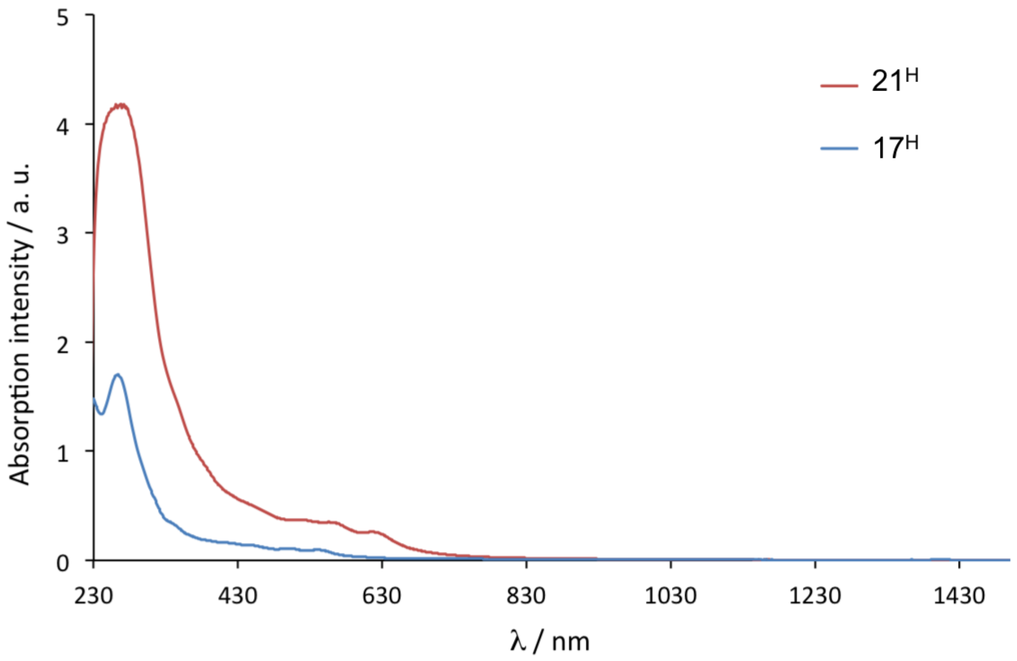

Fig. SI2. Normalised UV-vis absorption spectra of **17^H^** and **21^H^** in CH_2Cl_2 at the NIR region.

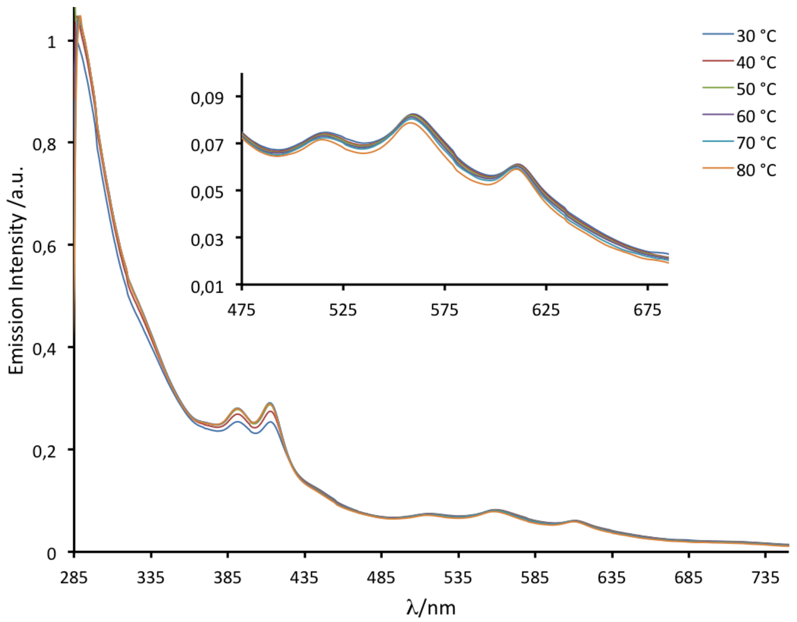

Fig. SI3. Variable temperature UV-Vis absorption spectra of **21^H^** recorded in a toluene solution (C = 0.72 10-3 M) at different temperature (from r.t. to 80 °C).

1.4. Mass spectrometry analysis

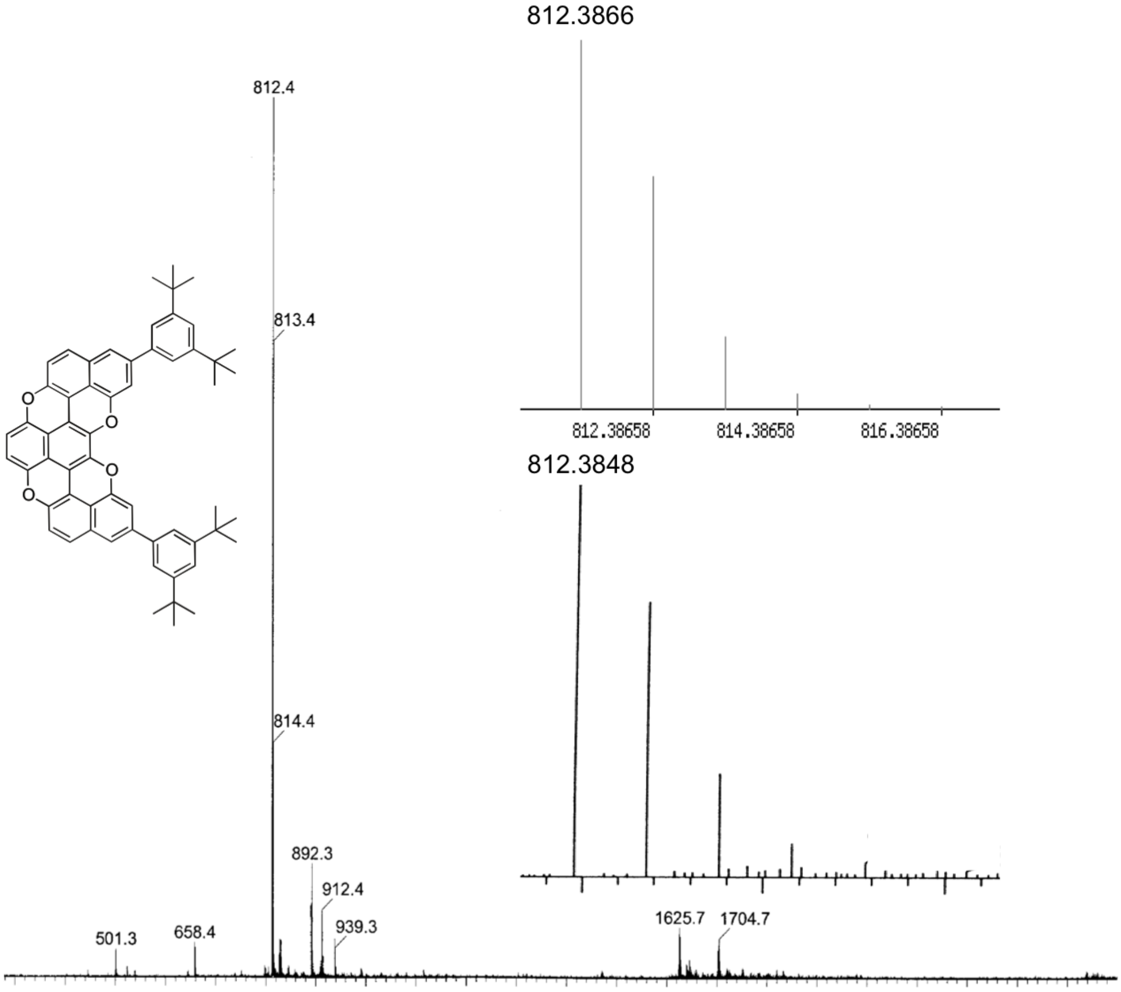

Fig. SI4. HRMS-MALDI mass spectrum of **17^{H}** in the positive ion mode (matrix: DTCB, N2-laser: 337 nm).

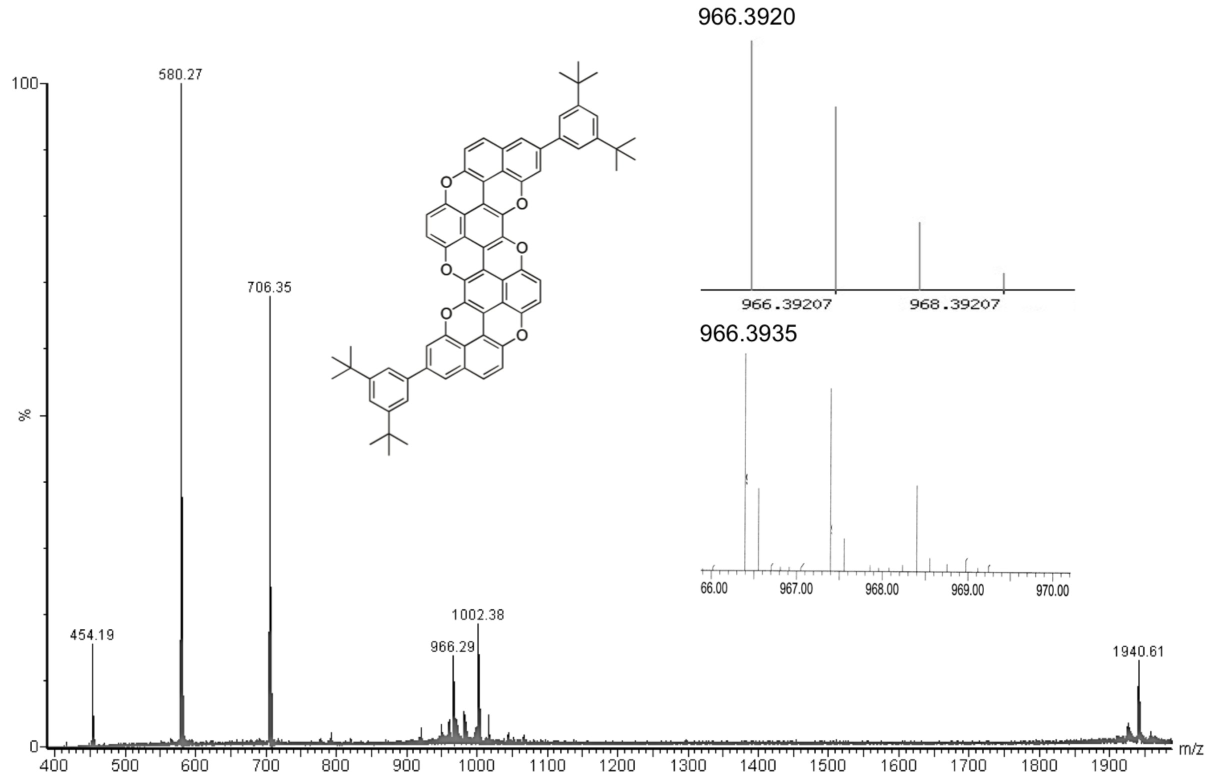

Fig. SI5. Low-resolution and HRMS-MALDI mass spectra (in the inset) of **21**^{H} in the positive ion mode (matrix: DTCB, N2-laser: 337 nm). The peak at 1002 correspond to an HCl adduct while at 1940 a dimeric specie.

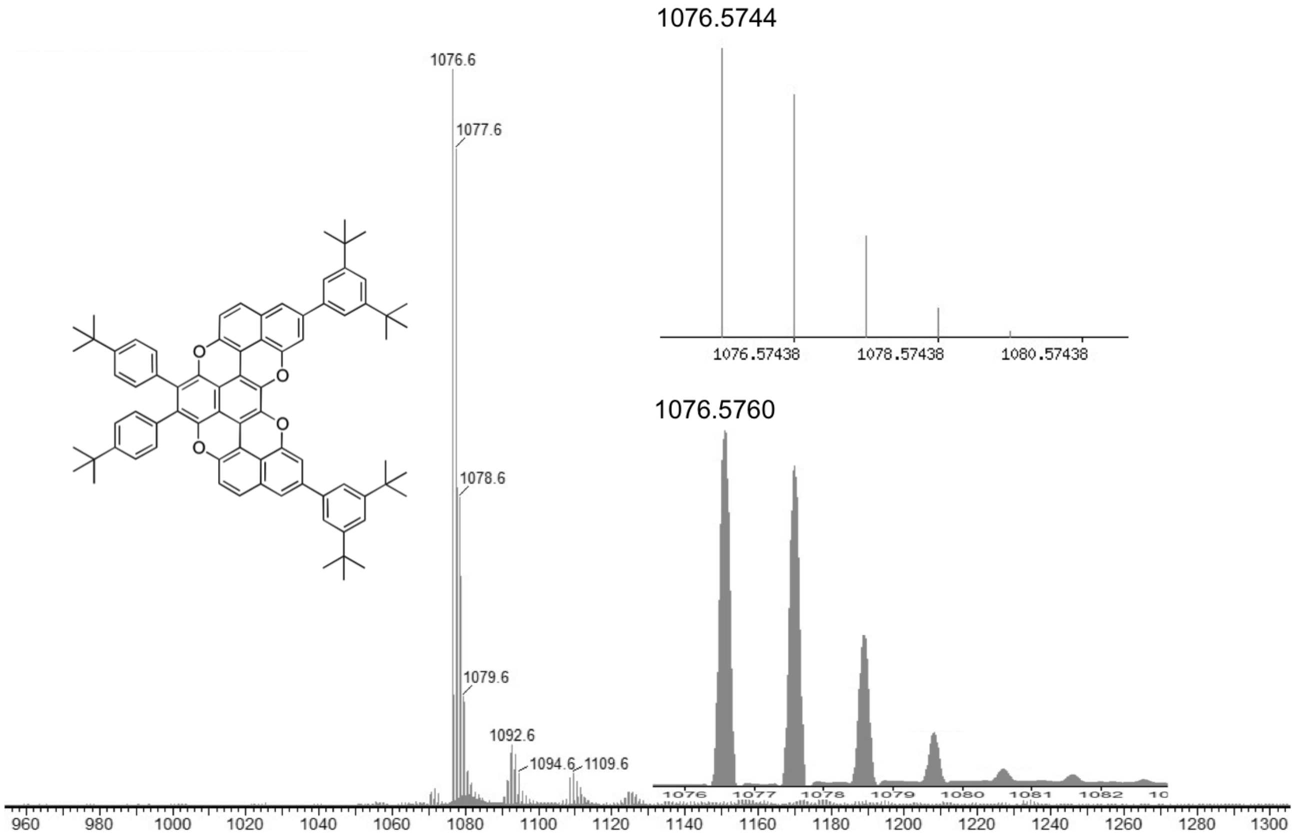

Fig. SI6. HRMS-MALDI mass spectrum of **17**$^{\mathbf{tBuPh}}$ in the positive ion mode (matrix: DTCB, N2-laser: 337 nm).

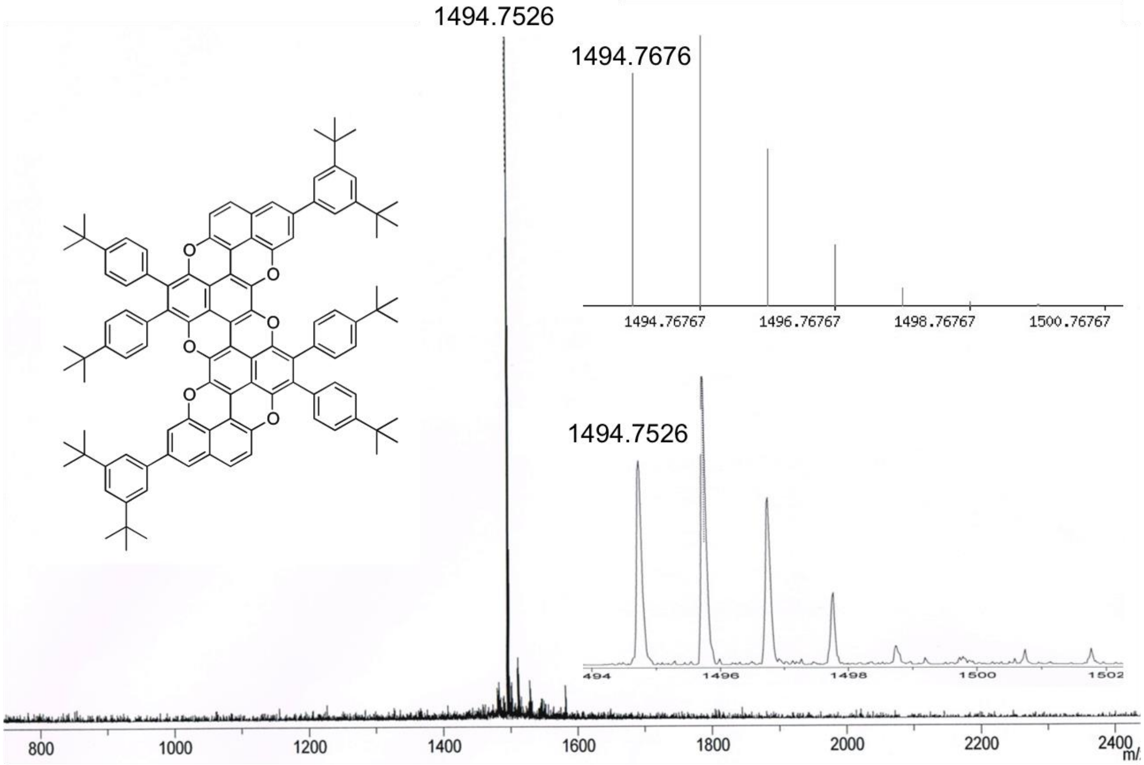

Fig. SI7. HRMS-MALDI mass spectrum of **21**$^{\mathbf{tBuPh}}$ in the positive ion mode (matrix: DTCB, N2-laser: 337 nm).

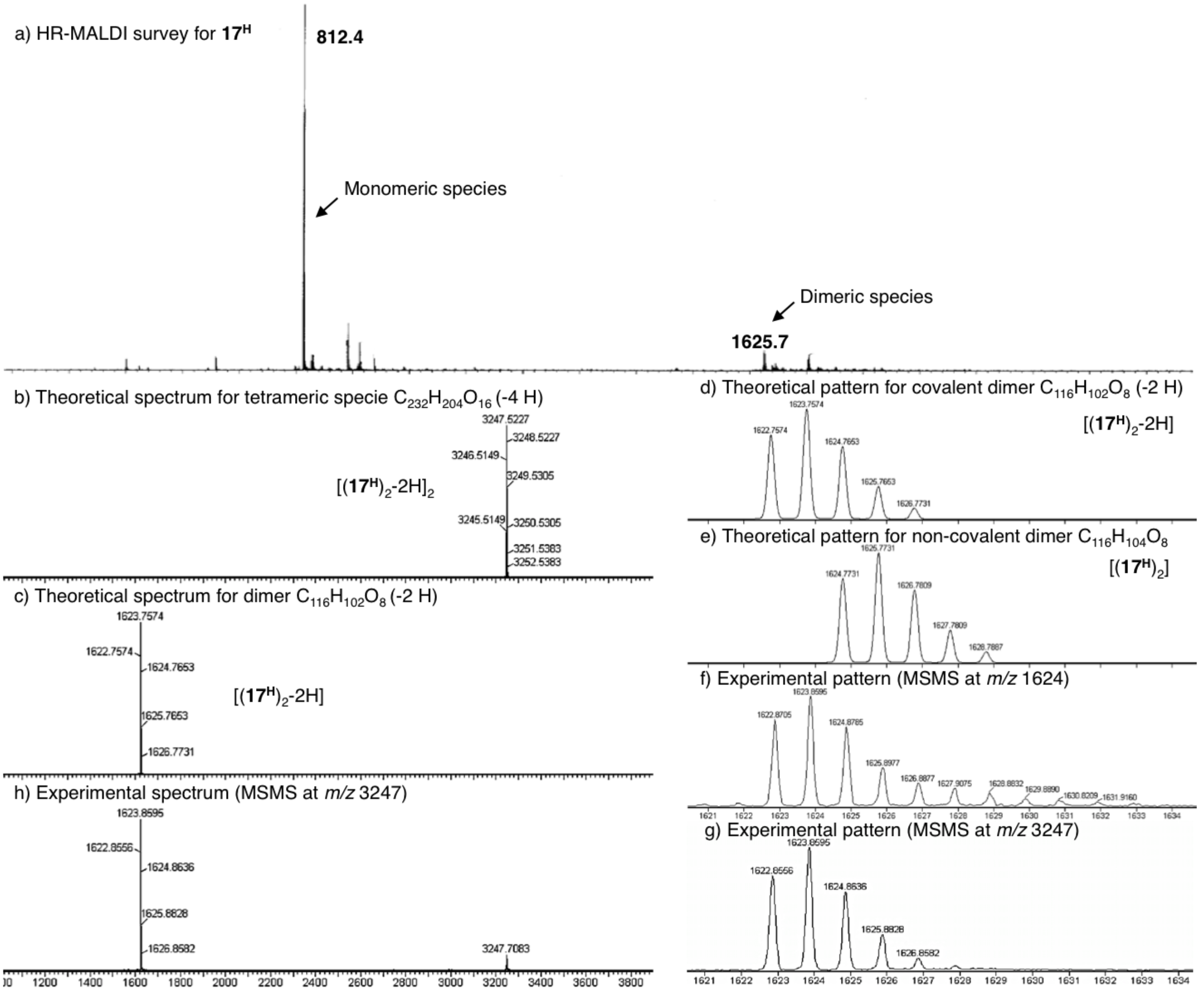

Fig. SI8. a) HRMS-MALDI spectrum for **17^H**; b-e) calculated mass spectra and isotopic patterns for the covalent and non-covalent dimeric and tetrameric species; f-g) MSMS isotopic patterns for the dimeric species; h) a MSMS spectrum.

1.5. X-Ray Analysis

Data collections for ***cis*-19^{tBuPh}** and **17^H** were performed at the X-ray diffraction beamline (XRD1) of the Elettra Synchrotron, Trieste (Italy), with a Pilatus 2M image plate detector. Complete datasets were collected at 100 K (nitrogen stream supplied through an Oxford Cryostream 700) with a monochromatic wavelength of 0.700 Å through the rotating crystal method. The crystals of the compounds were dipped in N-paratone and mounted on the

goniometer head with a nylon loop. Complete dataset for the triclinic crystal ***cis*-19^{tBuPh}** form has been obtained merging two different data collections done on the same crystal, mounted with different orientations.

The diffraction data were indexed, integrated and scaled using XDS.[3S] The structures were solved by direct methods using SIR2014,[4S] Fourier analyzed and refined by the full-matrix least-squares based on F^2 implemented in SHELXL-2014.[5S] The Coot program was used for modeling.[6S]

Anisotropic thermal motion modeling as then been applied to atoms with occupancy greater than 60% for ***cis*-19^{tBuPh}**. Several crystals have been tried for **17^{H}** and all of them show poor diffraction limit (~1.2 Å); to avoid overrefinement, thermal motion parameters have been modeled anisotropically only for oxygen atoms in this structure. Hydrogen atoms were included at calculated positions with isotropic $U_{factors}$ = 1.2 U_{eq} or Ufactors = 1.5 U_{eq} for methyl groups. Restrain on bond lengths, angles and thermal motion (DFIX, DANG and SIMU) for disordered fragments and solvent molecules have been applied. Two independent molecules for ***cis*-19^{tBuPh}** and **17^{H}** are present in the asymmetric units (ASUs).

For all the molecules, weak hydrophobic interactions keep compounds packed: several C-H - π interactions can be found and the **17^{H}** model show nicely stacked planes of almost flat molecules, with an average distance of 3.3 Å. Disordered solvent has been removed from ***cis*-19^{tBuPh}** model with the SQUEEZE routine of PLATON.[7S] The formula mass and unit-cell characteristics reported for ***cis*-19^{tBuPh}** do not take into account this disordered solvent (the contribution of this region to the scattering was estimated as ca. 198 electrons/cell, in a volume of ca. 1173 Å^3). Five isopropanol molecules have been located in molecule **17^{H}** crystal channels (aligned with crystallographic *c* axis), four of them disordered in two different orientations. Essential crystal and refinement data are reported below.

X-Ray Analysis of (±) *trans*-19^{tBuPh} CCDC Number 1415364

Empirical Formula: $C_{112}H_{118}O_6$

Formula Weight: 1560.06

Temperature: 293 K

Crystal system: monoclinic

space group: *P* 2_1/*c*

a (Å): 17.0940(3) Å, α = 90°

b (Å): 19.9020(3)

c (Å): 23.1182(5)

α (°): 90°

β (°): 130.591(1)°

γ (°): 90°

V (Å^3): 5972.4(2)

Z : 2

ρ (g·cm^{-3}): 0.868 g/cm^3

F(000): 1676

Crystal Size: 0.11 x 0.13 x 0.25 mm

Radiation: 1.54184 Å

θ Range for data collection: 4.1 to 66.6 °

Dataset: -20: 18 ; -23: 23 ; -27: 27

Total, Unique Data, R(int): 47369, 10520, 0.044

Observed data [I > 2.0 sigma(I)]: 7679

Refinement method: F^2 using SHELXL-97 software

Nref, Npar: 10520, 556

R, wR2, S: 0.0769, 0.2660, 1.06

P=(Fo^2^+2Fc^2^)/3

Max. and Av. Shift/Error: 0.00, 0.00

Min. and Max. Resd. Dens: -0.22, 0.60 Å^{-3}

X-Ray Analysis of (±) *cis*-19^{tBuPh} CCDC Number 1416668

Moiety Formula: $C_{112}H_{118}O_6$

Sum Formula: $C_{112}H_{118}O_6$

Formula weight (Da): 1560.06

Temperature (K): 100(2)

Wavelength (Å): 0.700

Crystal system: Triclinic

Space Group: *P* -1

a (Å): 21.014(4)

b (Å): 21.930(4)

c (Å): 24.956(5)

α (°): 68.14(3)

β (°): 89.85(3)

γ (°): 77.47(3)

V (Å^3): 10380(4)

Z: 4

ρ (g·cm^{-3}): 0.998

F(000): 3352

μ (mm^{-1}): 0.058

θ min,max (°):0.9, 18.6

Resolution (Å): 1.1

Total refl. Collctd: 47861

Independent refl.: 15860 [R(int) = 0.045]

Obs. Refl. [Fo>4σ(Fo)]: 10394

I/σ(I) (all data): 12.2

I/σ(I) (max resltn): 6.1

Completeness (all data): 0.97

Completeness (max resltn): 0.97

Rmerge (all data): 0.044

Rmerge (max resltn,): 0.133

Multiplicity (all data): 3.0

Multiplicity (max resltn): 3.1

Data/restraint/parameters: 15860/2625/1728

Goof: 1.734

R[I>2.0σ(I)], wR2 [I>2.0σ(I)]: 0.1718, 0.4051

R (all data), wR2 (all data): 0.2154, 0.4326

X-Ray Analysis of 17^H CCDC Number 1416669

Moiety Formula: $C_{58}H_{52}O_4 \cdot 2.5C_3H_8O$
Sum Formula: $C_{65.50}H_{72}O_{6.50}$
Formula weight (Da): 963.23
Temperature (K): 100(2)

Wavelength (Å): 0.700
Crystal system: Triclinic
Space Group: *P* -1
a (Å): 13.695(3)
b (Å): 19.931(4)
c (Å): 21.870(4)
α (°):68.32(3)
β (°):87.44(3)
γ (°):75.84(3)
V ($Å^3$): 5372(2)

Z: 4
ρ ($g \cdot cm^{-3}$): 1.191

F(000): 2068

μ (mm^{-1}): 0.072

θ min,max (°):1.0, 16.9
Resolution (Å): 1.2
Total refl. Collctd: 11488
Independent refl.: 6196 [R(int) = 0.202]
Obs. Refl. [Fo>4σ(Fo)]: 2959
I/σ(I) (all data): 3.4
I/σ(I) (max resltn): 1.8
Completeness (all data): 0.95
Completeness (max resltn): 0.93
Rmerge (all data): 0.135
Rmerge (max resltn,): 0.250
Multiplicity (all data): 1. 9
Multiplicity (max resltn): 1.9
Data/restraint/parameters: 6196/145/688
Goof: 0.983
R[I>2.0σ(I)], wR2 [I>2.0σ(I)]: 0.1614, 0.3535
R (all data), wR2 (all data): 0.2525, 0.4183

1.6. Synthesis.

2-(3,5-Di-*tert*-butylphenyl)-6-methoxynaphthalene 7

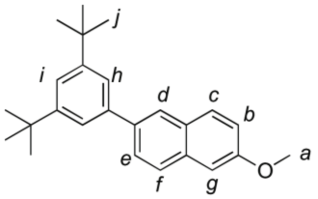

To a 5 mL microwave vial, **6** (380 mg, 1.20 mmol), 2-bromo-6-methoxynaphthalene **5** (237 mg, 1.00 mmol), Cs_2CO_3 (652 mg, 2.00 mmol) and $Pd(PPh_3)_4$ (60 mg, 0.05 mmol) were added. This was followed by the addition of toluene (2 mL), EtOH (1 mL) and DMF (1 mL) under argon and the resulting mixture was placed under microwave conditions for 1 h and at 100 °C. The resulting mixture was dissolved in EtOAc (10 mL). The organic layer was then washed with H_2O (3 × 20 mL) and the aqueous phase extracted with EtOAc (3 × 20 mL). The combined organic layers were dried over $MgSO_4$ and evaporated *in vacuo*. The residue was purified by silica gel column

chromatography (eluents: cyclohexane/CH_2Cl_2, 1:1) affording **7** as a white solid (382 mg, 91%). $C_{25}H_{30}O$; MW: 346.51 g/mol; M.p.: 170-171 °C; IR (cm^{-1}): ν 475.05, 680.38, 712.73, 823.09, 855.34, 876.08, 1029.85, 1163.23, 1204.74, 1237.53, 1361.84, 1389.18, 1454.25, 1478.73, 1591.73, 2951.67; ^{1}H-NMR (400 MHz, $CDCl_3$) δ_H: 7.96 (d, *J* = 1.37 Hz, 1H, H_c), 7.83-7.80 (m, 2H, $H_{b,f}$), 7.74-7.71 (m, 1H, H_d), 7.53 (d, *J* = 1.83 Hz, 2H, H_h), 7.45 (t, *J* = 1.83 Hz, 1H, H_i), 7.19-7.17 (m, 2H, $H_{e,g}$), 3.95 (s, 3H, H_a), 1.41 (s, 18H, H_j).); ^{13}C-NMR (100.5 MHz, $CDCl_3$) δ_C: 157.77, 151.32, 140.78, 137.83, 133.78, 129.80, 129.32, 127.23, 126.70, 125.85, 121.94, 121.42, 119.20, 105.73, 55.49, 35.18, 31.72; EI-HRMS: ($[M]^+$ $C_{25}H_{30}O^+$) calc. 346.2297, found 346.2283.

6-(3,5-Di-*tert*-butylphenyl)naphthalen-2-ol 8

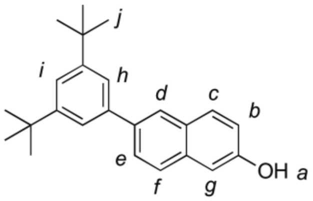

To a solution of **7** (1.34 g, 3.00 mmol) in anhydrous CH_2Cl_2 (30 mL), BBr_3 (9 mL, 9.00 mmol, 1M in CH_2Cl_2) was added at 0 °C under argon. The resulting mixture was stirred overnight at room temperature. Finally, H_2O (30 mL) was added and the solution stirred for additional 30 min. The mixture was washed with H_2O (2 × 25 mL) and the aqueous layer extracted with EtOAc (3 × 25 mL). The combined organic layers were dried over $MgSO_4$ and evaporated *in vacuo*. The residue was purified by precipitation in cold pentane affording **8** as a white solid (930 mg, 93%). $C_{24}H_{28}O$; MW: 332.48 g/mol; M.p.: 148-151 °C; IR (cm^{-1}): ν 477.65, 496.63, 678.69, 711.04, 814.72, 865.96, 897.76, 1124.07, 1181.95, 1247.29, 1362.39, 1385.84, 1453.06, 1593.05, 2948.79, 3527.24; ^{1}H-NMR (400 MHz, $CDCl_3$) δ_H: 7.95 (s, 1H, H_c), 7.83 (d, *J* = 8.93 Hz, 1H, H_b), 7.76 (d, *J* = 8.47 Hz, 1H, H_e), 7.71 (dd, *J* = 8.47, 1.83 Hz, 1H, H_d), 7.52 (d, *J* = 1.83 Hz, 2H, H_h), 7.46 (t, *J* = 1.83 Hz, 1H, H_i), 7.18 (d, *J* = 2.52 Hz, 1H, H_g), 7.13 (dd, *J* = 2.52, 8.70 Hz, 1H, H_f), 4.89 (s, 1H, H_a),

1.41 (s, 18H, H_j); ^{13}C-NMR (100.5 MHz, $CDCl_3$) δ_C: 153.44, 151.33, 140.72, 137.90, 133.76, 130.26, 129.32, 126.95, 126.85, 125.93, 121.93, 121.46, 118.20, 109.46, 35.16, 31.71; EI-HRMS: ($[M]^+$ $C_{24}H_{28}O^+$) calc. 332.2140, found 332.2133.

6,6'-Bis(3,5-di-*tert*-butylphenyl)-[1,1'-binaphthalene]-2,2'-diol 12

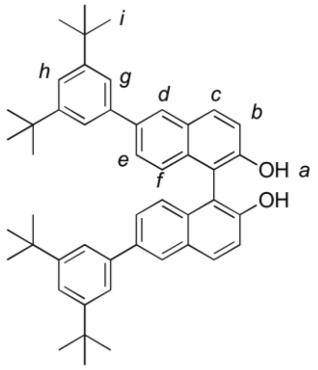

To a mixture of $CuCl_2$ (270 mg, 2.00 mmol) in anhydrous MeOH (10 mL), (±)-1-phenylethylamine (320 μL, 2.50 mmol) was added at 0 °C and the resulting blue-green mixture was degassed under argon for 30 min. A solution of **8** (332 mg, 1.00 mmol) in anhydrous CH_2Cl_2 (10 mL) was added and the resulting solution stirred overnight at room temperature. A 1M aqueous solution of HCl (10 mL) was added and the mixture extracted with CH_2Cl_2 (3 × 15 mL). The organic layer was then washed with H_2O (2 × 15 mL). The combined organic layers were dried over $MgSO_4$ and evaporated *in vacuo*. The residue was purified by silica gel column chromatography (eluents: CH_2Cl_2/cyclohexane, 1:1 with a gradient to CH_2Cl_2) affording **12** as a white solid (176 mg, 53%). $C_{48}H_{54}O_2$; MW: 662.94 g/mol; M.p.: 265-267 °C; IR (cm^{-1}): ν 520.23, 561.77, 716.12, 819.04, 825.92, 875.24, 1123.78, 1142.77, 1157.78, 1214.16, 1245.79, 1361.93, 1391.35, 1477.95, 1590.00, 2955.65, 3463.15; ^{1}H-NMR (400 MHz, C_6D_6) δ_H: 8.16 (d, J = 1.60 Hz, 2H, H_c), 7.69-7.67 (m, 6H, $H_{b,g}$), 7.58 (t, J = 1.83 Hz, 2H, H_h), 7.51 (dd, J = 1.83, 8.70 Hz, 2H, H_d), 7.37 (d, J = 8.70 Hz, 2H, H_e), 7.32 (d, J = 9.02 Hz, 2H, H_f), 4.96 (s, 2H, H_a), 1.36 (s, 36H, H_i); ^{13}C-NMR (100.5 MHz, C_6D_6) δ_C: 153.39, 151.56, 141.39, 138.95, 133.27, 131.88, 130.30, 126.91, 125.50, 122.42,

121.56, 118.59, 111.65, 35.10, 31.69 (One peak is missing due to overlap with C_6D_6); MALDI-HRMS: ($[M]^+$ $C_{48}H_{54}O_2^+$) calc. 662.4124, found 662.4117.

2,8-Bis(3,5-di-*tert*-butylphenyl)xantheno[2,1,9,8-*klmna*]xanthene 13

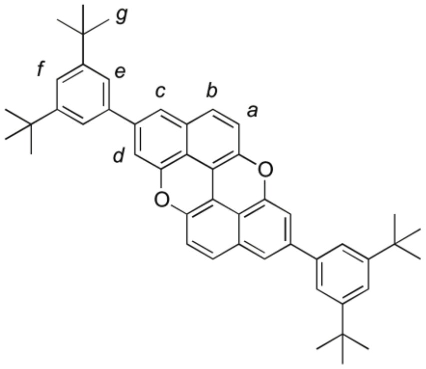

To **12** (66 mg, 0.10 mmol), CuI (57 mg, 0.30 mmol) and pivalic acid (20 mg, 0.20 mmol), anhydrous DMSO (1 mL) was added and the resulting mixture heated to 130 °C for 2 h under open-air condition. The solution was filtered, washed with CH_2Cl_2 and evaporated under vacuum. The residue was purified by silica gel column chromatography (eluents: CH_2Cl_2/cyclohexane, 1:1) followed by precipitation in MeOH affording **13** as a yellow solid (62 mg, 94%). $C_{48}H_{50}O_2$; MW: 658.91 g/mol; M.p.: > 300 °C; IR (cm^{-1}): ν 711.84, 852.97, 1064.13, 1077.53, 1225.18, 1246.56, 1261.27, 1361.2, 1458.34, 1579.81, 1594.21, 1631.26, 2854.10, 2923.29, 2957.97; UV-vis (CH_2Cl_2, r.t.): λ_{max} (ε_{max}, mol^{-1} L cm^{-1}) 256 (63329), 282 (104172), 340 (11228), 396 (7691), 420 (15427), 448 (20566); ^{1}H-NMR (400 MHz, C_6D_6) δ_H: 7.65 (d, J = 1.83 Hz, 4H, Ar*H*), 7.60 (t, J = 1.83 Hz, 2H, Ar*H*), 7.40 (d, J = 1.14 Hz, 2H, Ar*H*), 7.23 (d, J = 1.37 Hz, 2H, Ar*H*), 7.03 (d, J = 8.93 Hz, 2H, Ar*H*), 6.81 (d, J = 8.93 Hz, 2H, Ar*H*), 1.36 (s, 36H, C*H3*); ^{13}C-NMR (100.5 MHz, C_6D_6) δ_C: 153.61, 151.61, 144.79, 142.63, 141.38, 132.04, 126.94, 122.19, 121.93, 121.14, 118.94, 117.96, 111.99, 109.51, 35.11, 31.69; MALDI-HRMS: ($[M]^+$ $C_{48}H_{50}O_2^+$) calc. 658.3811, found 658.3813.

3-(Benzyloxy)-6'-(3,5-di-*tert*-butylphenyl)-2,2'-dimethoxy-1,1'-binaphthalene 10[H]

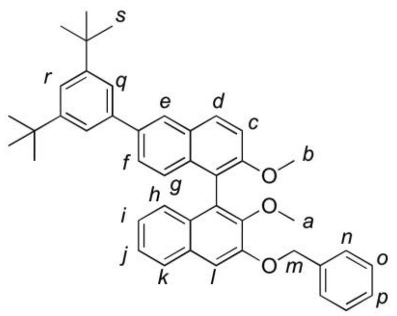

To a mixture of $CuCl_2$ (537 mg, 4.00 mmol) in anhydrous MeOH (25 mL), (±)-1-phenylethylamine (640 μL, 5.00 mmol) was added at 0 °C and the resulting blue-green mixture degassed under argon during 30 min. A solution of **8** (664 mg, 2.00 mmol) and 3-(benzyloxy)naphthalen-2-ol (600 mg, 2.40 mmol) in anhydrous CH_2Cl_2 (25 mL) was added and the resulting solution stirred overnight at room temperature. A 1M aqueous solution of HCl (20 mL) was added and the solution stirred for another 30 min. CH_2Cl_2 (20 mL) was added and the mixture washed with H_2O (3 × 25 mL). The aqueous phase was then extracted with CH_2Cl_2 (2 × 25 mL). The combined organic layers were dried over $MgSO_4$ and evaporated *in vacuo*. The residue was purified by silica gel column chromatography (eluents: CH_2Cl_2/cyclohexane, 1:1 with a gradient to CH_2Cl_2) affording **9[H]** in a mixture of the three possible cross-coupling products as a white solid (1.05 g – no yield reported). $C_{41}H_{40}O_3$; MW: 580.75 g/mol; M.p.: 166-168 °C; MALDI-HRMS: ($[M]^+$ $C_{41}H_{40}O_3^+$) calc. 580.2977, found 580.2973. To a solution of the obtained mixture (970 mg, 1.68 mmol) and K_2CO_3 (3.60 g, 26.08 mmol) in anhydrous acetone (50 mL), MeI (2.16 mL, 34.68 mmol) was added under argon at room temperature. The resulting mixture was refluxed during 2 h and further stirred overnight at room temperature. A 1M aqueous solution of HCl (30 mL) and EtOAc (30 mL) were added to the solution. The mixture was washed with H_2O (2 × 50 mL) and the aqueous layer extracted with EtOAc (2 × 50 mL). The combined organic layers were dried over $MgSO_4$ and evaporated *in vacuo*. The residue was purified by silica gel column chromatography (eluents:

CH_2Cl_2/pentane, 1:1) affording **10^H^** as a white solid (560 mg, 46% over 2 steps). $C_{43}H_{44}O_3$; MW: 608.81 g/mol; M.p.: 105-107 °C; IR (cm^{-1}): ν 698.00, 712.20, 735.65, 825.10, 873.80, 1019.03, 1037.39, 1046.83, 1092.93, 1114.60, 1151.01, 1248.25, 1267.74, 1329.84, 1362.77, 1423.56, 1442.81, 1462.62, 1590.50, 2960.97; ^{1}H-NMR (400 MHz, CD_2Cl_2) δ_H: 8.19-8.17 (m, 2H, $H_{c,d}$), 7.88 (d, J = 8.24 Hz, 1H, H_k), 7.66-7.58 (m, 6H, $H_{e,g,n,q}$), 7.55-7.41 (m, 6H, $H_{i,l,o,p,r}$), 7.28 (d, J = 8.70 Hz, 1H, H_f), 7.22-7.14 (m, 2H, $H_{h,j}$), 5.39 (s, 2H, H_m), 3.88 (s, 3H, H_b), 3.74 (s, 3H, H_a), 1.48 (s, 18H, H_s); ^{13}C-NMR (100.5 MHz, CD_2Cl_2) δ_C: 155.24, 152.08, 151.76, 148.25, 140.91, 137.96, 137.43, 133.51, 131.80, 130.30, 129.69, 129.46, 129.06, 128.46, 128.02, 127.20, 127.08, 126.88, 126.29, 125.93, 125.79, 125.67, 124.55, 122.20, 121.79, 119.12, 114.19, 109.01, 70.86, 60.92, 56.68, 35.35, 31.77; MALDI-HRMS: ($[M]^+$ $C_{43}H_{44}O_3^+$) calc. 608.3290, found 608.3304.

6'-(3,5-Di-*tert*-butylphenyl)-2,2'-dimethoxy-[1,1'-binaphthalen]-3-ol 11^H^

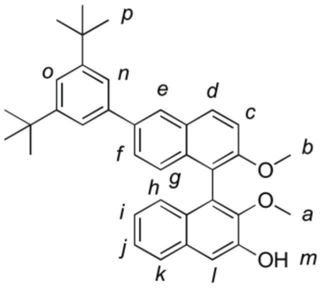

To a suspension of Pd/C (700 mg, 6.58 mmol) in $CHCl_3$ (20 mL) at 0 °C under argon, a solution of **10^H^** (400 mg, 0.66 mmol) in $CHCl_3$ (20 mL) and AcOH (2 mL) was added. The solution was degassed with H_2 for 40 min and the resulting dark solution stirred during 24 h at room temperature, under H_2 atmosphere. The solution was filtered and the solvent evaporated *in vacuo*. The residue was purified by silica gel column chromatography (eluents: CH_2Cl_2/pentane, 1:1 with gradient to CH_2Cl_2) affording **11^H^** as a white solid (310 mg, 91%). $C_{36}H_{38}O_3$; MW: 518.69 g/mol; M.p.: 226-228 °C; IR (cm^{-1}): ν 694.64, 712.44, 736.29, 825.71, 873.51, 1001.14, 1040.77, 1087.79,

1109.21, 1147.63, 1171.44, 1203.33, 1244.67, 1267.09, 1362.41, 1427.7, 1450.89, 1464.23, 1589.75, 2961.05, 3508.60; ^{1}H-NMR (400 MHz, CD_2Cl_2) δ_H: 8.14 (d, J = 8.16 Hz, 1H, H_c), 8.09 (d, J = 1.83 Hz, 1H, H_d), 7.76 (d, J = 8.24 Hz, 1H, H_k), 7.55-7.52 (m, 2H, $H_{e,g}$), 7.50 (d, J = 1.83 Hz, 2H, H_n), 7.45 (t, J = 1.83 Hz, 1H, H_o), 7.42 (s, 1H, H_l), 7.37-7.33 (m, 1H, H_i), 7.17 (d, J = 8.70 Hz, 1H, H_f), 7.12-7.04 (m, 2H, $H_{h,j}$), 6.19 (s, 1H, H_m), 3.85 (s, 3H, H_b), 3.44 (s, 3H, H_a), 1.38 (s, 18H, H_p); ^{13}C-NMR (100.5 MHz, CD_2Cl_2) δ_C: 155.39, 151.75, 148.38, 146.56, 140.66, 138.06, 133.29, 132.00, 130.84, 129.66, 129.14, 127.30, 126.99, 126.25, 125.77, 125.69, 125.55, 124.40, 124.10, 122.10, 121.82, 118.28, 114.04, 109.95, 61.12, 56.68, 35.30, 31.65; MALDI-HRMS: ($[M]^+$ $[C_{36}H_{38}O_3]^+$) calc. 518.2821, found 518.2832.

2-(3,5-Di-*tert*-butylphenyl)-10-(6-(3,5-di-*tert*-butylphenyl)-2-methoxynaphthalen-1-yl)-11-methoxyxantheno[2,1,9,8-*klmna*]xanthene 15^H

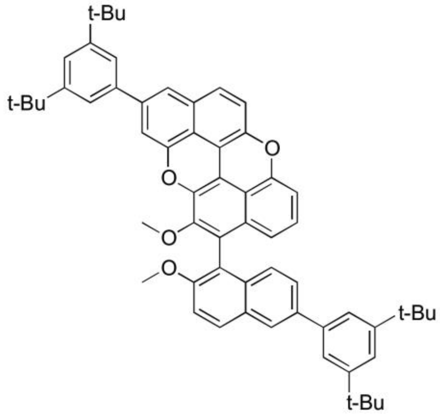

To a mixture of $CuCl_2$ (180 mg, 1.34 mmol) in anhydrous MeOH (15 mL), (±)-1-phenylethylamine (215 μL, 1.66 mmol) was added at 0 °C and the resulting blue-green mixture degassed under argon for 30 min. This was followed by the addition of a solution of **11^H** (345 mg, 0.66 mmol) and **8** (332 mg, 1.00 mmol) in anhydrous CH_2Cl_2 (15 mL), and the resulting solution was stirred overnight at room temperature. A 1M aqueous solution of HCl (20 mL) was added and the solution stirred for additional 30 min. CH_2Cl_2 (20 mL) was added and the mixture washed with H_2O (3 × 20 mL). The aqueous

phase was then extracted with CH_2Cl_2 (3 × 25 mL). The combined organic layers were dried over $MgSO_4$ and evaporated *in vacuo*. The residue was purified by silica gel column chromatography (eluents: CH_2Cl_2/pentane, 1:1 with a gradient to CH_2Cl_2) affording the mixture of the 3 possible compounds that were used without further purification. To the obtained mixture (140 mg, 0.16 mmol), CuI (95 mg, 0.49 mmol) and pivalic acid (34 mg, 0.33 mmol), anhydrous DMSO (2 mL) was added and the resulting mixture heated to 145 °C for 4 h. The resulting crude was filtered, washed with CH_2Cl_2 and evaporated *in vacuo*. The black residue was purified by silica gel column chromatography (eluents: CH_2Cl_2/cyclohexane, 3:7 with a gradient to CH_2Cl_2) followed by precipitation in MeOH affording **15^{H}** as a yellow solid (30 mg, 5% over two steps). $C_{60}H_{60}O_4$; MW: 845.12 g/mol; M.p.: decomposition before reaching melting point; IR (cm^{-1}): ν 799.42, 848.42, 1054.78, 1070.27, 1141.81, 1209.70, 1235.49, 1248.53, 1328.73, 1362.15, 1403.96, 1453.35, 1503.83, 1593.57, 1629.45, 2961.06; UV-vis (CH_2Cl_2, r.t.): λ_{max} (ε_{max}, mol^{-1} L cm^{-1}) 227 (64248), 262 (82561), 396 (7004), 419 (12503), 447 (15509); ^{1}H-NMR (400 MHz, C_6D_6) δ_H: 8.25 (s, 1H, Ar*H*), 7.83 (d, *J* = 8.93 Hz, 1H, Ar*H*), 7.74-7.59 (m, 8H, Ar*H*), 7.42 (d, *J* = 0.69 Hz, 1H, Ar*H*), 7.28 (d, *J* = 0.92 Hz, 1H, Ar*H*), 7.10 (d, *J* = 9.16 Hz, 1H, Ar*H*), 7.04 (d, *J* = 8.93 Hz, 1H, Ar*H*), 6.82 (d, *J* = 8.93 Hz, 1H, Ar*H*), 6.74-6.60 (m, 3H, Ar*H*), 3.57 (s, 3H, OCH_3), 3.37 (s, 3H, OCH_3), 1.37-1.34 (m, 36H, CCH_3); ^{13}C-NMR (100.5 MHz, C_6D_6) δ_C: 155.30, 153.29, 153.25, 151.59, 151.51, 147.41, 144.77, 142.51, 141.77, 141.32, 139.55, 138.76, 133.54, 132.91, 131.88, 130.23, 130.06, 128.90, 126.94, 126.62, 126.30, 125.12, 122.56, 122.17, 121.92, 121.42, 121.07, 119.11, 119.09, 118.96, 118.89, 117.96, 114.18, 113.56, 112.13, 109.50, 108.14, 60.83, 55.97, 35.11, 31.71, 31.68 (one aromatic peak is missing probably due to overlap with the solvent and one *C*CH_3 is missing due to overlap); MALDI-HRMS: ($[M]^+$ $[C_{60}H_{60}O_4]^+$) calc. 844.4492, found 844.4478.

8-(3,5-Di-*tert*-butylphenyl)-4-(6-(3,5-di-*tert*-butylphenyl)-2-hydroxynaphthalen-1-yl)xantheno[2,1,9,8-*klmna*]xanthen-5-ol 16^{H}

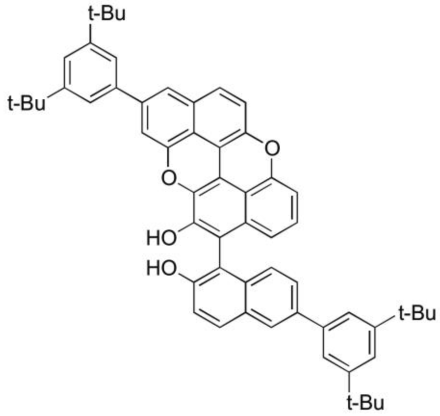

To a solution of **15^{H}** (30 mg, 0.036 mmol) in anhydrous CH_2Cl_2 (10 mL), BBr_3 (110 μL, 0.11 mmol, 1M in CH_2Cl_2) was added at 0 °C under argon. The resulting mixture was stirred overnight at room temperature. As the starting material was still present, BBr_3 (200 μL, 0.20 mmol, 1M in CH_2Cl_2) was added and the solution stirred 3 h. H_2O (10 mL) was added and the solution stirred 30 min. The mixture was washed with H_2O (2 x 15 mL) and the aqueous layer extracted with CH_2Cl_2 (2 × 20 mL). The combined organic layers were dried over $MgSO_4$ and evaporated *in vacuo*. The residue was purified by silica gel column chromatography (eluents: CH_2Cl_2/cyclohexane, 1:1) affording **16^{H}** as a yellow solid (24 mg, 82%). $C_{58}H_{56}O_4$; MW: 817.06 g/mol; M.p.: decomposition before reaching melting point; IR (cm^{-1}): ν 712.44, 751.45, 852.12, 873.63, 1132.49, 1170.96, 1232.38, 1248.44, 1362.42, 1441.74, 1476.95, 1508.12, 1594.13, 1630.75, 2960.86, 3062.56, 3492.89; UV-vis (CH_2Cl_2, r.t.): λ_{max} (ε_{max}, mol^{-1} L cm^{-1}) 227 (64076), 262 (83784), 396 (6534), 419 (12494), 447 (15186); ^{1}H-NMR (400 MHz, CD_2Cl_2) δ_H: 8.09-8.03 (m, 2H, Ar*H*), 7.64 (dd, *J* = 1.83 Hz, 8.70 Hz, 1H, Ar*H*), 7.63-7.38 (m, 10H, Ar*H*), 7.04-7.01 (m, 2H, Ar*H*), 6.92 (t, *J* = 8.24 Hz, 1H, Ar*H*), 6.56-6.40 (m, 2H, Ar*H*), 5.56-5.52 (m, 2H, O*H*), 1.38 (s, 36H, CCH_3); ^{13}C-NMR (100.5 MHz, CD_2Cl_2) δ_C: 171.10, 153.07, 152.73, 152.46, 151.77, 144.98, 142.18, 140.58, 140.19, 138.24, 132.61, 132.04, 131.83, 131.67, 130.12, 128.97, 127.93, 127.62, 126.73, 124.91, 122.34, 122.09, 121.90, 121.85, 120.57, 119.24, 118.68, 118.20, 117.57,

116.88, 114.18, 111.75, 110.90, 109.59, 107.97, 35.29, 31.65 (three aromatic C peaks are missing probably due to overlap and the *C*CH_3 are overlapping); MALDI-HRMS: ([M]$^+$ [$C_{58}H_{56}O_4$]$^+$) calc. 816.4179, found 816.4168.

2,13-bis(3,5-di-*tert*-butylphenyl)-tetraoxa-pentaphenopentaphene 17^{H}

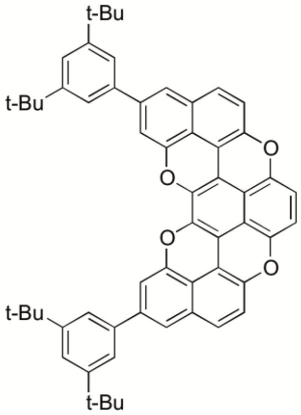

To **16^{H}** (20 mg, 0.024 mmol), CuI (15 mg, 0.073 mmol) and pivalic acid (5 mg, 0.049 mmol), anhydrous DMSO (2 mL) was added and the resulting mixture heated at 140 °C for 4 h. The resulting crude was filtered, washed with CH_2Cl_2 and evaporated *in vacuo*. The dark-red residue was purified by silica gel column chromatography (eluents: CH_2Cl_2) followed by precipitation in MeOH, affording **17^{H}** as a red solid (6 mg, 31%). $C_{58}H_{52}O_4$; MW: 813.03 g/mol; M.p. > 250 °C; IR (cm^{-1}): ν 699.92, 737.82, 802.19, 858.03, 1072.12, 1261.28, 1362.82, 1461.87, 1595.20, 1727.31, 2851.98, 2921.51, 2956.20; UV-vis (CH_2Cl_2, r.t.): λ_{max} (ε_{max}, mol^{-1} L cm^{-1}) 226 (37924), 262 (42227), 495 (2710), 542 (1691); MALDI-HRMS: ([M]$^+$ $C_{58}H_{52}O_4^+$) calc. 812.3866, found 812.3848.

6,6'''-Bis(3,5-di-*tert*-butylphenyl)-2,2',2''',3''-tetramethoxy-[1,1':4',1'':4'',1'''-quaternaphthalene]-2'',3'-diol 18^{H}

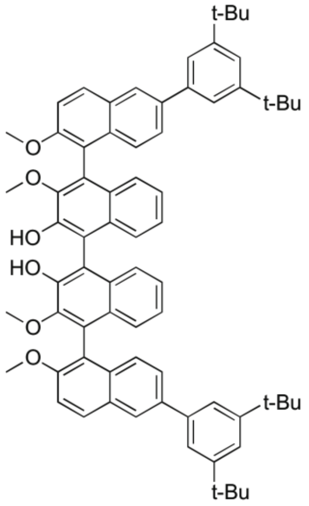

To a mixture of $CuCl_2$ (135 mg, 1.00 mmol) in anhydrous MeOH (10 mL), (±)-1-phenylethylamine (161 μL, 1.25 mmol) was added at 0 °C and the resulting blue-green mixture degassed under argon for 30 min. This was followed by the addition of a solution of **11^{H}** (250 mg, 0.48 mmol) in anhydrous CH_2Cl_2 (10 mL), and the resulting solution was stirred overnight at room temperature. A 1M aqueous solution of HCl (10 mL) was added and the solution stirred for additional 30 min. CH_2Cl_2 (20 mL) was added and the mixture washed with H_2O (3 × 20 mL). The aqueous phase was then extracted with CH_2Cl_2 (2 × 25 mL). The combined organic layers were dried over $MgSO_4$ and evaporated *in vacuo*. The residue was precipitated in MeOH affording **18^{H}** as a white solid as a mixture of diastereoisomers (210 mg, 85%). $C_{72}H_{74}O_6$; MW: 1035.35 g/mol; M.p.: decomposition before reaching melting point; IR (cm^{-1}): ν 481.81, 712.46, 758.58, 799.43, 824.44, 873.74, 906.74, 1002.27, 1055.69, 1087.98, 1110.99, 1153.14, 1249.29, 1269.39, 1345.19, 1361.29, 1398.73, 1448.84, 1589.45, 2957.92, 3492.50; ^{1}H-NMR (400 MHz, CD_2Cl_2) δ_H: 8.20 (d, J = 8.93 Hz, 2H, ArH), 8.14 (d, J = 1.83 Hz, 1H, ArH), 7.64-7.61 (m, 4H, ArH), 7.54 (d, J = 1.60 Hz, 4H, ArH), 7.47-7.14 (m, 12H, ArH), 6.26 (s, 2H, OH), 3.97 (s, 6H, OCH_3), 3.60 (s, 6H, OCH_3), 1.39 (s, 36H, CCH_3); ^{13}C-NMR (100.5 MHz, CD_2Cl_2) δ_C: 155.43, 151.69, 146.69, 146.55, 140.66, 138.01, 133.37, 131.12, 130.82,

129.60, 129.33, 127.36, 126.25, 126.04, 125.94, 125.38, 124.84, 124.36, 122.11, 121.77, 118.31, 115.10, 114.00, 61.23, 56.68, 35.25, 31.61 (one peak is missing probably due to overlap); MALDI-HRMS: ([M]$^+$ [$C_{72}H_{74}O_6$]$^+$) calc. 1034.5485, found 1034.5461.

4,10-Bis(6-(3,5-di-*tert*-butylphenyl)-2-methoxynaphthalen-1-yl)-5,11-dimethoxyxantheno[2,1,9,8-*klmna*]xanthene 19^H

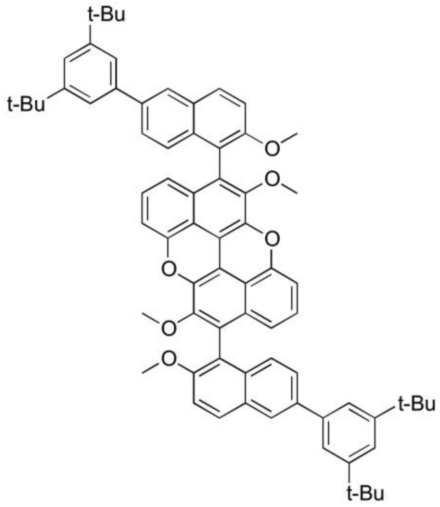

To a solution of **18^H** (210 mg, 0.20 mmol), CuI (115 mg, 0.61 mmol) and pivalic acid (42 mg, 0.41 mmol), anhydrous DMSO (2 mL) was added and the resulting mixture heated at 145 °C for 5 h. The resulting crude was filtered, washed with CH_2Cl_2 and evaporated *in vacuo*. The residue was purified by silica gel column chromatography (eluents: CH_2Cl_2/pentane, 3:7 with a gradient of 1:1) affording **19^H** (118 mg, 57%). $C_{72}H_{70}O_6$; MW: 1031.32 g/mol; M.p. > 250 °C; IR (cm^{-1}): ν 712.60, 756.76, 800.36, 834.51, 873.15, 1037.41, 1073.14, 1148.32, 1219.16, 1252.64, 1270.94, 1344.99, 1361.26, 1445.17, 1461.74, 1592.21, 2900.30, 2964.20; UV-vis (CH_2Cl_2, r.t.): λ_{max} (ε_{max}, mol^{-1} L cm^{-1}) 231 (101091), 291 (116348), 393 (9624), 416 (19586), 444 (22985);

(±) *Trans*-like diastereoisomer

^{1}H-NMR (400 MHz, CD_2Cl_2) δ_H: 8.12-8.10 (m, 4H, Ar*H*), 7.63 (d, *J* = 8.93 Hz, 2H, Ar*H*), 7.55-7.53 (m, 6H, Ar*H*), 7.46 (s, 2H, Ar*H*), 7.38 (d, *J* = 8.70 Hz, 2H, Ar*H*), 6.97 (t, *J* = 8.47 Hz, 2H, Ar*H*), 6.74 (d, *J* = 7.56 Hz, 2H, Ar*H*), 6.39 (d, *J* = 8.47 Hz, 2H, Ar*H*), 3.91 (s, 6H, OC*H*$_3$), 3.73 (s, 6H, OC*H*$_3$), 1.40 (s, 36H, CC*H*$_3$); ^{13}C-NMR

(100.5 MHz, CD_2Cl_2) δ_C: 155.15, 152.71, 151.74, 146.96, 140.71, 139.29, 137.97, 133.02, 132.41, 130.50, 129.74, 128.02, 127.24, 126.31, 125.51, 124.88, 122.10, 121.78, 119.01, 118.43, 118.10, 114.18, 113.36, 108.47, 61.33, 56.78, 35.30, 31.66; MALDI-HRMS: ($[M]^+$ $[C_{72}H_{70}O_6]^+$) calc. 1030.5172, found 1030.5190.

(±) *Cis*-like diastereoisomer

^1H-NMR (400 MHz, CD_2Cl_2) 8.13-8.10 (m, 4H, Ar*H*), 7.63 (dd, *J* = 1.60, 8.93 Hz, 2H, Ar*H*), 7.56-7.53 (m, 6H, Ar*H*), 7.47 (m, 2H, Ar*H*), 7.39 (d, *J* = 8.70 Hz, 2H, Ar*H*), 6.97 (t, *J* = 8.47 Hz, 2H, Ar*H*), 6.75 (d, *J* = 7.56 Hz, 2H, Ar*H*), 6.40 (d, *J* = 8.47 Hz, 2H, Ar*H*), 3.91 (s, 6H, OCH_3), 3.74 (s, 6H, OCH_3), 1.40 (s, 36H, CCH_3); ^{13}C-NMR (100.5 MHz, CD_2Cl_2) δ_C: 155.18, 152.73, 151.75, 146.97, 140.73, 139.30, 138.00, 133.03, 132.42, 130.50, 129.75, 128.03, 127.23, 126.34, 125.52, 124.90, 122.11, 121.79, 119.03, 118.44, 118.12, 114.22, 113.35, 108.48, 61.33, 56.80, 35.31, 31.67; MALDI-HRMS: ($[M]^+$ $[C_{72}H_{70}O_6]^+$) calc. 1030.5172, found 1030.5204.

4,10-Bis(6-(3,5-di-tert-butylphenyl)-2-hydroxynaphthalen-1-yl)xantheno[2,1,9,8-klmna]xanthene-5,11-diol 20^H

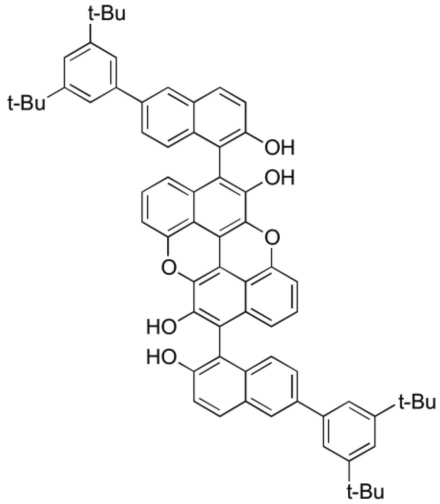

To a solution of **19^H** (60 mg, 0.058 mmol) in anhydrous CH_2Cl_2 (10 mL), BBr_3 (290 μL, 0.29 mmol, 1M in CH_2Cl_2) was added at 0 °C under argon. The resulting mixture was stirred overnight at room temperature. H_2O (10 mL) was then added and the solution stirred for 30 min. The mixture was washed with H_2O (2 × 15 mL) and the aqueous layer extracted with CH_2Cl_2 (2 × 15 mL). The combined

organic layers were dried over $MgSO_4$ and evaporated *in vacuo*. The residue was purified by precipitation in cold pentane affording **20^H** as a green solid (44 mg, 78%). $C_{68}H_{62}O_6$; MW: 975.22 g/mol; M.p. > 250 °C; IR (cm^{-1}): ν 713.11, 746.38, 798.14, 845.08, 874.55, 1135.10, 1216.03, 1245.08, 1362.33, 1394.11, 1434.65, 1462.42, 1477.96, 1508.06, 1594.19, 2961.87, 3520.70; UV-vis (CH_2Cl_2, r.t.): λ_{max} (ε_{max}, mol^{-1} L cm^{-1}) 229 (99459), 260 (103430), 288 (57196), 334 (13185), 391 (8918), 415 (16727), 443 (18128); ^{1}H-NMR (400 MHz, THF-d_6) 8.30 (s, 2H, O*H*), 8.03 (d, *J* = 1.60 Hz, 2H, Ar*H*), 7.96 (s, 2H, O*H*), 7.93 (d, *J* = 8.93 Hz, 2H, Ar*H*), 7.54-7.52 (m, 6H, Ar*H*), 7.44 (t, *J* = 1.60 Hz, 2H, Ar*H*), 7.34 (d, *J* = 8.93 Hz, 2H, Ar*H*), 7.28 (d, *J* = 8.93 Hz, 2H, Ar*H*), 6.92-6.88 (m, 2H, Ar*H*), 6.62 (d, *J* = 7.56 Hz, 2H, Ar*H*), 6.38 (d, *J* = 8.70 Hz, 2H, Ar*H*), 1.39 (s, 36H, C*H_3*); ^{13}C-NMR (100.5 MHz, THF-d_6) δ_C: 154.35, 153.31, 151.51, 145.79, 141.70, 137.60, 136.62, 134.01, 133.52, 130.51, 130.12, 127.98, 126.82, 126.54, 125.61, 122.24, 121.43, 119.75, 118.74, 116.59, 115.78, 114.17, 112.78, 106.95, 35.40, 31.71; MALDI-HRMS: ($[M]^+$ $[C_{68}H_{62}O_6]^+$) calc. 974.4546, found 974.4564.

2,12-bis(3,5-di-*tert*-butylphenyl)-hexaoxa-naphtotetraphenopyranthrene 21^H

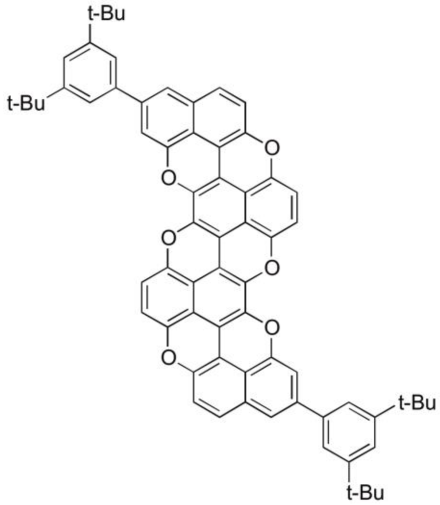

To a solution of **20^H** (40 mg, 0.040 mmol), CuI (47 mg, 0.25 mmol) and pivalic acid (17 mg, 0.16 mmol), anhydrous DMSO (2 mL) was added and the resulting mixture heated at 145 °C for 3 h. The resulting crude was filtered, abundantly washed with CH_2Cl_2, EtOAc, MeOH and pyridine and evaporated *in vacuo*. The black residue was

purified by silica gel column chromatography (eluents: CH_2Cl_2 to EtOAc) affording desired **21^{H}** as a black residue (14 mg, 36%). $C_{68}H_{54}O_6$; MW: 967.15 g/mol; M.p. > 250 °C; IR (cm^{-1}): ν 534.90, 711.46, 736.71, 804.89, 873.88, 1073.44, 1246.92, 1261.46, 1362.14, 1454.82, 1505.21, 1592.70, 1725.60, 2852.57, 2922.45, 2956.79; UV-vis (CH_2Cl_2, r.t.): λ_{max} (ε_{max}, mol^{-1} L cm^{-1}) 263 (16070); MALDI-HRMS: ([M]$^+$ $C_{68}H_{54}O_6^+$) calc. 966.3920, found 966.3935.

3-(Benzyloxy)-6,7-dibromonaphthalen-2-ol 2

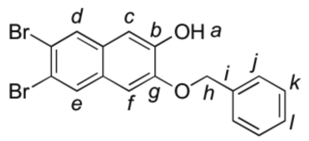

To a solution of **1** (1.59 g, 5.00 mmol) in anhydrous DMF (10 mL), $NaHCO_3$ (420 g, 5.00 mmol) was added under argon at room temperature. The resulting mixture was heated at 100 °C for 1 h. Benzyl bromide (535 μL, 4.50 mmol) was then added and the resulting mixture heated at 100 °C overnight. After cooling down, the mixture was washed with H_2O (3 × 10 mL). The combined aqueous layers were then extracted with EtOAc (3 x 10 mL). The combined organic layers were dried over $MgSO_4$ and evaporated *in vacuo*. The residue was purified by silica gel column chromatography (eluents: CH_2Cl_2) affording **2** as a white solid (800 mg, 39%). $C_{17}H_{12}Br_2O_2$; MW: 408.08 g/mol; M.p.: 164-165 °C; IR (cm^{-1}): ν 539.45, 695.35, 736.30, 887.28, 941.85, 1023.66, 1104.08, 1150.63, 1250.59, 1292.16, 1352.4, 1383.68, 1403.54, 1453.27, 1498.08, 3515.9; ^{1}H-NMR (400 MHz, $CDCl_3$) δ_H: 7.94 (s, 2H, $H_{d,e}$), 7.47-7.40 (m, 5H, $H_{j,k,l}$), 7.16 (s, 1H, H_f), 7.08 (s, 1H, H_c), 6.03 (s, 1H, H_a), 5.23 (s, 2H, H_h); ^{13}C-NMR (100.5 MHz, $CDCl_3$) δ_C: 147.55, 146.98, 135.45, 130.87, 130.66, 129.82, 129.05, 128.95, 128.17, 120.19, 119.52, 108.68, 106.00, 71.35 (one peak is missing probably due to overlap); ESI-HRMS: ([M+H]$^+$ $C_{17}H_{13}Br_2O_2^+$) calc. 406.9277, found 406.9272.

3-(Benzyloxy)-6,7-bis(4-(*tert*-butyl)phenyl)naphthalen-2-ol 4^{tBuPh}

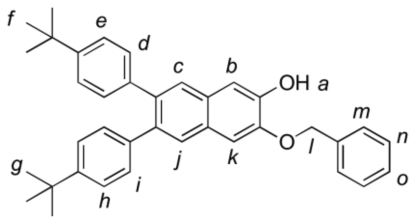

To a 5 mL microwave vial were added **2** (204 mg, 0.50 mmol), (4-(*tert*-butyl)phenyl)boronic acid **3** (196 mg, 1.10 mmol), Cs_2CO_3 (489 mg, 1.50 mmol) and $Pd(PPh_3)_4$ (58 mg, 0.05 mmol). This was followed by the addition of toluene (2 mL), EtOH (1 mL) and DMF (1 mL) under argon and the resulting reaction mixture was heated under microwave conditions for 1 h at 100 °C. The resulting mixture was then dissolved in CH_2Cl_2 (5 mL) and the organic layer washed with H_2O (3 × 10 mL). The resulting aqueous phase was then extracted with CH_2Cl_2 (3 × 10 mL). The combined organic layers were dried over $MgSO_4$ and evaporated *in vacuo*. The residue was purified by silica gel column chromatography (eluents: cyclohexane/CH_2Cl_2, 1:1) affording **4**$^{\mathbf{tBuPh}}$ as a white solid (255 mg, 99%). $C_{37}H_{38}O_2$; MW: 514.29 g/mol; ^{1}H-NMR (400 MHz, $CDCl_3$) δ_H: 7.71-7.70 (d, 2H, $H_{c,j}$), 7.51-7.37 (m, 5H, $H_{m,n,o}$), 7.31 (s, 1H, H_b), 7.25-7.23 (m, 5H, $H_{e,h,k}$), 7.14-7.11 (m, 4H, $H_{d,i}$), 5.99 (s, 1H, H_a), 5.27 (s, 2H, H_l), 1.31 (s, 18H, $H_{f,g}$); ^{13}C-NMR (100.5 MHz, $CDCl_3$) δ_C: 149.24, 149.20, 146.87, 146.16, 138.87, 138.82, 137.86, 137.29, 136.01, 129.72, 129.10, 128.95, 128.71, 128.25, 128.20, 128.10, 127.97, 124.71, 109.46, 107.02, 71.19, 34.54, 31.50 (four peaks are missing probably due to overlap); ESI-HRMS: ($[M+H]^+$ $[C_{37}H_{39}O_2]^+$) calc. 515.2944, found 515.2946.

3-(Benzyloxy)-6,7-bis(4-(*tert*-butyl)phenyl)-6'-(3,5-di-*tert*-butylphenyl)-2,2'-dimethoxy-1,1'-binaphthalene 10$^{\mathbf{tBuPh}}$

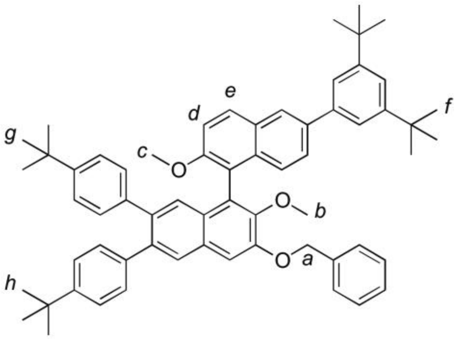

To a mixture of $CuCl_2$ (520 mg, 3.89 mmol) in anhydrous MeOH (25 mL), (±)-1-phenylethylamine (626 μL, 4.86 mmol) was added at 0 °C and the resulting blue-green mixture degassed under argon for 30 min. This was followed by the addition of a solution of **4**$^{\mathbf{tBuPh}}$ (500 mg, 0.97 mmol) and **8** (390 mg, 1.17 mmol) in anhydrous CH_2Cl_2 (25 mL), and the resulting solution was stirred overnight at room temperature. A 1M aqueous solution of HCl (20 mL) was added and the reaction mixture stirred for additional 30 min. This was followed by the addition of CH_2Cl_2 (20 mL) and the mixture was washed with H_2O (2 × 20 mL). The resulting aqueous phase was then extracted with CH_2Cl_2 (3 × 20 mL). The combined organic layers were dried over $MgSO_4$ and evaporated *in vacuo*. The residue was directly subjected to a silica gel column chromatography (eluents: cyclohexane/CH_2Cl_2, 1:1 to CH_2Cl_2) affording an unseparable mixture of the 3 possible products, amongst which desire **9**$^{\mathbf{tBuPh}}$ (870 mg). To a solution of the obtained mixture (845 mg, 1.00 mmol) and K_2CO_3 (2.07 g, 15.00 mmol) in anhydrous acetone (50 mL), MeI (1.25 mL, 20.00 mmol) was added under argon at room temperature. The resulting mixture was refluxed for 2 h and further stirred overnight at room temperature. A 1M aqueous solution of HCl (20 mL) was added to the solution, and the mixture extracted with H_2O (2 × 30 mL). The aqueous layer was washed with CH_2Cl_2 (3 × 30 mL). The combined organic layers were dried over $MgSO_4$ and evaporated *in vacuo*. The residue was purified by silica gel column chromatography (eluents: CH_2Cl_2/cyclohexane, 1:1) affording desired **10**$^{\mathbf{tBuPh}}$ (210 mg, 25% over two steps) as white solids. $C_{63}H_{68}O_3$; MW: 872.52 g/mol; M.p. > 250 °C; IR (cm^{-1}): ν 596.85, 695.93, 711.72, 734.71, 834.73, 873.16, 1027.56, 1048.32, 1093.59, 1141.33, 1201.60, 1249.09, 1361.90, 1461.38, 1476.68, 1590.71, 2960.03; ^1H-NMR (270 MHz, CD_2Cl_2) δ_H: 8.12-8.09 (m, 2H, $H_{d,e}$), 7.85 (s, 1H, Ar*H*), 7.63-7.39 (m, 12H, Ar*H*), 7.32-7.27 (m, 2H, Ar*H*), 7.17-7.08 (m, 5H, Ar*H*), 6.90-6.88 (m, 2H, Ar*H*), 5.38 (s, 2H, H_a), 3.91 (s, 3H, H_c), 3.68 (s, 3H, H_b), 1.41 (s, 18H, H_f), 1.33 (s, 9H, H_g), 1.22 (s, 9H, H_h); ^{13}C-NMR (68 MHz, CD_2Cl_2) δ_C: 155.23, 152.32, 151.70, 149.78,

149.52, 148.54, 140.90, 139.17, 138.83, 137.93, 137.77, 137.41, 133.44, 130.99, 130.31, 129.93, 129.84, 129.64, 129.05, 128.83, 128.66, 128.44, 127.99, 127.12, 127.08, 126.82, 126.27, 125.85, 125.08, 124.89, 122.19, 121.72, 118.93, 114.15, 108.79, 70.89, 60.93, 56.77, 35.31, 34.73, 34.59, 31.71, 31.51, 31.40 (one peak is missing probably due to overlap); MALDI-HRMS: ($[M]^+$ $[C_{63}H_{68}O_3]^+$) calc. 872.5168, found 872.5159.

6,7-Bis(4-(*tert*-butyl)phenyl)-6'-(3,5-di-*tert*-butylphenyl)-2,2'-dimethoxy-[1,1'-binaphthalen]-3-ol 11$^{\mathbf{tBuPh}}$

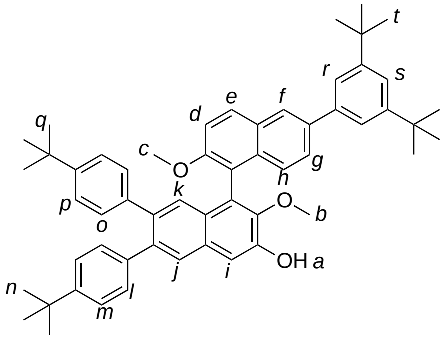

To a suspension of 10% Pd/C (112 mg, 1.06 mmol) in $CHCl_3$ (5 mL) at 0 °C under argon, a solution containing **10$^{\mathbf{tBuPh}}$** (185 mg, 0.21 mmol) in $CHCl_3$ (5 mL) and AcOH (0.5 mL) was added. The solution was degassed with H_2 for 40 min and the resulting dark solution stirred for 24 h at room temperature, under H_2 atmosphere. The solution was filtered and the solvent evaporated under vacuum. The residue was purified by silica gel column chromatography (eluents: CH_2Cl_2/cyclohexane, 1:1 with gradient to CH_2Cl_2) affording **11$^{\mathbf{tBuPh}}$** as a white solid (110 mg, 67%). $C_{56}H_{62}O_3$; MW: 783.09 g/mol; M.p.: 162-165 °C; IR (cm^{-1}): ν 833.99, 874.09, 1016.58, 1041.34, 1087.94, 1224.83, 1250.93, 1268.63, 1362.49, 1416.94, 1462.12, 1479.15, 1591.28, 2904.11, 2961.00; ^{1}H-NMR (270 MHz, CD_2Cl_2) δ_H: 8.17-8.12 (m, 2H, $H_{d,e}$), 7.83 (s, 1H, H_j), 7.65-7.49 (m, 6H, $H_{f,h,i,r,s}$), 7.35-7.28 (m, 3H, $H_{g,o}$), 7.18-7.11 (m, 5H, $H_{k,l,p}$), 6.92-6.89 (m, 2H, H_m), 6.30 (s, 1H, H_a), 3.94 (s, 3H, H_c), 3.49 (s, 3H, H_b), 1.43 (s, 18H, H_t), 1.34 (s, 9H, H_q), 1.23 (s, 9H, H_n); ^{13}C-NMR (68 MHz, CD_2Cl_2) δ_C: 155.46, 151.76, 149.79, 149.50, 148.75, 146.93, 140.78, 139.24, 139.16, 139.00, 138.14, 137.46, 133.33, 131.30, 130.97, 129.93,

129.85, 129.74, 128.67, 128.44, 127.41, 127.02, 126.34, 125.84, 125.08, 124.89, 124.46, 122.19, 121.83, 118.20, 114.08, 109.80, 61.21, 56.80, 35.34, 34.73, 34.60, 31.73, 31.53, 31.43; MALDI-HRMS: ($[M]^+$ $[C_{56}H_{62}O_3]^+$) calc. 782.4699, found 782.4675.

1,2-Bis(4-(*tert*-butyl)phenyl)-8-(3,5-di-*tert*-butylphenyl)-4-(6-(3,5-di-*tert*-butylphenyl)-2-methoxynaphthalen-1-yl)-5-methoxyxantheno[2,1,9,8-klmna]xanthene 15^{tBuPh}

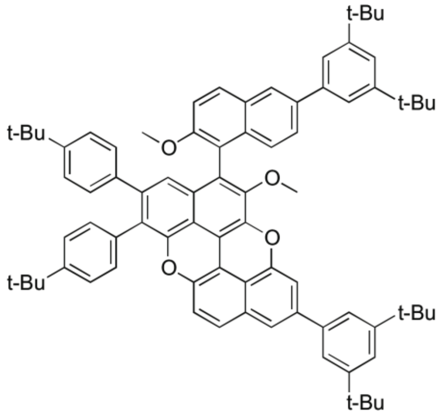

To a mixture of $CuCl_2$ (171 mg, 1.28 mmol) in anhydrous MeOH (15 mL), (±)-1-phenylethylamine (205 μL, 1.60 mmol) was added at 0 °C and the resulting blue-green mixture degassed under argon for 30 min. This was followed by the addition of a solution of **11**^{tBuPh} (250 mg, 0.32 mmol) and **8** (220 mg, 0.64 mmol) in anhydrous CH_2Cl_2 (15 mL), and the resulting solution stirred overnight at room temperature. A 1M aqueous solution of HCl (20 mL) was added and the solution stirred for another 30 min. CH_2Cl_2 (20 mL) was added and the mixture washed with H_2O (3 × 20 mL). The aqueous phase was then extracted with CH_2Cl_2 (3 × 25 mL) and the combined organic layers dried over $MgSO_4$ and evaporated *in vacuo*. The residue was purified by silica gel column chromatography (eluents: CH_2Cl_2/pentane, 1:1 with a gradient to CH_2Cl_2) affording 1**4**^{tBuPh} in a mixture of the 3 possible compounds (250 mg). $C_{80}H_{88}O_4$; MW: 1113.55 g/mol; MALDI-HRMS: ($[M]^+$ $[C_{80}H_{88}O_4]^+$) calc. 1112.6683, found 1112.6677. To the obtained mixture (250 mg, 0.22 mmol), CuI (128 mg, 0.67 mmol), pivalic acid (46 mg, 0.45 mmol) and anhydrous DMSO (5 mL) were added and the resulting mixture heated to 145 °C for 2 h. The resulting crude was filtered, washed with CH_2Cl_2 and

evaporated *in vacuo*. The black residue was purified by silica gel column chromatography (eluents: CH_2Cl_2/cyclohexane, 3:7 with a gradient to CH_2Cl_2) followed by precipitation in MeOH affording **15**$^{\mathbf{tBuPh}}$ as a yellow solid (69 mg, 19% over 2 steps). $C_{80}H_{84}O_4$; MW: 1109.52 g/mol; M.p.: decomposition before reaching melting point; IR (cm^{-1}): ν 711.20, 797.45, 819.04, 833.88, 852.76, 872.88, 1054.21, 1075.99, 1142.20, 1205.62, 1246.77, 1361.96, 1393.01, 1454.70, 1591.61, 2955.07; UV-vis (CH_2Cl_2, r.t.): λ_{max} (ε_{max}, mol^{-1} L cm^{-1}) 227 (87142), 262 (119172), 284 (95153), 344 (12760), 399 (8506), 424 (15820), 452 (19572); ^{1}H-NMR (400 MHz, CD_2Cl_2) δ_H: 8.08-8.06 (m, 2H, Ar*H*), 7.65-7.63 (m, 1H, Ar*H*), 7.53-7.41 (m, 10H, Ar*H*), 7.2-7.24 (m, 2H, Ar*H*), 7.12-7.08 (m, 3H, Ar*H*), 7.00-6.98 (m, 2H, Ar*H*), 6.94-6.92 (m, 1H, Ar*H*), 6.74-7.72 (m, 2H, Ar*H*), 6.41 (s, 1H, Ar*H*), 3.93 (s, 3H, OC*H*3), 3.74 (s, 3H, OC*H*3), 1.40-1.39 (s, 36H, CC*H*3), 1.30 (s, 9H, CC*H*3), 1.15 (s, 9H, CC*H*3); ^{13}C-NMR (100.5 MHz, CD_2Cl_2) δ_C: 155.19, 152.92, 151.80, 151.70, 149.90, 149.60, 147.08, 144.72, 142.79, 142.16, 140.77, 140.47, 139.38, 138.84, 137.96, 133.05, 132.99, 131.77, 131.21, 130.51, 129.74, 129.58, 127.23, 127.14, 126.34, 125.54, 124.78, 124.57, 122.28, 122.14, 121.90, 121.75, 120.57, 119.72, 118.91, 118.31, 117.96, 117.61, 114.18, 113.71, 111.56, 109.37, 61.39, 56.82, 35.31, 35.30, 34.73, 34.50, 31.66, 31.44, 31.29 (four aromatic peaks and one of the C*C*H3 carbon are missing due to overlap); MALDI-HRMS: ($[M]^+$ $[C_{80}H_{84}O_4]^+$) calc. 1108.6670, found 1108.6400.

1,2-Bis(4-(*tert*-butyl)phenyl)-8-(3,5-di-*tert*-butylphenyl)-4-(6-(3,5-di-*tert*-butylphenyl)-2-hydroxynaphthalen-1-yl)xantheno[2,1,9,8-klmna]xanthen-5-ol 16$^{\mathbf{tBuPh}}$

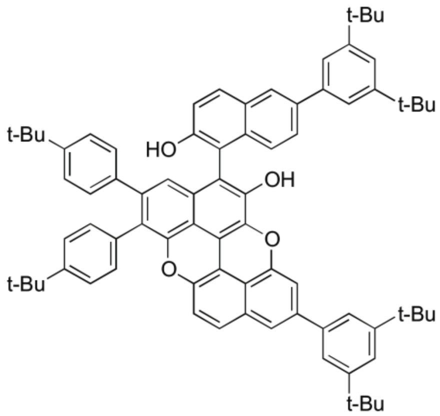

To a solution of **15^tBuPh^** (40 mg, 0.036 mmol) in anhydrous CH_2Cl_2 (10 mL), BBr_3 (110 µL, 0.11 mmol, 1M in CH_2Cl_2) was added at 0 °C under argon. The resulting mixture was stirred overnight at room temperature. H_2O (10 mL) was added and the solution stirred 30 min. The mixture was washed with H_2O (2 × 15 mL) and the aqueous layer extracted with CH_2Cl_2 (2 × 20 mL). The combined organic layers were dried over $MgSO_4$ and evaporated *in vacuo*. The residue was purified by silica gel column chromatography (eluents: CH_2Cl_2/cyclohexane, 1:1) affording **16^{tBuPh}** as a yellow solid (25 mg, 64%). $C_{78}H_{80}O_4$; MW: 1081.47 g/mol; M.p.: decomposition before reaching melting point; IR (cm^{-1}): ν 562.90, 711.30, 736.69, 801.29, 861.19, 1019.18, 1096.43, 1260.75, 1362.73, 1392.92, 1462.02, 1509.58, 1593.95, 2867.54, 2960.65; ^{1}H-NMR (400 MHz, CD_2Cl_2) δ_H: 8.08 (d, *J* = 1.60 Hz, 1H, Ar*H*), 8.02 (d, *J* = 8.93 Hz, 1H, Ar*H*), 7.68 (dd, *J* = 1.83 Hz, 8.70 Hz, 1H, Ar*H*), 7.52-7.45 (m, 9H, Ar*H*), 7.38 (d, *J* = 8.93 Hz, 1H, Ar*H*), 7.27-7.24 (m, 2H, Ar*H*), 7.15-6.98 (m, 6H, Ar*H*), 6.76-6.73 (m, 2H, Ar*H*), 6.49 (bs, 1H, Ar*H*), 5.55 (s, 1H, O*H*), 5.37 (s, 1H, O*H*), 1.39 (s, 36H, CCH_3), 1.30 (s, 9H, CCH_3), 1.15 (s, 9H, CCH_3); MALDI-HRMS: ([M]$^+$ [$C_{78}H_{80}O_4$]$^+$) calc. 1080.6057, found 1080.6096.

7,8-bis(4-(*tert*-butyl)phenyl)-2,13-bis(3,5-di-*tert*-butylphenyl)-tetraoxapentapheno-pentaphene 17^{tBuPh}

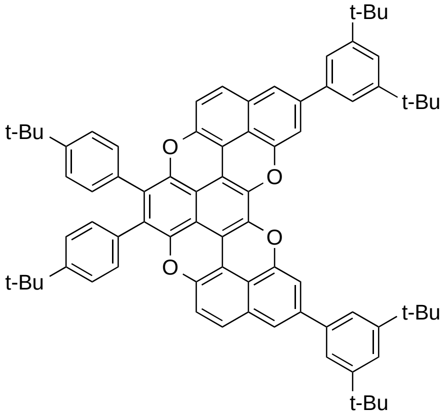

To **16^tBuPh^** (21 mg, 0.019 mmol), CuI (11 mg, 0.058 mmol) and pivalic acid (4 mg, 0.039 mmol), anhydrous DMSO (3 mL) was added and the resulting mixture heated to 145 °C for 1h under open-air condition. The solution was filtered, washed with CH_2Cl_2 and evaporated under vacuum. The residue was purified by silica gel column chromatography (eluents: from CH_2Cl_2/cyclohexane, 1:1 to CH_2Cl_2/EtOAc, 1:1) affording **17^{tBuPh}** as a red solid (10 mg, 49%). $C_{78}H_{76}O_4$; MW: 1077.44 g/mol; IR (cm^{-1}): ν 816.47, 836.76, 857.16, 1107.35, 1144.83, 1194.21, 1246.99, 1310.37, 1362.33, 1392.85, 1461.27, 1508.02, 1593.82, 2865.90, 2959.38; UV-vis (CH_2Cl_2, r.t.): λ_{max} (ε_{max}, mol^{-1} L cm^{-1}) 268 (76557), 509 (5800), 552 (5361); MALDI-HRMS: ($[M]^+$ $C_{78}H_{76}O_4^+$) calc. 1076.5744, found 1076.5760.

6',6'',7',7''-Tetrakis(4-(*tert*-butyl)phenyl)-6,6'''-bis(3,5-di-*tert*-butylphenyl)-2,2',2''',3''-tetramethoxy-[1,1':4',1'':4'',1'''-quaternaphthalene]-2'',3'-diol 18^{tBuPh}

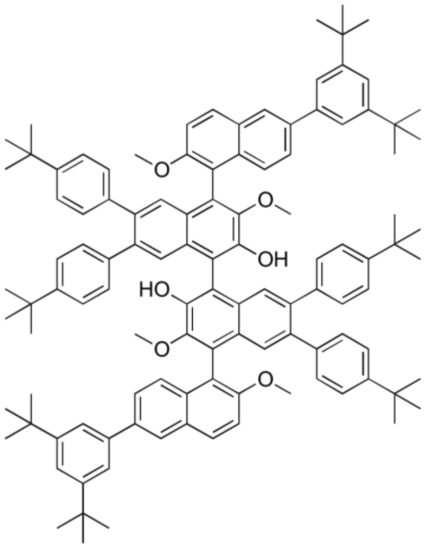

To a mixture of $CuCl_2$ (96 mg, 0.72 mmol) in anhydrous MeOH (15 mL), (±)-1-phenylethylamine (115 μL, 0.89 mmol) was added at 0 °C and the resulting blue-green mixture degassed under argon for 30 min. This was followed by the addition of a solution of **11^{tBuPh}** (280 mg, 0.36 mmol) in anhydrous CH_2Cl_2 (15 mL), and the resulting solution was stirred overnight at room temperature. A 1M aqueous solution of HCl (20 mL) was then added and the solution stirred for additional 30 min. CH_2Cl_2 (20 mL) was added and the mixture washed with H_2O (3 × 20 mL). The aqueous phase was extracted with CH_2Cl_2 (3 × 25 mL). The combined organic layers were dried over $MgSO_4$ and evaporated *in vacuo*. The residue was purified by silica gel column chromatography (eluents: CH_2Cl_2/pentane, 1:1 with a gradient to CH_2Cl_2) affording the mixture of the 3 different diastereoisomers **18^{tBuPh}** (207 mg, 74%). $C_{112}H_{122}O_6$; MW: 1564.16 g/mol; M.p. 208-212 °C; IR (cm^{-1}): ν 543.11, 562.95, 601.15, 711.31, 736.69, 799.40, 873.81, 968.54, 1018.02, 1087.80, 1258.88, 1362.07, 1394.25, 1461.43, 1591.18, 1732.20, 2960.76; ^{1}H-NMR (270 MHz, CD_2Cl_2) δ_H: 8.16 (d, *J* = 9.16 Hz, 2H, Ar*H*), 8.11 (d, *J* = 1.60 Hz, 2H, Ar*H*), 7.61-7.53 (m, 10H, Ar*H*), 7.47-7.43 (m, 4H, Ar*H*), 7.23 (s, 2H, Ar*H*), 7.15-7.13 (m, 4H, Ar*H*), 7.09-7.07 (m, 4H, Ar*H*), 6.99-6.97 (m, 4H, Ar*H*), 6.88-6.86 (m, 4H, Ar*H*), 6.43 (s, 2H, O*H*), 4.02 (s, 6H, OCH_3), 3.59 (s, 6H, OCH_3), 1.40 (s, 36H, CCH_3), 1.20-1.19 (m, 36H, CCH_3); ^{13}C-NMR (100.5 MHz, CD_2Cl_2) δ_C: 155.50, 151.70, 149.65, 149.44, 146.97, 146.76, 140.81, 139.28, 139.17, 139.14, 138.14, 137.71, 133.33, 130.98, 130.57, 129.90, 129.83, 129.71, 128.57, 127.53, 127.35, 126.88, 126.43, 125.96, 124.89, 124.84, 124.72, 122.13, 121.79, 118.18, 115.31, 113.92, 61.30, 56.75, 35.31, 34.61, 34.57, 31.70, 31.49, 31.38; MALDI-HRMS: ($[M]^+$ $[C_{112}H_{122}O_6]^+$) calc. 1562.9241, found 1562.9214.

1,2,7,8-Tetrakis(4-(*tert*-butyl)phenyl)-4,10-bis(6-(3,5-di-*tert*-butylphenyl)-2-methoxynaphthalen-1-yl)-5,11-dimethoxyxantheno[2,1,9,8-*klmna*]xanthene 19^{tBuPh}

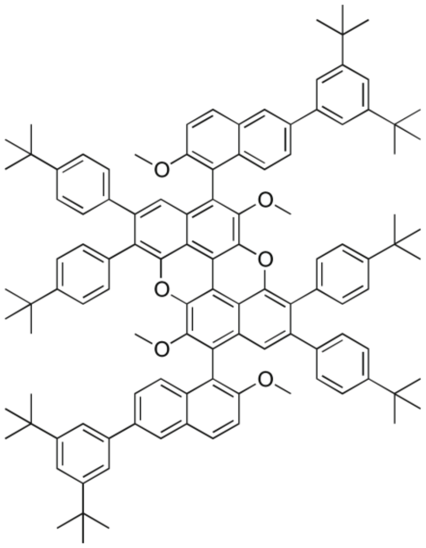

To **18^tBuPh^** (95 mg, 0.060 mmol), CuI (35 mg, 0.18 mmol) and pivalic acid (13 mg, 0.12 mmol), anhydrous DMSO (2 mL) was added and the resulting mixture heated at 145 °C for 1 h. The resulting crude was filtered, washed with CH_2Cl_2 and evaporated *in vacuo*. The black residue was purified by silica gel column chromatography (eluents: CH_2Cl_2/cyclohexane, 1:1 with a gradient to CH_2Cl_2) affording **19^{tBuPh}** as a yellow solid (60 mg, 64%). $C_{112}H_{118}O_6$; MW: 1560.13 g/mol; M.p. > 250 °C; IR (cm^{-1}): ν 711.43, 736.58, 799.75, 833.82, 873.25, 1020.55, 1077.33, 1111.76, 1250.20, 1308.31, 1343.38, 1362.67, 1392.94, 1461.40, 1590.21, 2903.46, 2959.61; UV-vis (CH_2Cl_2, r.t.): λ_{max} ($\varepsilon_{max,}$ mol^{-1} L cm^{-1}) 262 (33816), 292 (19346), 350 (3394), 402 (1875), 426 (3414), 455 (3927);

(±) *Trans*-like diastereoisomer

^{1}H-NMR (400 MHz, CD_2Cl_2) δ_H: 8.04-8.02 (m, 4H, Ar*H*), 7.62 (dd, *J* = 1.83 Hz, 8.70 Hz, 2H, Ar*H*), 7.52-7.42 (m, 10H, Ar*H*), 7.26-7.02 (m, 12H, Ar*H*), 6.86-6.84 (m, 4H, Ar*H*), 6.39 (s, 2H, Ar*H*), 3.92 (s, 6H, OCH_3), 3.25 (s, 6H, OCH_3), 1.38 (s, 36H, CCH_3), 1.21 (s, 18H, CCH_3), 1.14 (s, 18H, CCH_3); ^{13}C-NMR (100.5 MHz, CD_2Cl_2) δ_C: 155.01, 151.65, 150.01, 149.64, 146.85, 142.29, 140.72, 139.22, 138.67, 137.82, 133.21, 132.97, 130.98, 130.30, 129.58, 127.09, 126.25, 125.45, 124.85, 124.68, 124.43, 122.37, 122.08, 121.70, 119.66, 117.91, 116.98, 114.00, 113.33, 60.84, 56.82, 35.26, 34.65, 34.49, 31.62, 31.34, 31.24 (three peaks are missing probably due to overlap); MALDI-HRMS: ($[M]^+$ $[C_{112}H_{118}O_6]^+$) calc. 1558.8928, found 1558.8900.

(±) *Cis*-like diastereoisomer

^{1}H-NMR (400 MHz, CD_2Cl_2) δ_H: 8.04-8.02 (m, 4H, Ar*H*), 7.65-7.62 (m, 2H, Ar*H*), 7.52-7.43 (m, 10H, Ar*H*), 7.26-7.02 (m, 12H, Ar*H*), 6.86-6.84 (m, 4H, Ar*H*), 6.40 (s, 2H, Ar*H*), 3.92 (s, 6H, OC*H3*), 3.26 (s, 6H, OC*H3*), 1.39 (s, 36H, CC*H3*), 1.21 (s, 18H, CC*H3*), 1.15 (s, 18H, CC*H3*); ^{13}C-NMR (100.5 MHz, CD_2Cl_2) δ_C: 155.12, 151.71, 150.07, 149.71, 146.96, 142.39, 140.80, 139.30, 138.75, 137.91, 133.29, 133.06, 131.05, 130.35, 129.71, 129.65, 127.12, 126.32, 125.52, 124.90, 124.72, 124.49, 122.44, 122.13, 121.75, 119.72, 118.07, 117.05, 114.15, 113.38, 60.86, 56.80, 35.31, 34.70, 34.52, 31.68, 31.41, 31.30 (two peaks are missing probably due to overlap); MALDI-HRMS: ($[M]^+$ $[C_{112}H_{118}O_6]^+$) calc. 1558.8928, found 1558.8955.

1,2,7,8-Tetrakis(4-(*tert*-butyl)phenyl)-4,10-bis(6-(3,5-di-*tert*-butylphenyl)-2-hydroxynaphthalen-1-yl)xantheno[2,1,9,8-*klmna*]xanthene-5,11-diol 20^{tBuPh}

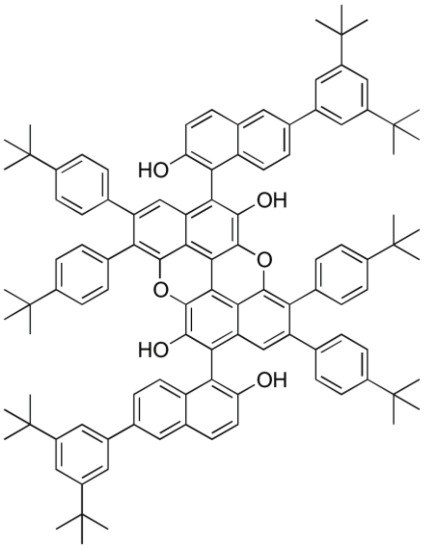

To a solution of **19^{tBuPh}** (45 mg, 0.029 mmol) in anhydrous CH_2Cl_2 (10 mL), BBr_3 (86 μL, 0.086 mmol, 1M in CH_2Cl_2) was added at 0 °C under argon. The resulting mixture was stirred overnight at room

temperature. H_2O (10 mL) was then added and the solution stirred for 30 min. The mixture was washed with H_2O (2 × 10 mL) and the aqueous layer extracted with CH_2Cl_2 (2 × 10 mL). The combined organic layers were dried over $MgSO_4$ and evaporated *in vacuo*. The residue was purified by silica gel column chromatography (eluents: CH_2Cl_2/cyclohexane, 1:1) affording **20^tBuPh^** as a yellow solid (37 mg, 85%). $C_{108}H_{110}O_6$; MW: 1504.03 g/mol; M.p. > 250 °C; IR (cm^{-1}): ν 509.09, 557.99, 711.54, 806.72, 822.33, 839.03, 872.67, 1046.82, 1139.69, 1179.54, 1213.83, 1245.98, 1270.20, 1360.99, 1402.23, 1419.70, 1458.37, 1477.33, 1594.60, 2960.82, 3303.10, 3500.40; UV-vis (CH_2Cl_2, r.t.): λ_{max} (ε_{max}, mol^{-1} L cm^{-1}) 260 (97252), 296 (60241), 340 (10045), 400 (5572), 425 (9335), 454 (10112); ^{1}H-NMR (400 MHz, THF-*d8*) δ_H: 8.13-8.00 (m, 4H, Ar*H*), 7.91 (d, *J* = 8.93 Hz, 2H, Ar*H*), 7.58-7.51 (m, 6H, Ar*H*), 7.44-7.42 (m, 4H, Ar*H*), 7.27 (d, *J* = 8.93 Hz, 2H, Ar*H*), 7.15-7.10 (m, 8H, Ar*H*), 7.00 (d, *J* = 8.47 Hz, 4H, Ar*H*), 6.78 (d, *J* = 8.47 Hz, 4H, Ar*H*), 6.52 (s, 2H, O*H*), 5.53 (s, 2H, O*H*), 1.38 (s, 36H, CCH_3), 1.20 (s, 18H, CCH_3), 1.15 (s, 18H, CCH_3); ^{13}C-NMR (100.5 MHz, THF-*d8*) δ_C: 154.63, 151.49, 149.93, 149.30, 149.21, 145.63, 142.80, 141.60, 139.82, 137.60, 136.89, 134.00, 133.54, 132.09, 131.90, 130.83, 130.16, 130.02, 126.94, 126.56, 125.52, 124.70, 124.48, 122.20, 121.44, 120.96, 119.75, 119.67, 116.12, 115.79, 113.30, 113.13, 35.39, 34.75, 34.64, 31.69, 31.47, 31.36; MALDI-HRMS: ($[M]^+$ $[C_{108}H_{110}O_6]^+$) calc. 1502.8302, found 1502.8296

6',6'',7',7''-Tetrakis(4-(*tert*-butyl)phenyl)-6,6'''-bis(3,5-di-*tert*-butylphenyl)-2,2',2''',3''-tetramethoxy-[1,1':4',1'':4'',1'''-quaternaphthalene]-2'',3'-diol 21^tBuPh^

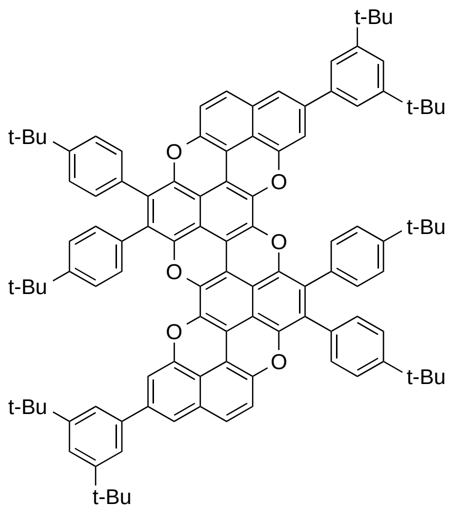

To **20^{tBuPh}** (40 mg, 0.027 mmol), CuI (30 mg, 0.16 mmol) and pivalic acid (12 mg, 0.11 mmol), anhydrous DMSO (3 mL) was added and the resulting mixture heated to 145 °C for 1h under open-air condition. The solution was filtered, washed with CH_2Cl_2 and evaporated under vacuum. The residue was purified by silica gel column chromatography (eluents: from CH_2Cl_2/cyclohexane, 1:1 to CH_2Cl_2/EtOAc, 1:1) affording **21^{tBuPh}** as a green-dark solid (12 mg, 29%). $C_{108}H_{102}O_6$; MW: 1495.96 g/mol; IR (cm^{-1}): ν 563.57, 681.71, 711.05, 800.35, 837.92, 873.78, 1018.51, 1092.03, 1201.29, 1260.21, 1310.52, 1362.28, 1392.74, 1461.27, 1507.21, 1593.50, 1737.84, 2865.49, 2960.91; UV-vis (CH_2Cl_2, r.t.): λ_{max} (ε_{max}, mol^{-1} L cm^{-1}) 264 (94311), 437 (15124), 557 (3107), 621 (2101); MALDI-HRMS: ([M]$^+$ $C_{108}H_{102}O_6^+$) calc. 1494.7556, found 1494.7526.

1.7. NMR Analysis

2-(3,5-Di-*tert*-butylphenyl)-6-methoxynaphthalene **7**

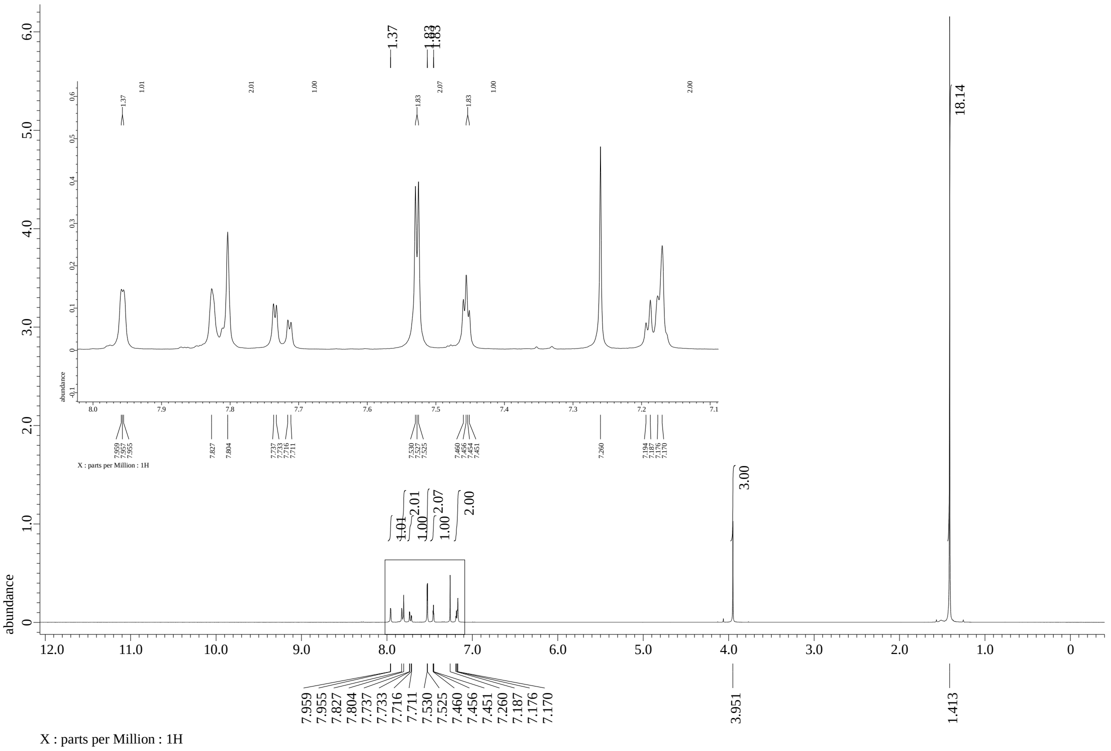

Figure A 1: ^{1}H-NMR (400 MHz, $CDCl_3$)

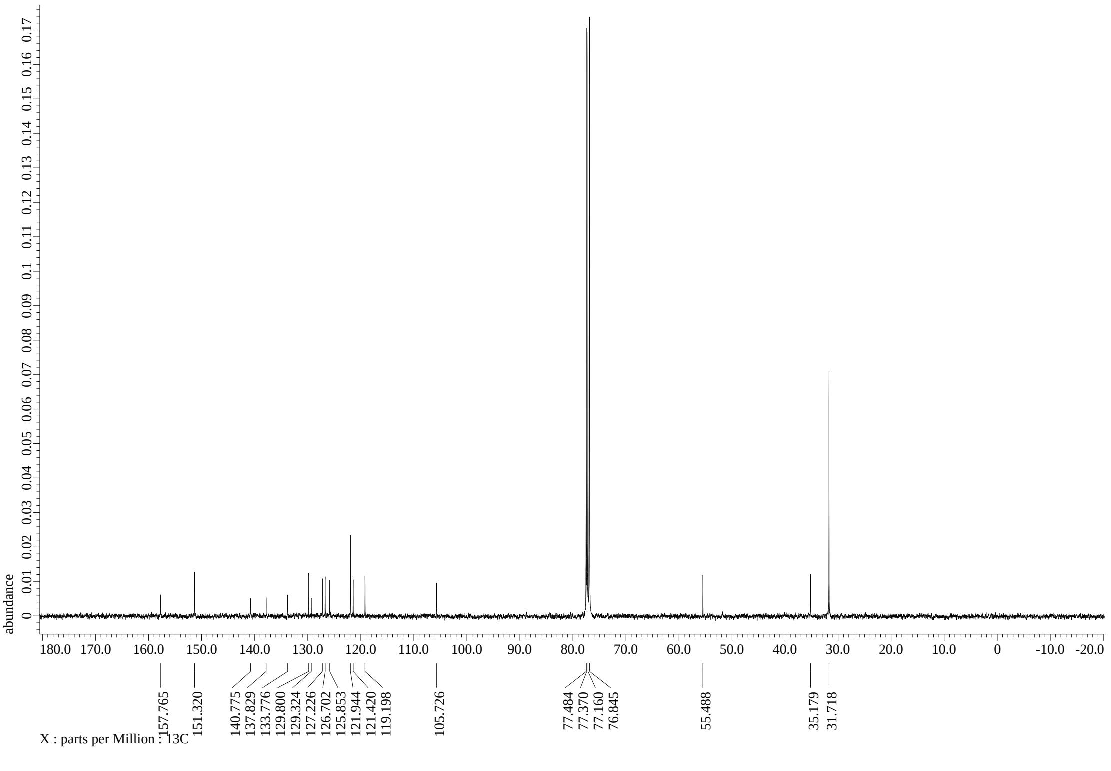

Figure A 2: ^{13}C-NMR (100.5 MHz, $CDCl_3$)

6-(3,5-Di-*tert*-butylphenyl)naphthalen-2-ol **8**

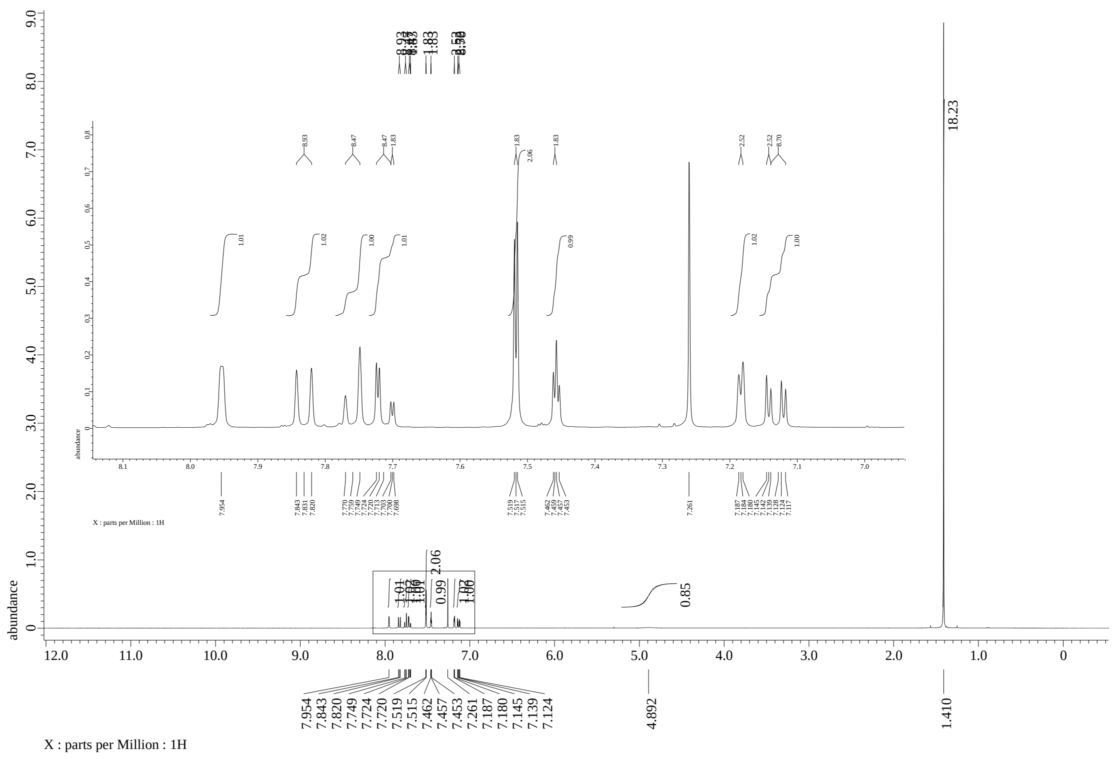

Figure A 3: ^{1}H-NMR (400 MHz, $CDCl_3$)

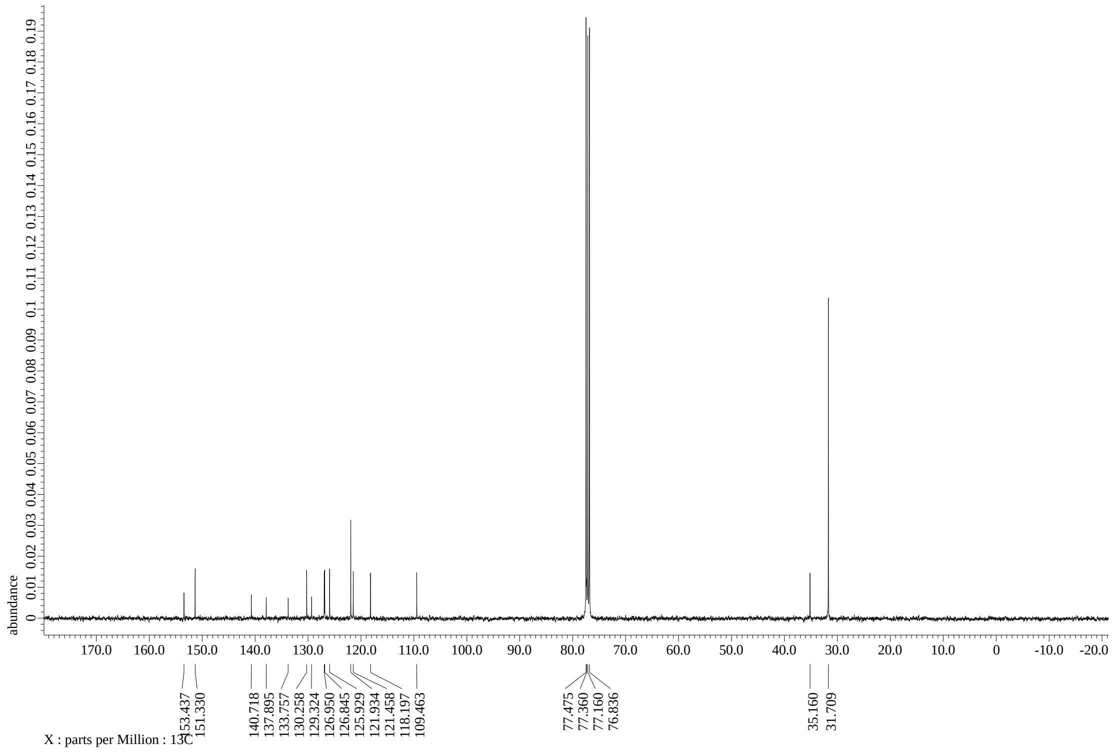

Figure A 4: ^{13}C-NMR (100.5 MHz, $CDCl_3$)

6,6'-Bis(3,5-di-*tert*-butylphenyl)-[1,1'-binaphthalene]-2,2'-diol **12**

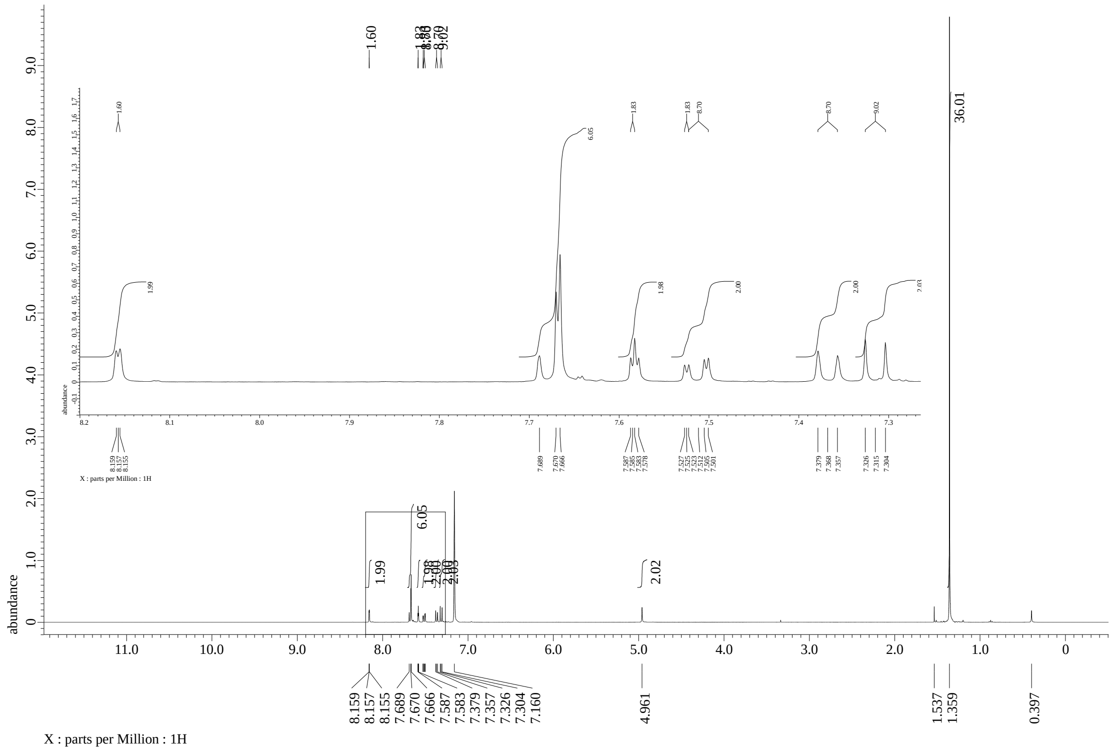

Figure A 5: ^{1}H-NMR (400 MHz, C_6D_6)

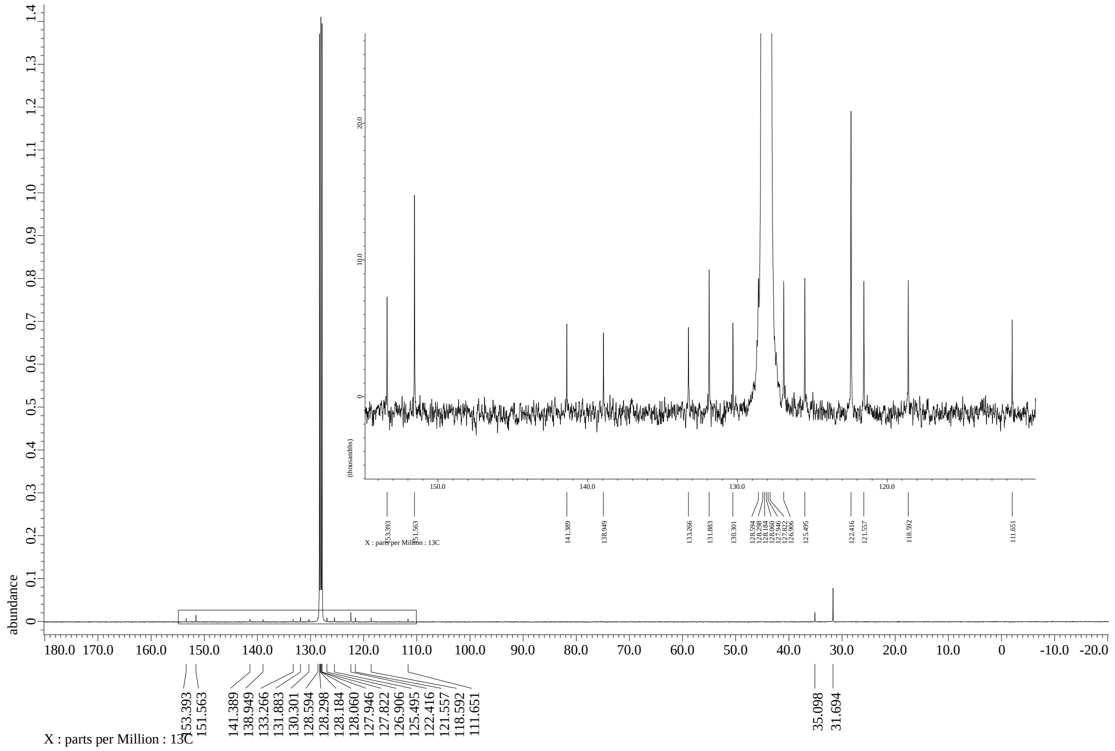

Figure A 6: ^{13}C-NMR (100.5 MHz, C_6D_6)

2,8-Bis(3,5-di-*tert*-butylphenyl)xantheno[2,1,9,8-*klmna*]xanthene **13**

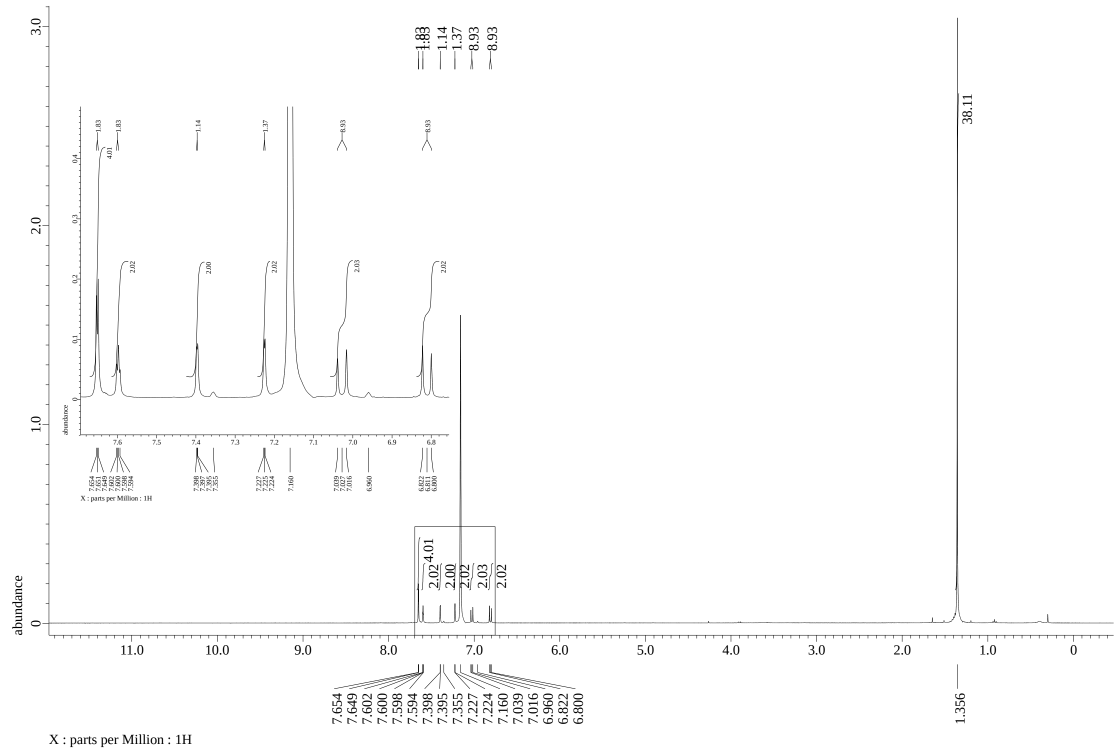

Figure A 7: ^{1}H-NMR (400 MHz, C_6D_6)

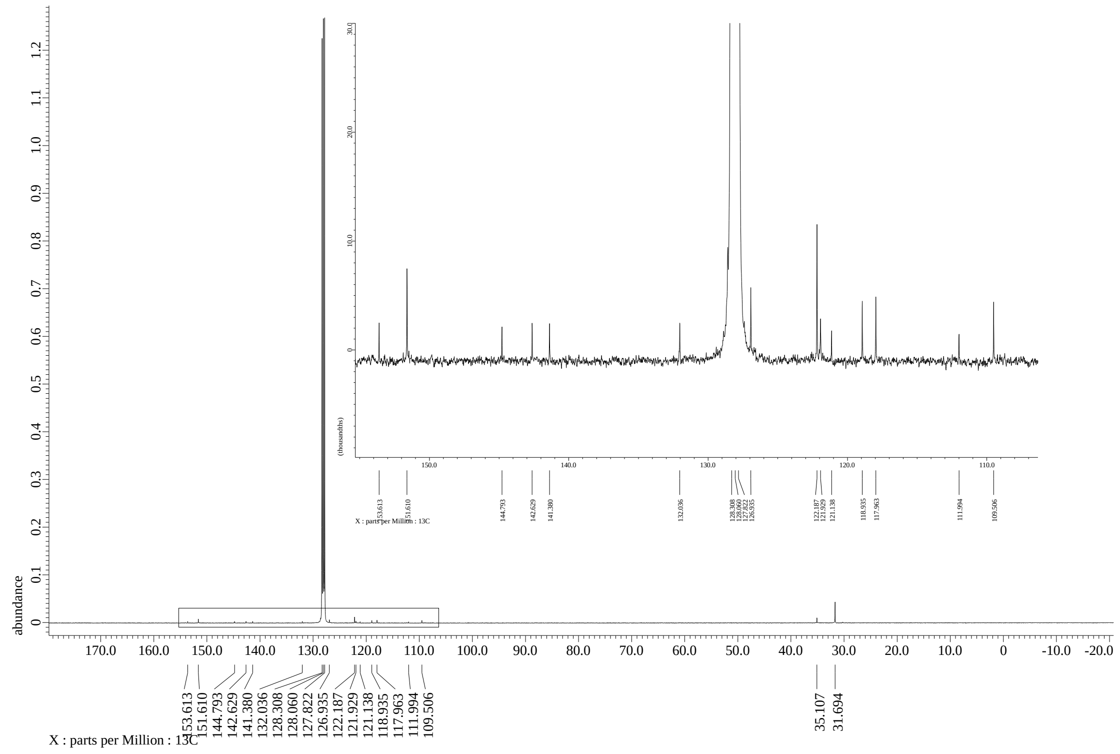

Figure A 8: ^{13}C-NMR (100.5 MHz, C_6D_6)

3-(Benzyloxy)-6'-(3,5-di-*tert*-butylphenyl)-2,2'-dimethoxy-1,1'-binaphthalene **10^H**

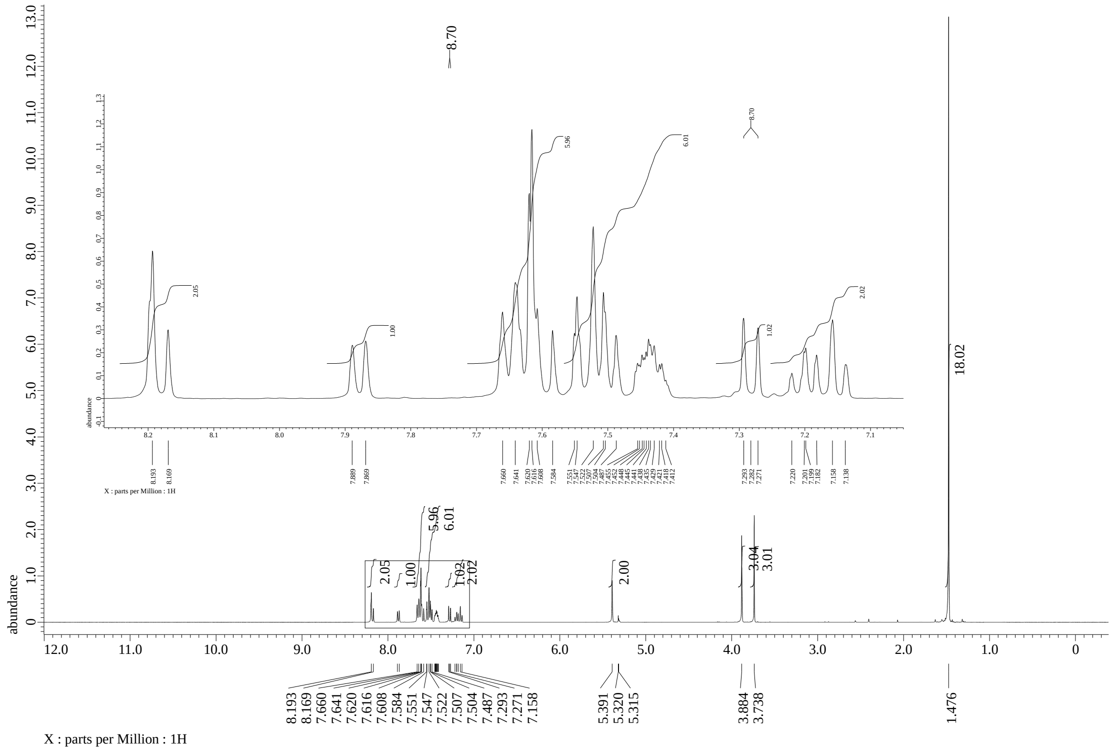

Figure A 9: ^{1}H-NMR (400 MHz, CD_2Cl_2)

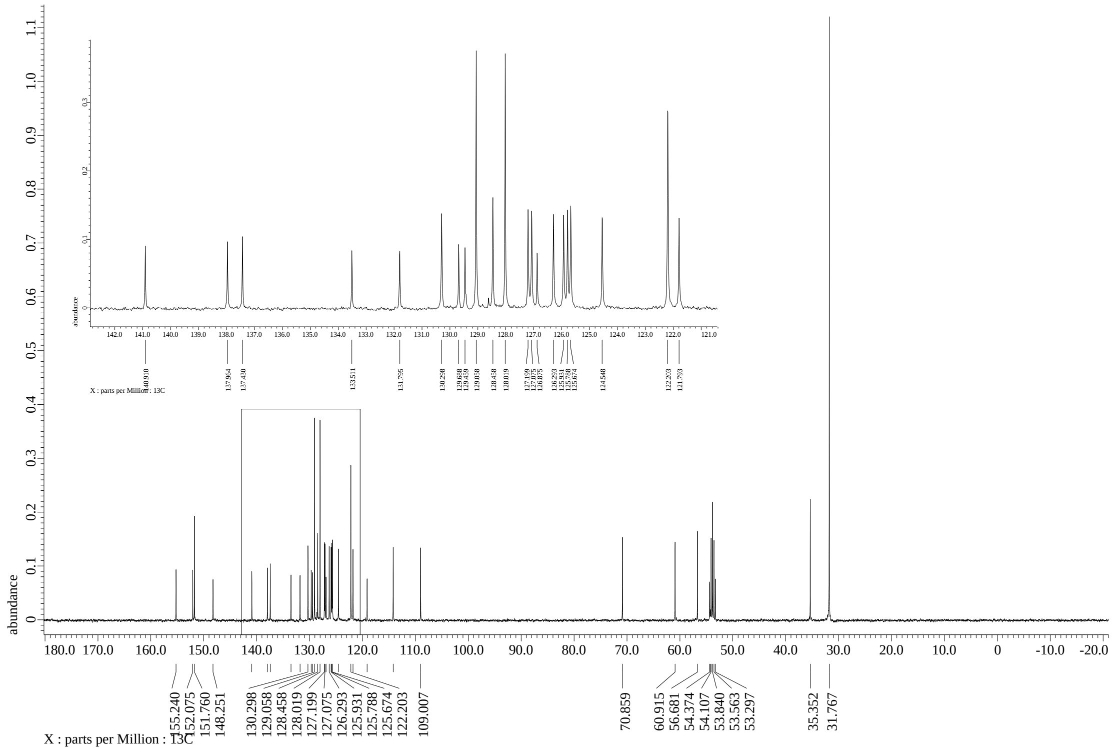

Figure A 10: ^{13}C-NMR (100.5 MHz, CD_2Cl_2)

6'-(3,5-Di-*tert*-butylphenyl)-2,2'-dimethoxy-[1,1'-binaphthalen]-3-ol **11^H^**

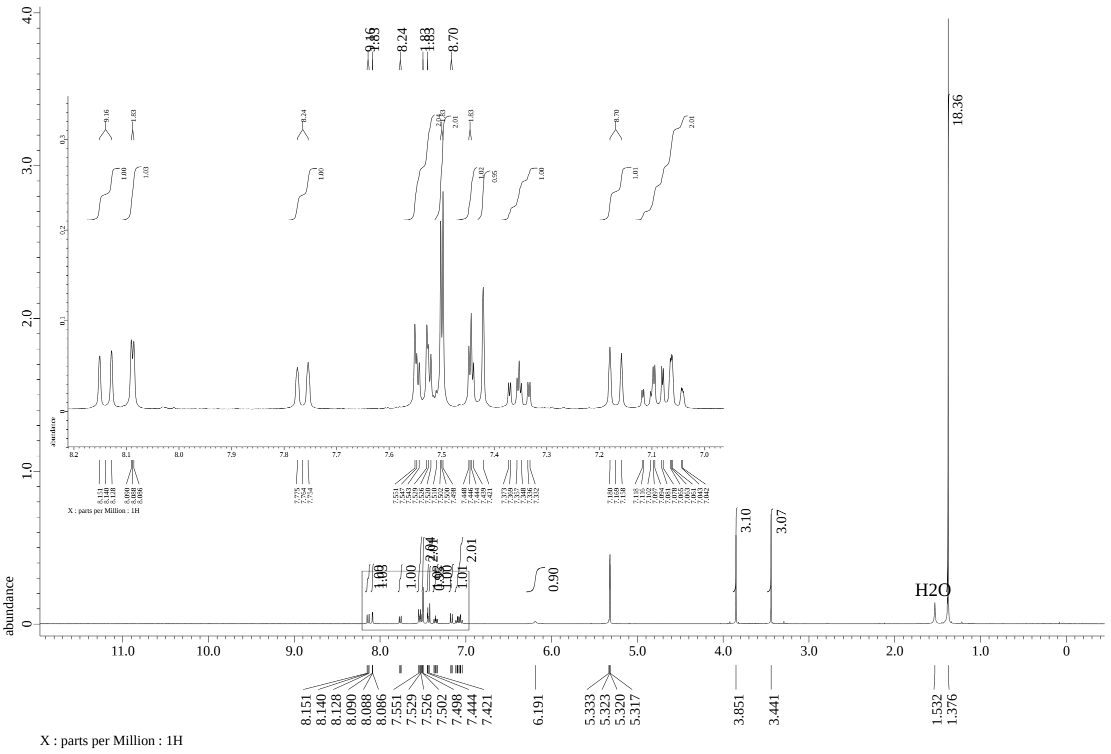

Figure A 11: ^{1}H-NMR (400 MHz, CD_2Cl_2)

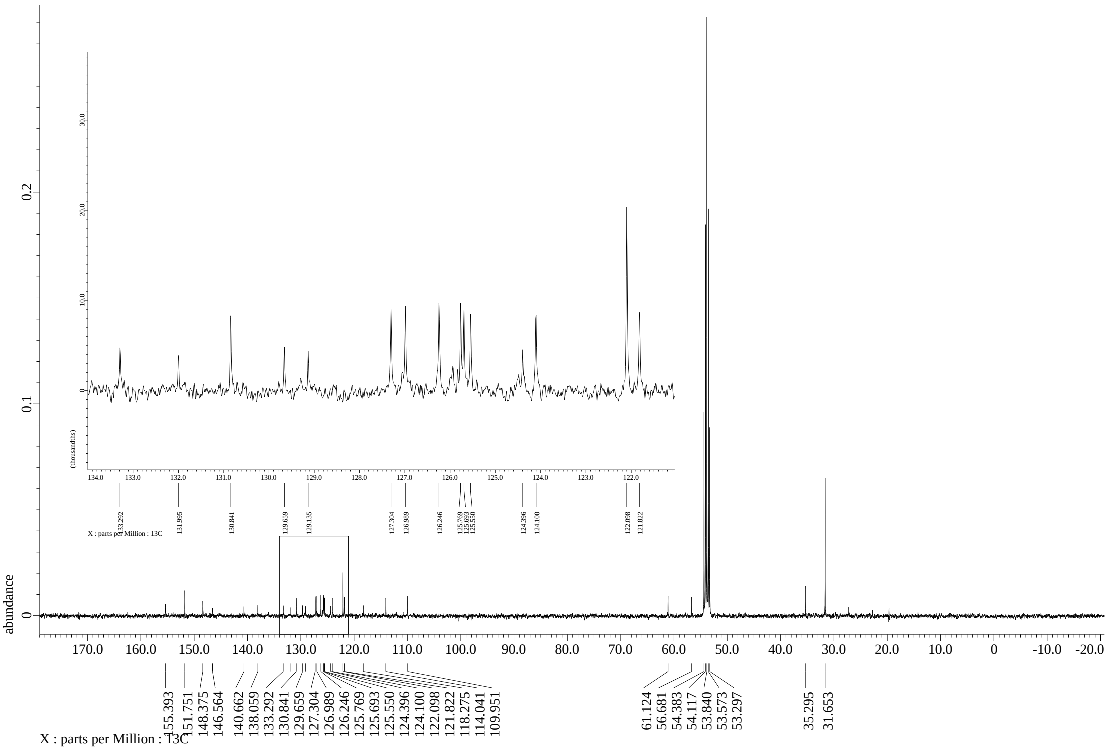

Figure A 12: ^{13}C-NMR (100.5 MHz, CD_2Cl_2)

2-(3,5-Di-*tert*-butylphenyl)-10-(6-(3,5-di-*tert*-butylphenyl)-2-methoxynaphthalen-1-yl)-11-methoxyxantheno[2,1,9,8-*klmna*]xanthene **15H**

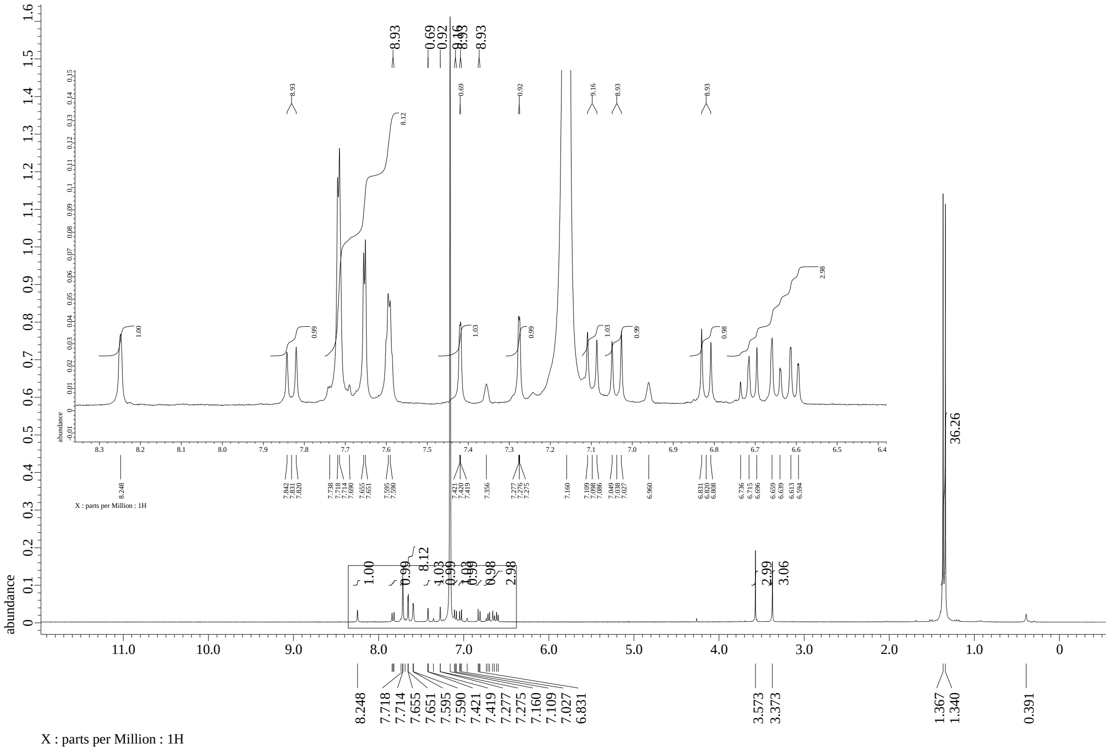

Figure A 13: ^{1}H-NMR (400 MHz, C_6D_6)

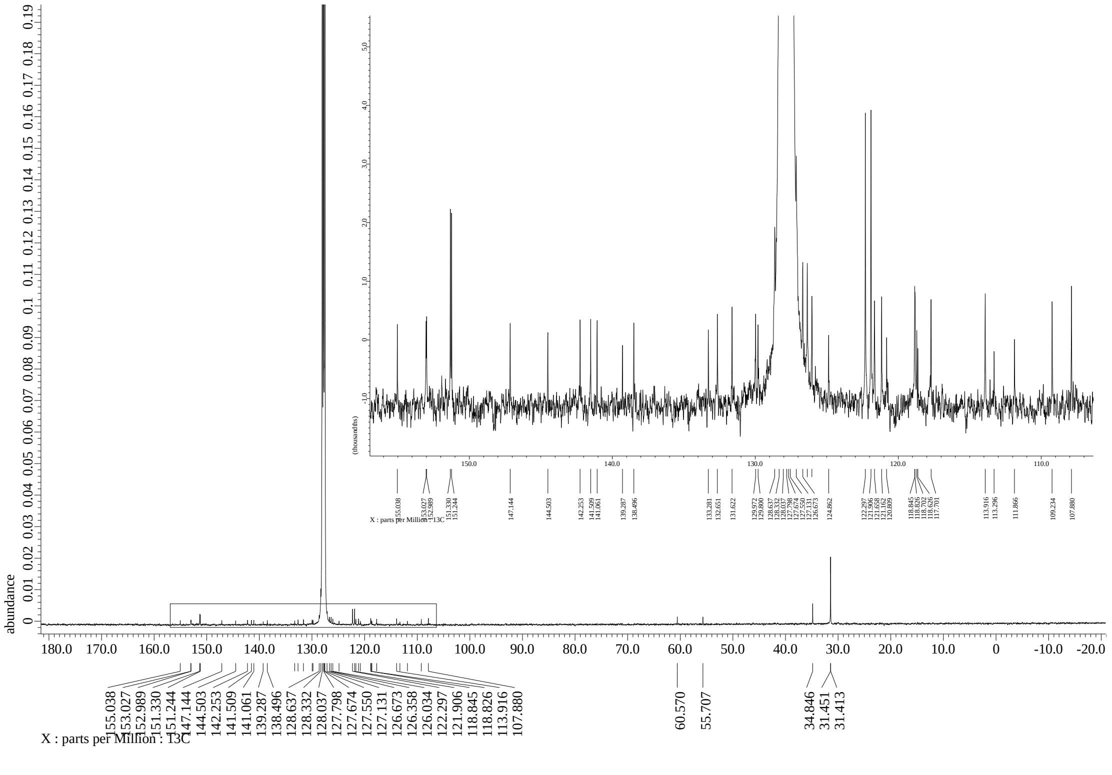

Figure A 14: ^{13}C-NMR (100.5 MHz, C_6D_6)

8-(3,5-Di-*tert*-butylphenyl)-4-(6-(3,5-di-*tert*-butylphenyl)-2-hydroxynaphthalen-1-yl)xantheno[2,1,9,8-*klmna*]xanthen-5-ol **16[H]**

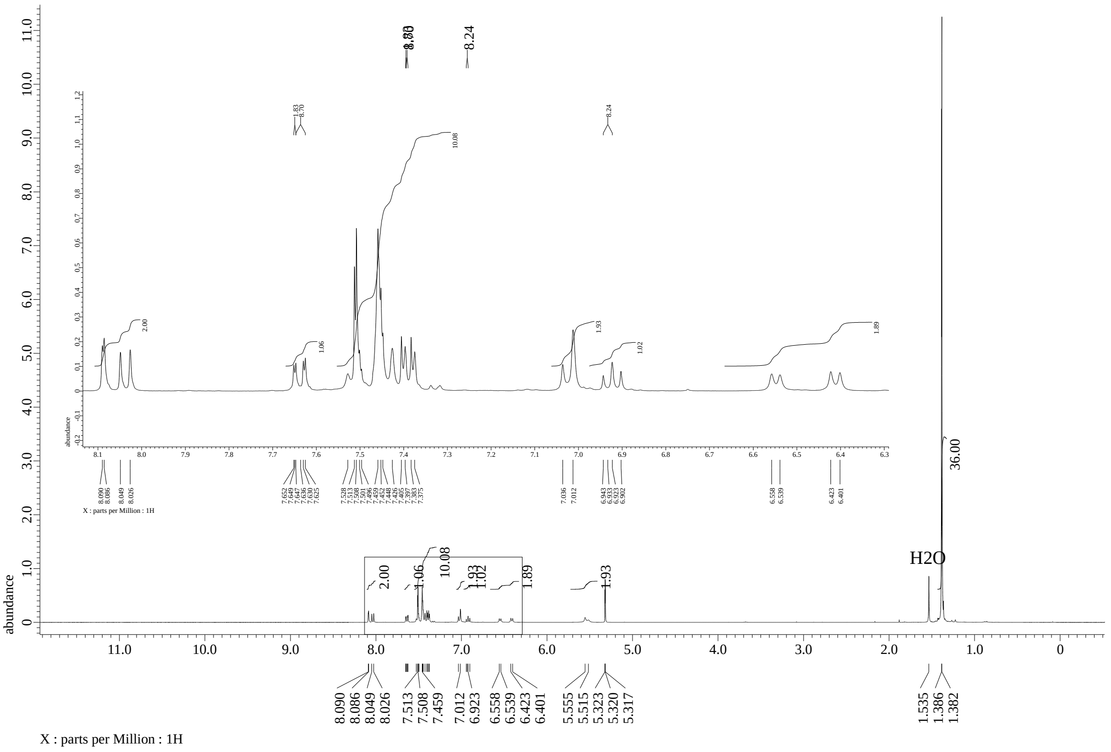

Figure A 15: ^{1}H-NMR (400 MHz, CD_2Cl_2)

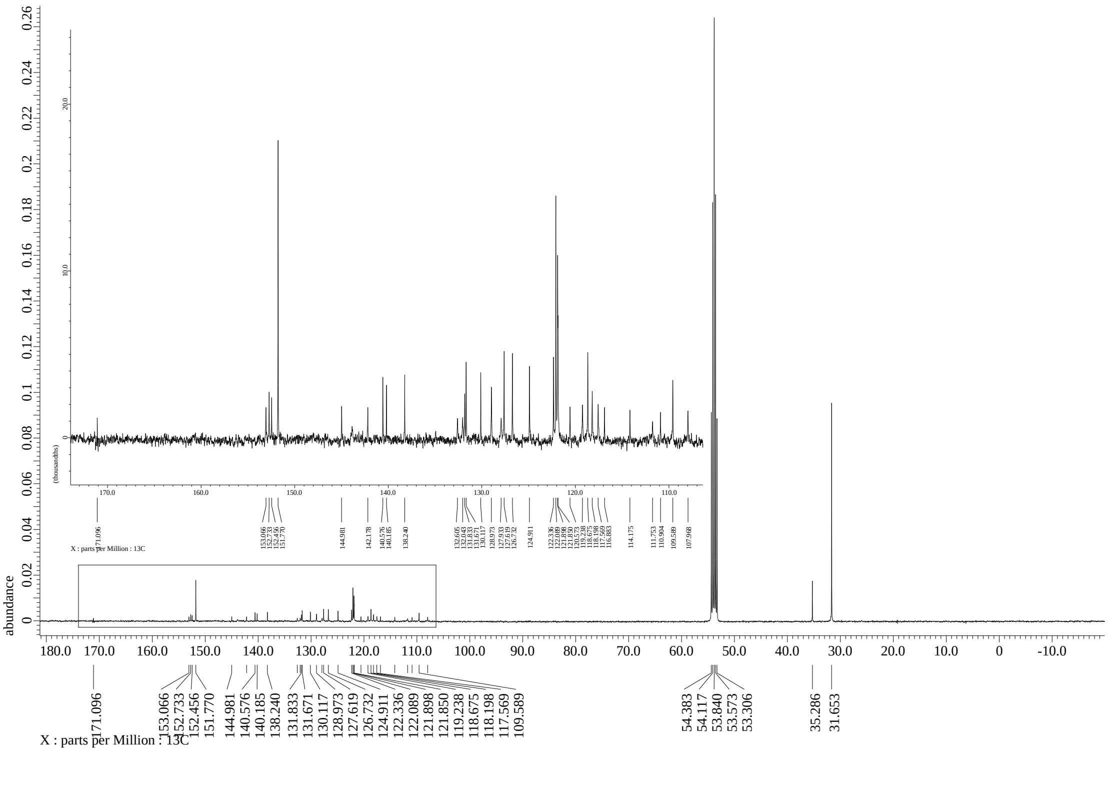

Figure A 16: ^{13}C-NMR (100.5 MHz, CD_2Cl_2)

6,6'''-Bis(3,5-di-*tert*-butylphenyl)-2,2',2''',3''-tetramethoxy-[1,1':4',1'':4'',1'''-quaternaphthalene]-2'',3'-diol **18^H^**

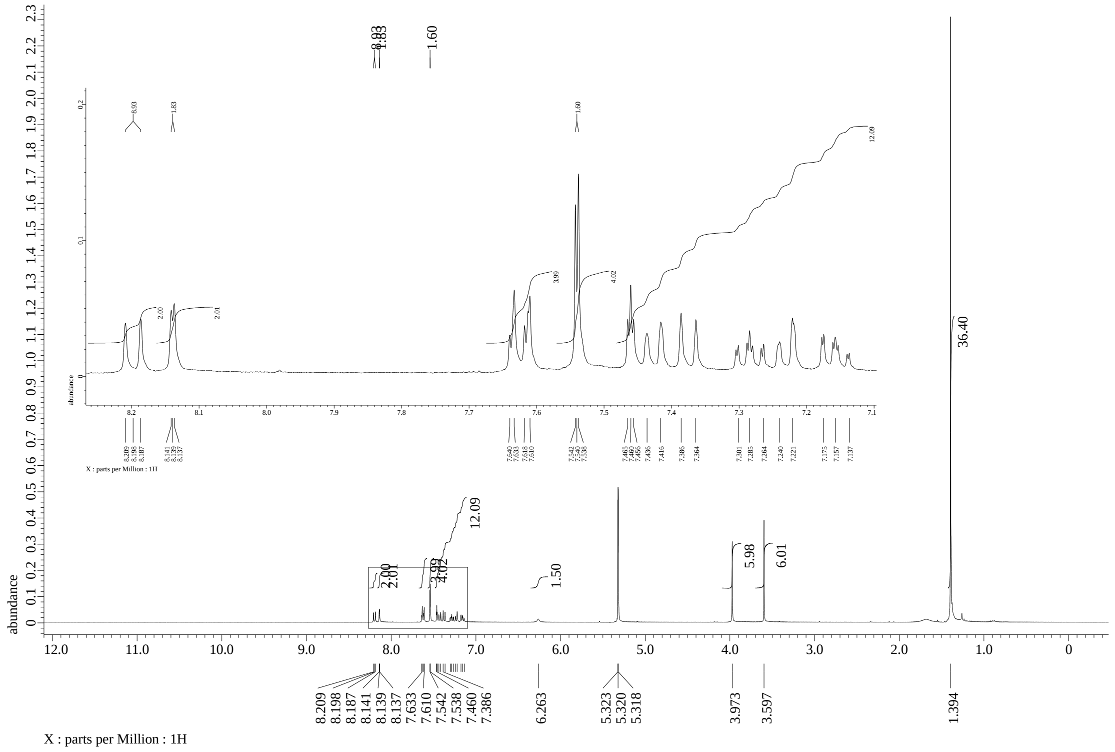

Figure A 17: ^{1}H-NMR (400 MHz, CD_2Cl_2)

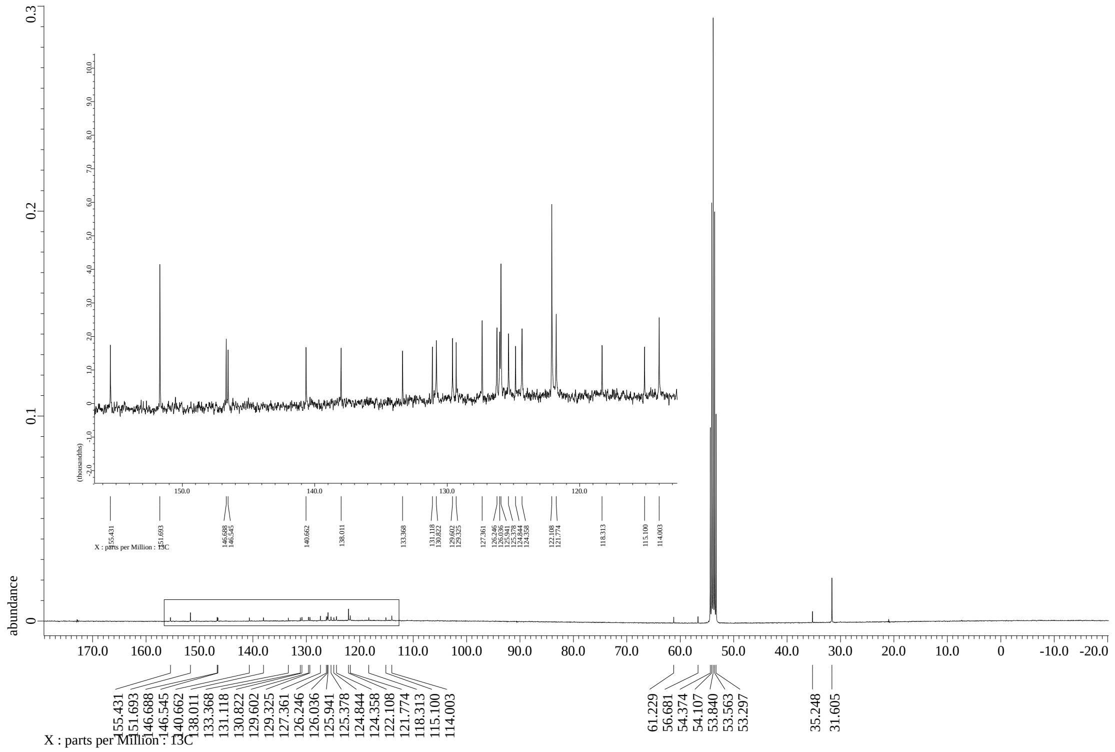

Figure A 18: ^{13}C-NMR (100.5 MHz, CD_2Cl_2)

4,10-Bis(6-(3,5-di-*tert*-butylphenyl)-2-methoxynaphthalen-1-yl)-5,11-dimethoxyxantheno[2,1,9,8-*klmna*]xanthene

(±) *Trans*-like diastereoisomer **19^H^**

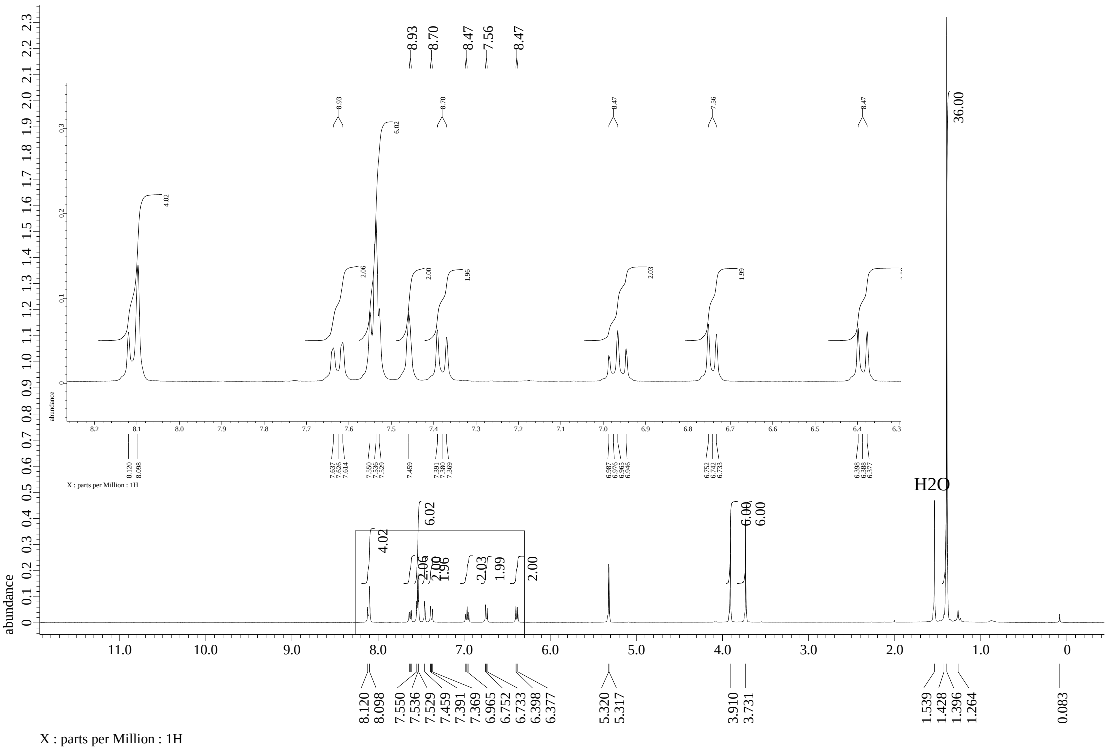

Figure A 19: ^1^H-NMR (400 MHz, CD_2Cl_2)

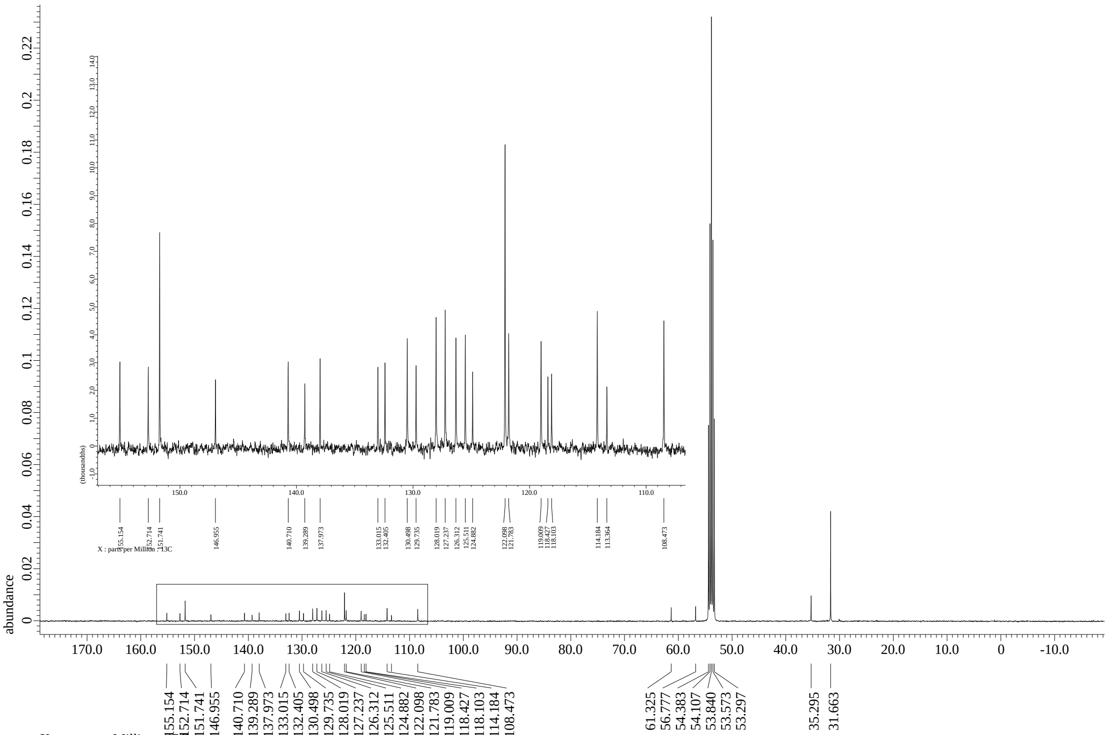

Figure A 20: ^13^C-NMR (100.5 MHz, CD_2Cl_2)

(±) *Cis*-like diastereoisomer **19^{H}**

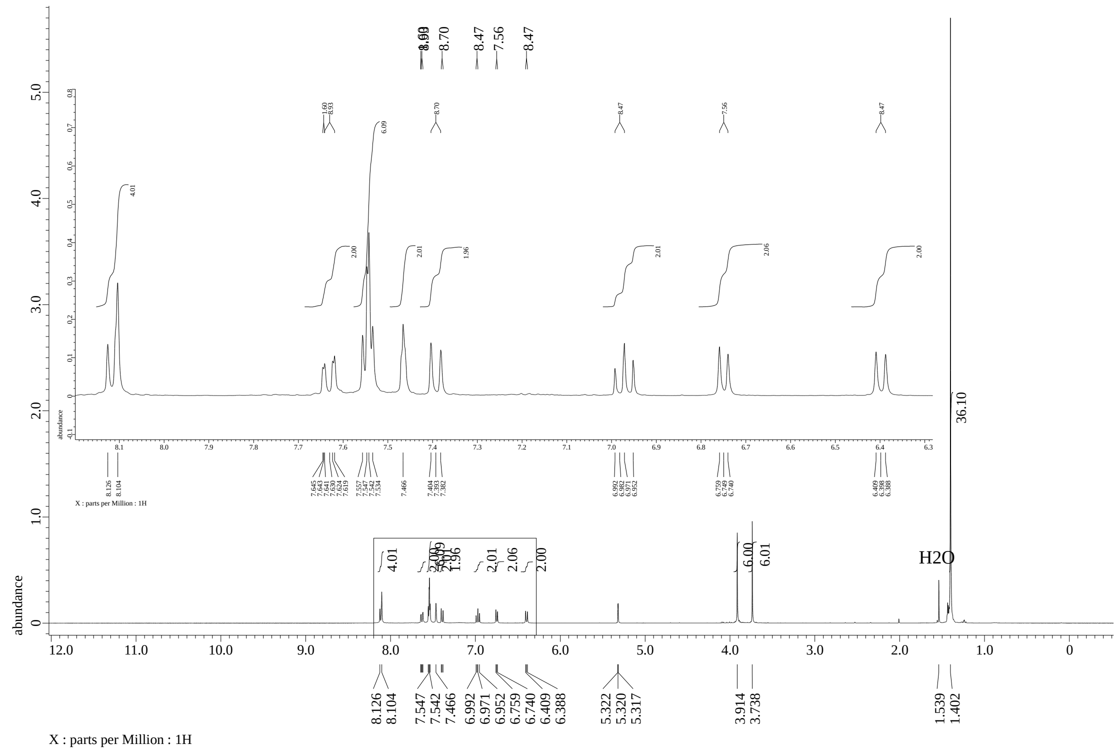

Figure A 21: ^{1}H-NMR (400 MHz, CD_2Cl_2)

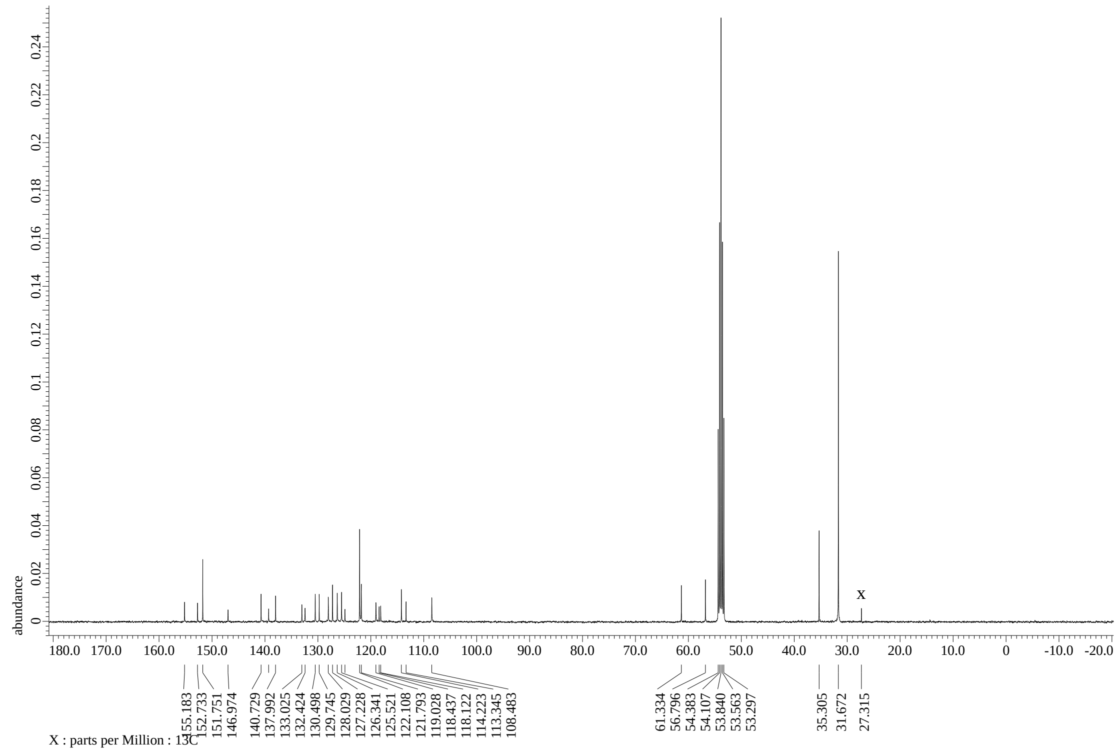

Figure A 22: ^{13}C-NMR (100.5 MHz, CD_2Cl_2)

4,10-Bis(6-(3,5-di-*tert*-butylphenyl)-2-hydroxynaphthalen-1-yl)xantheno[2,1,9,8-*klmna*]xanthene-5,11-diol **20^{H}**

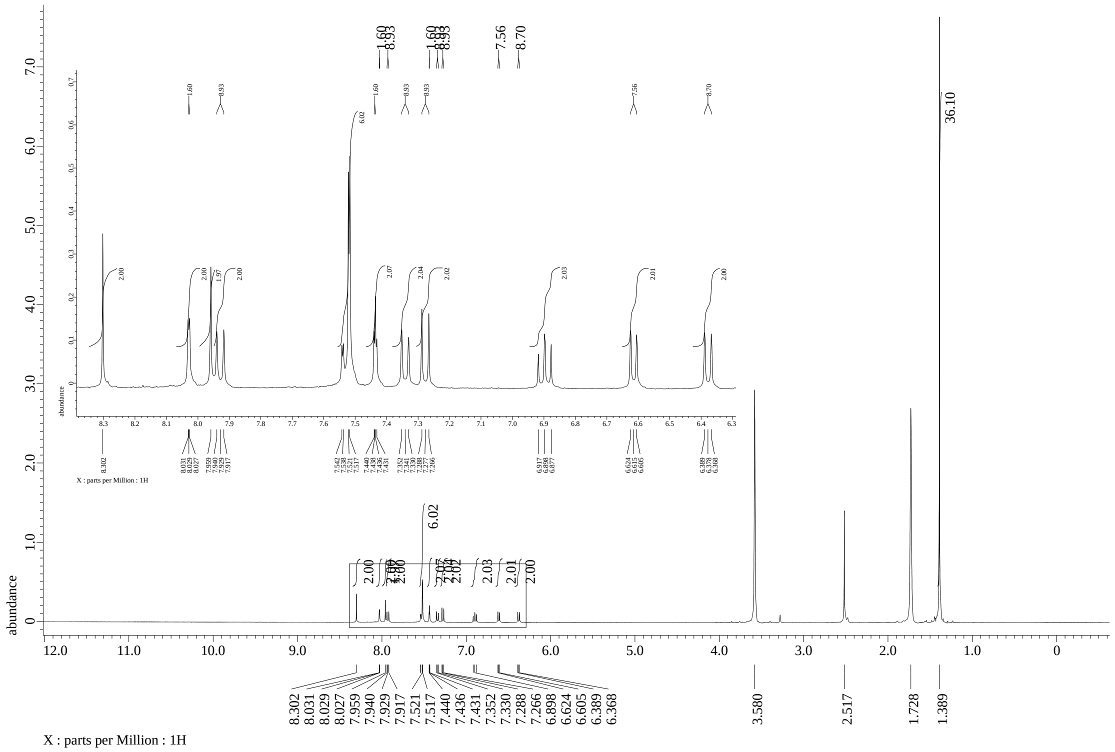

Figure A 23: ^{1}H-NMR (400 MHz, THF-d_6)

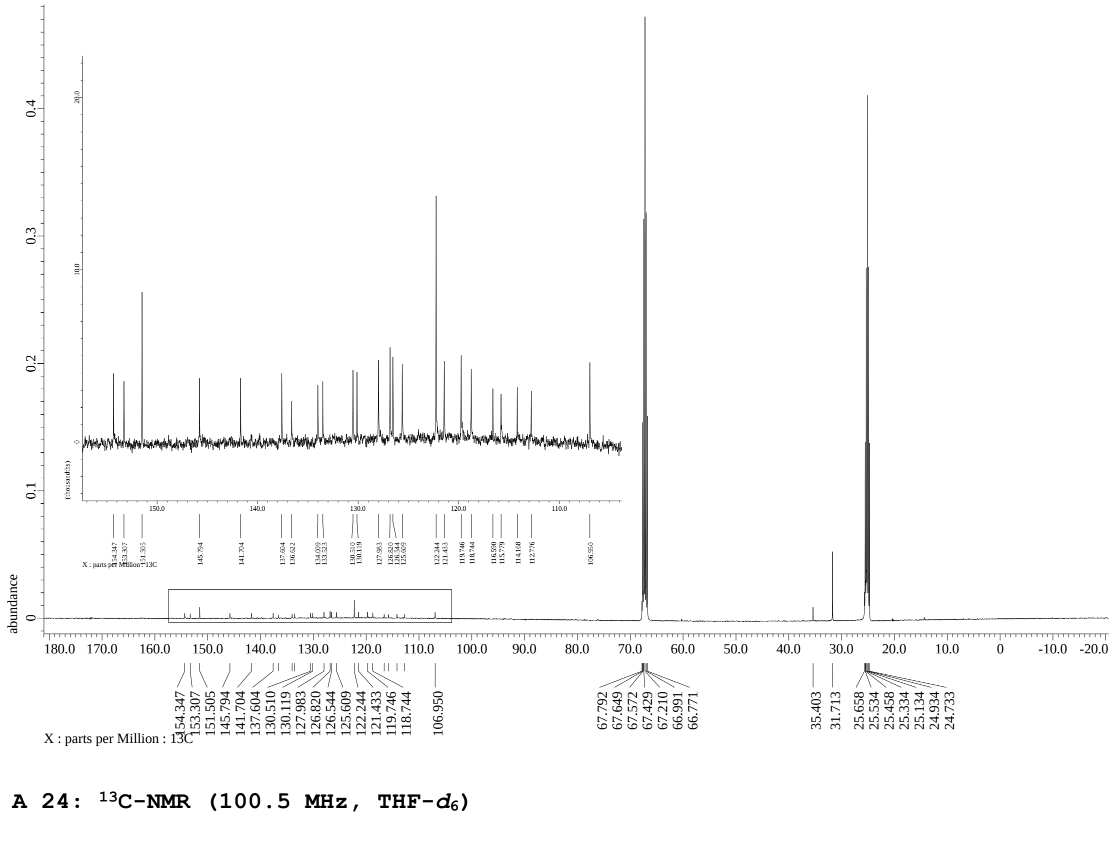

Figure A 24: ^{13}C-NMR (100.5 MHz, THF-d_6)

3-(Benzyloxy)-6,7-dibromonaphthalen-2-ol **2**

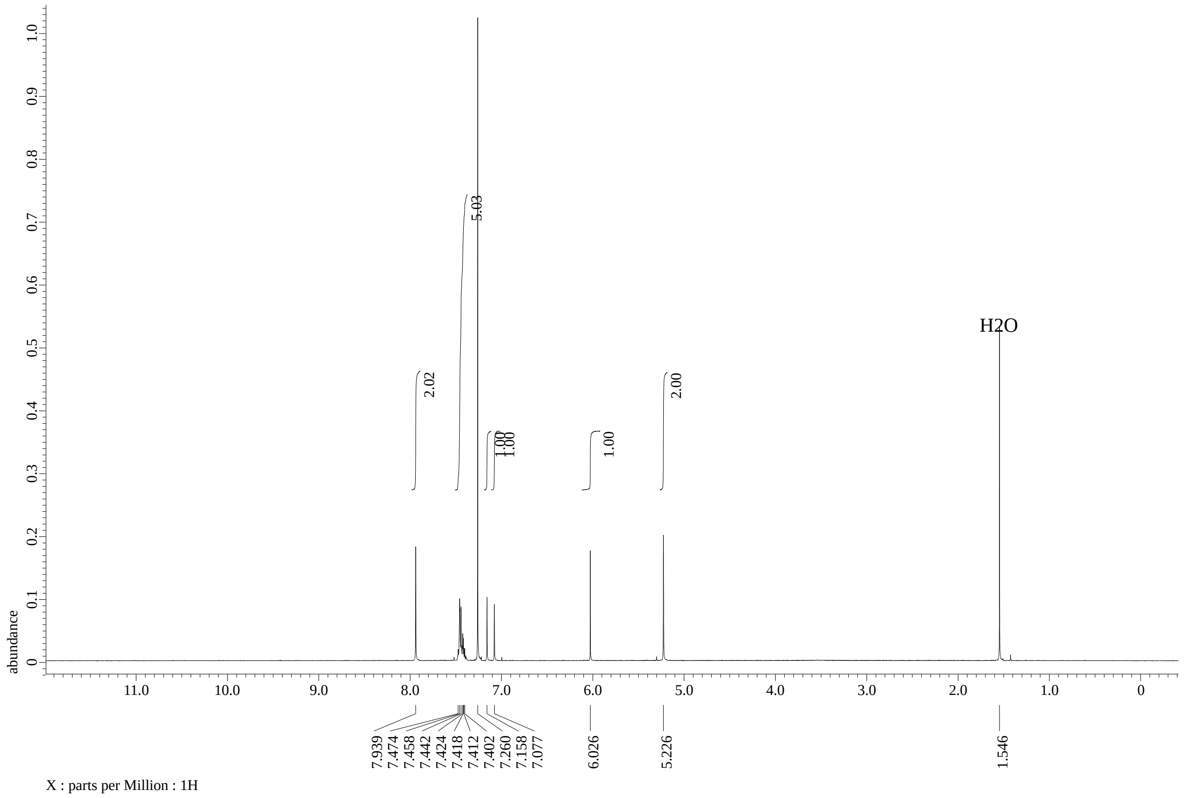

Figure A 25: ^{1}H-NMR (400 MHz, $CDCl_3$)

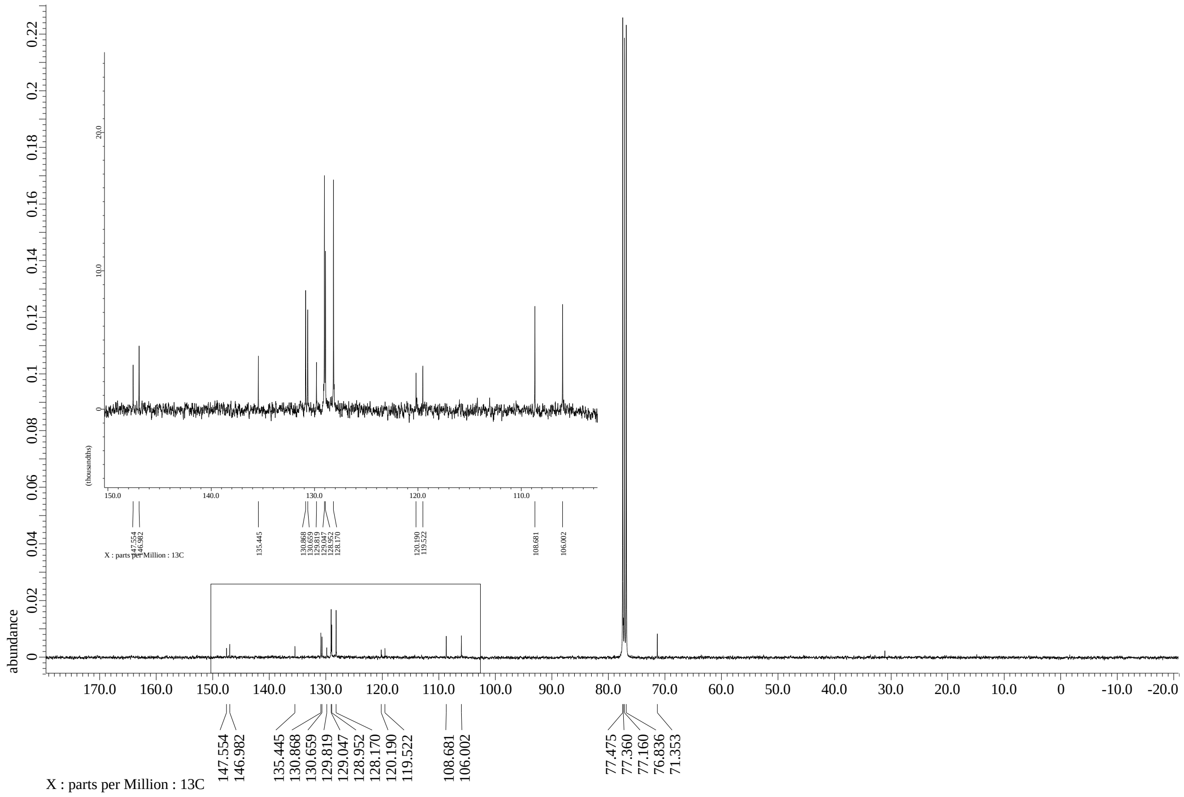

Figure A 26: ^{13}C-NMR (100.5 MHz, $CDCl_3$)

3-(Benzyloxy)-6,7-bis(4-(*tert*-butyl)phenyl)naphthalen-2-ol **4^tBuPh^**

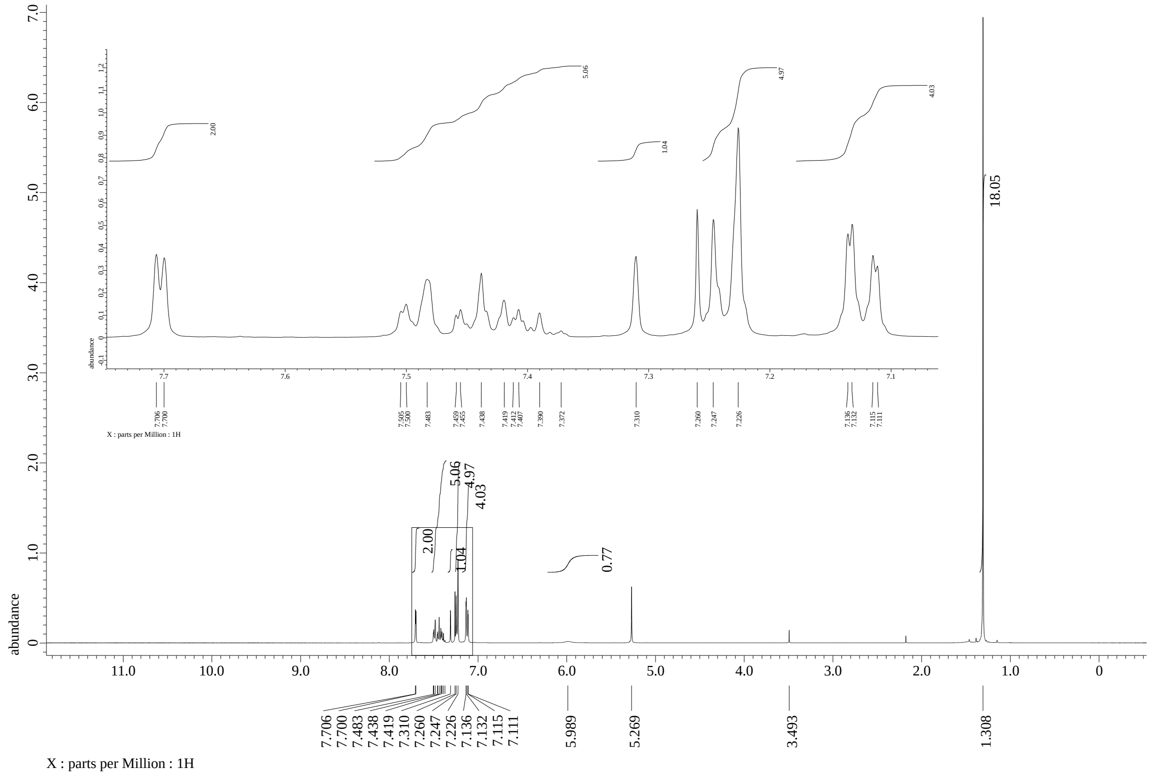

Figure A 27: ^{1}H-NMR (400 MHz, $CDCl_3$)

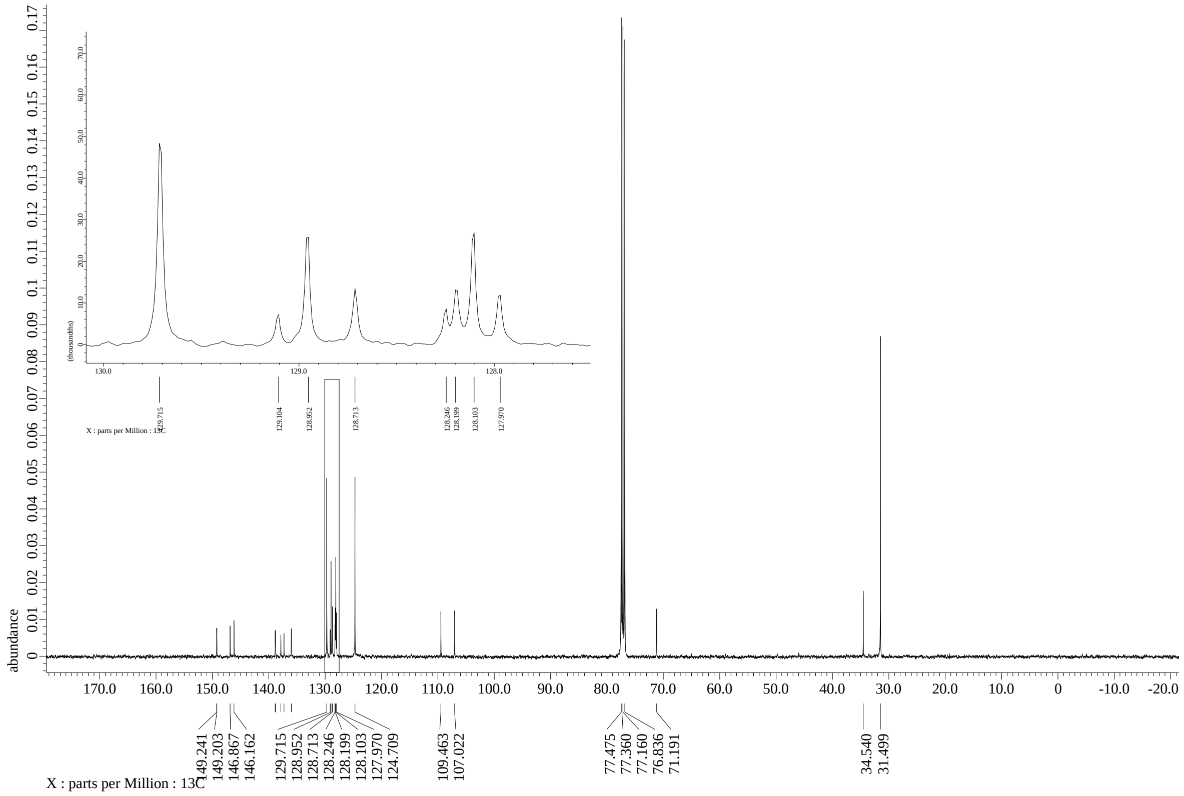

Figure A 28: ^{13}C-NMR (100.5 MHz, $CDCl_3$)

3-(Benzyloxy)-6,7-bis(4-(*tert*-butyl)phenyl)-6'-(3,5-di-*tert*-butylphenyl)-2,2'-dimethoxy-1,1'-binaphthalene **10^{tBuPh}**

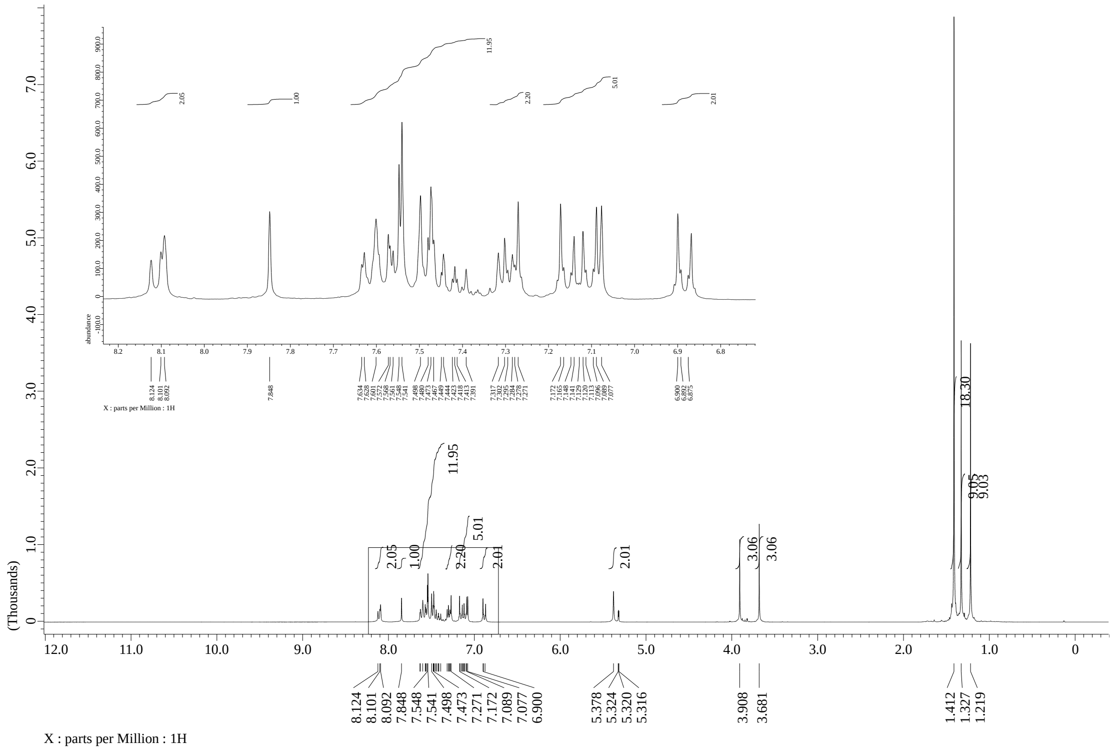

Figure A 29: ^{1}H-NMR (270 MHz, CD_2Cl_2)

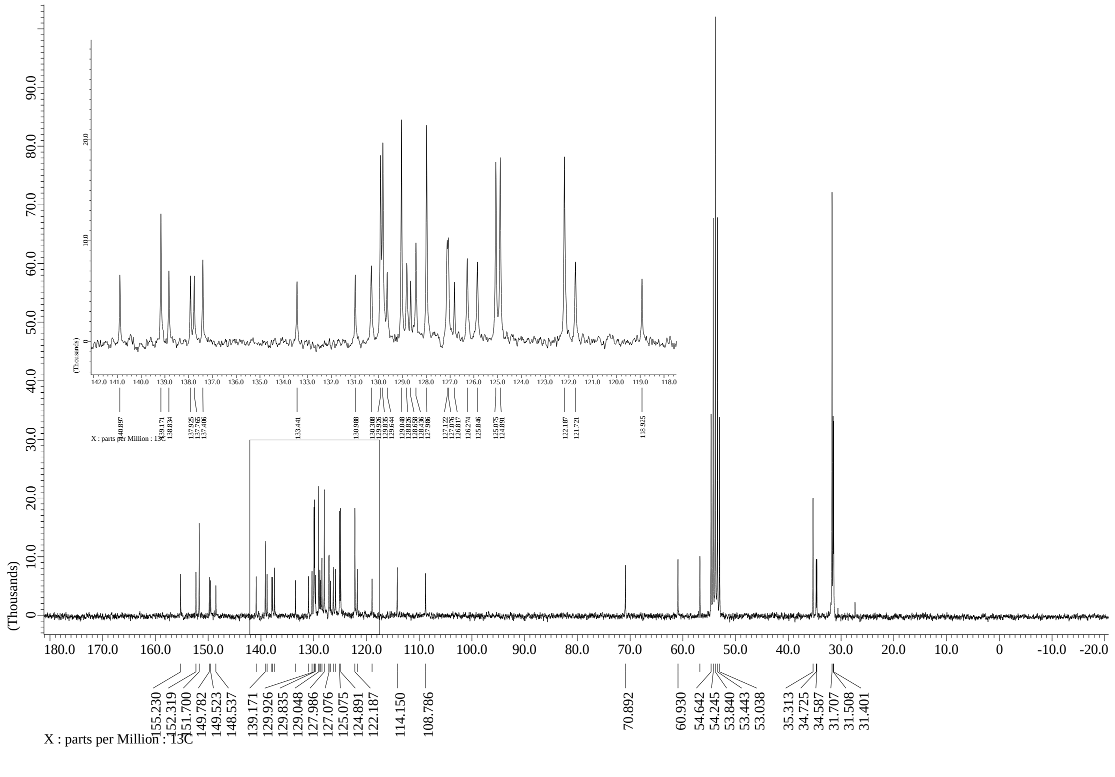

Figure A 30: ^{13}C-NMR (68 MHz, CD_2Cl_2)

6,7-Bis(4-(*tert*-butyl)phenyl)-6'-(3,5-di-*tert*-butylphenyl)-2,2'-dimethoxy-[1,1'-binaphthalen]-3-ol **11^{tBuPh}**

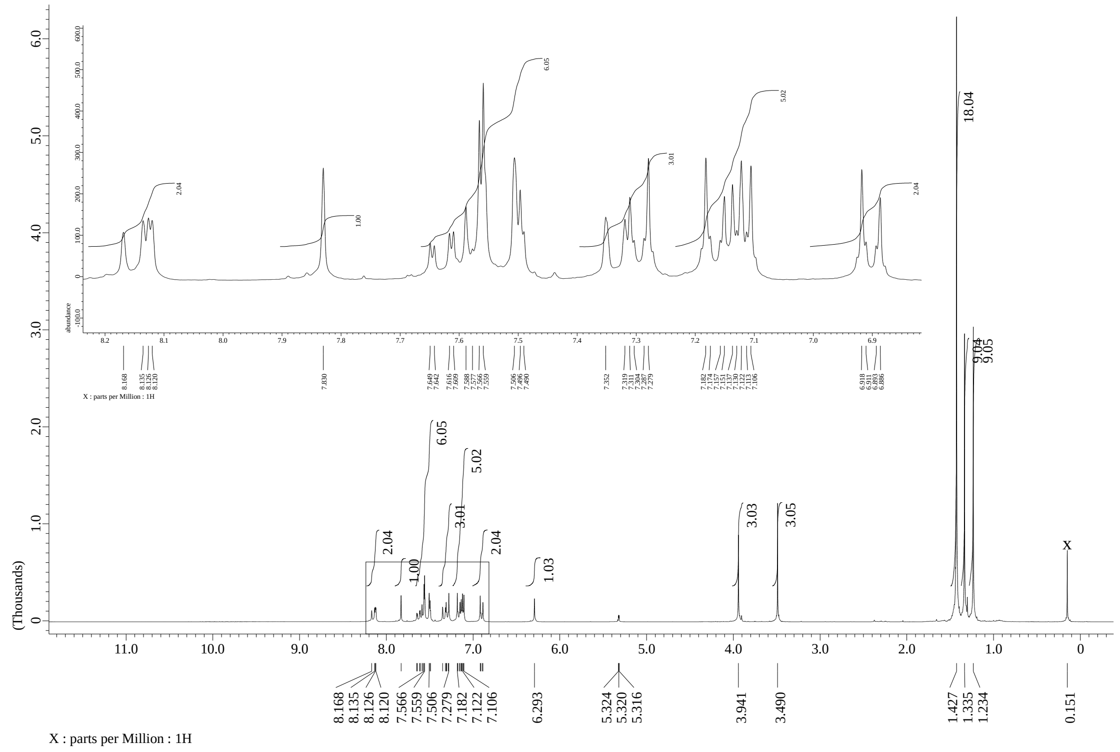

Figure A 31: ^{1}H-NMR (270 MHz, CD_2Cl_2)

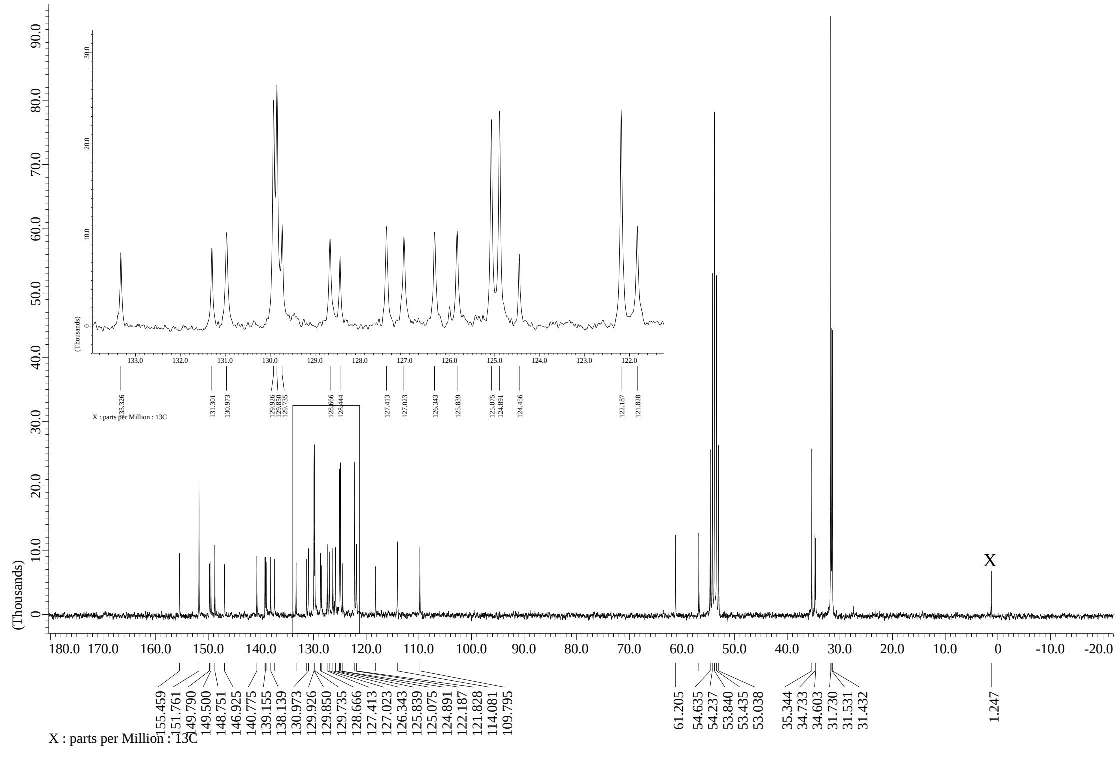

Figure A 32: ^{13}C-NMR (68 MHz, CD_2Cl_2)

1,2-Bis(4-(*tert*-butyl)phenyl)-8-(3,5-di-*tert*-butylphenyl)-4-(6-(3,5-di-*tert*-butylphenyl)-2-methoxynaphthalen-1-yl)-5-methoxyxantheno[2,1,9,8-*klmna*]xanthene **15**[tBuPh]

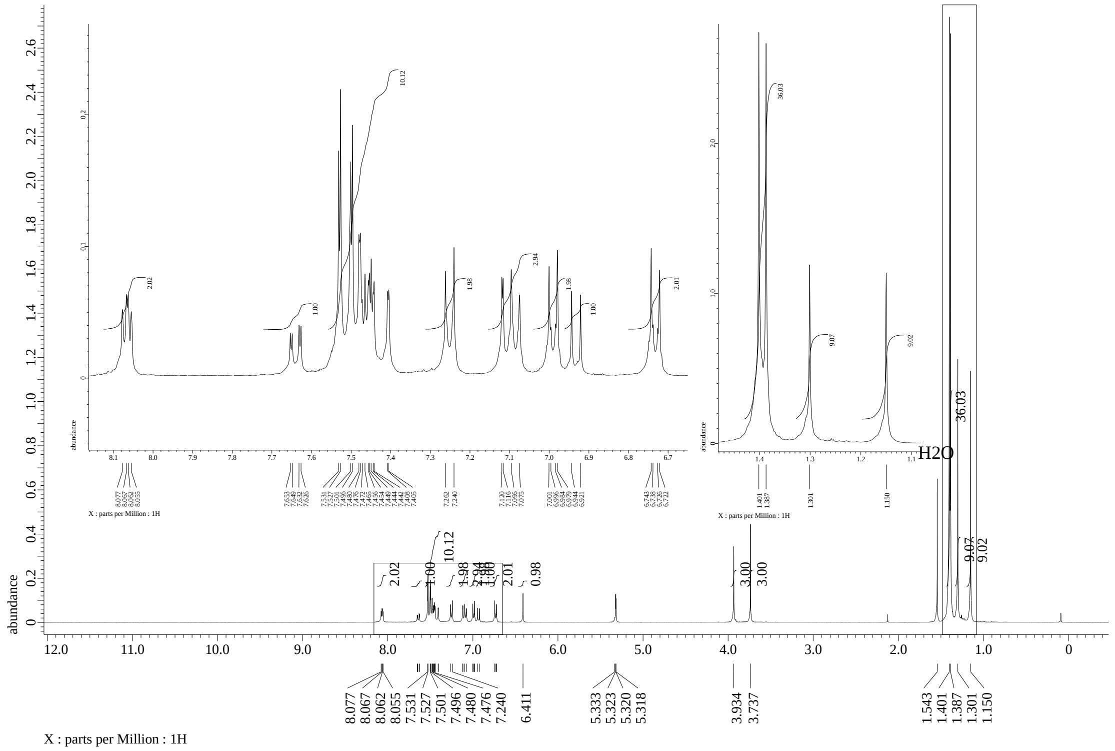

Figure A 33: ^{1}H-NMR (400 MHz, CD_2Cl_2)

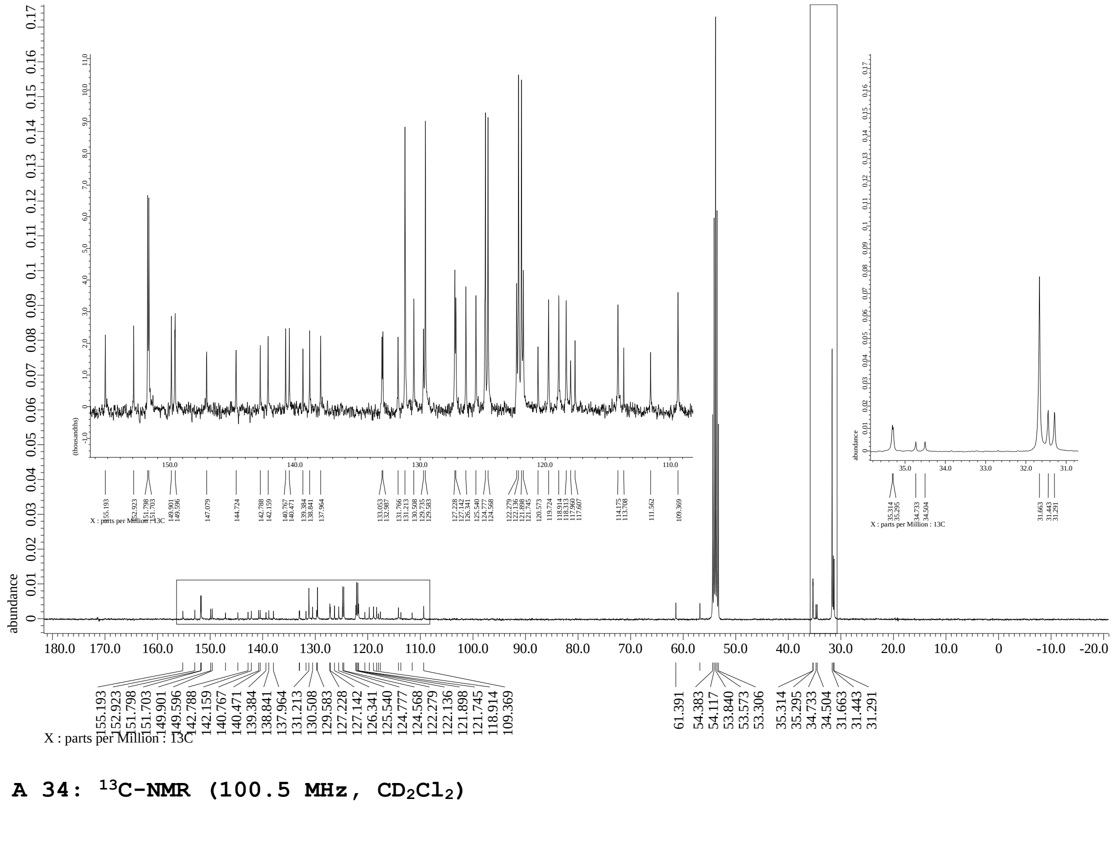

Figure A 34: ^{13}C-NMR (100.5 MHz, CD_2Cl_2)

1,2-Bis(4-(*tert*-butyl)phenyl)-8-(3,5-di-*tert*-butylphenyl)-4-(6-(3,5-di-*tert*-butylphenyl)-2-hydroxynaphthalen-1-yl)xantheno[2,1,9,8-klmna]xanthen-5-ol **16^{tBuPh}**

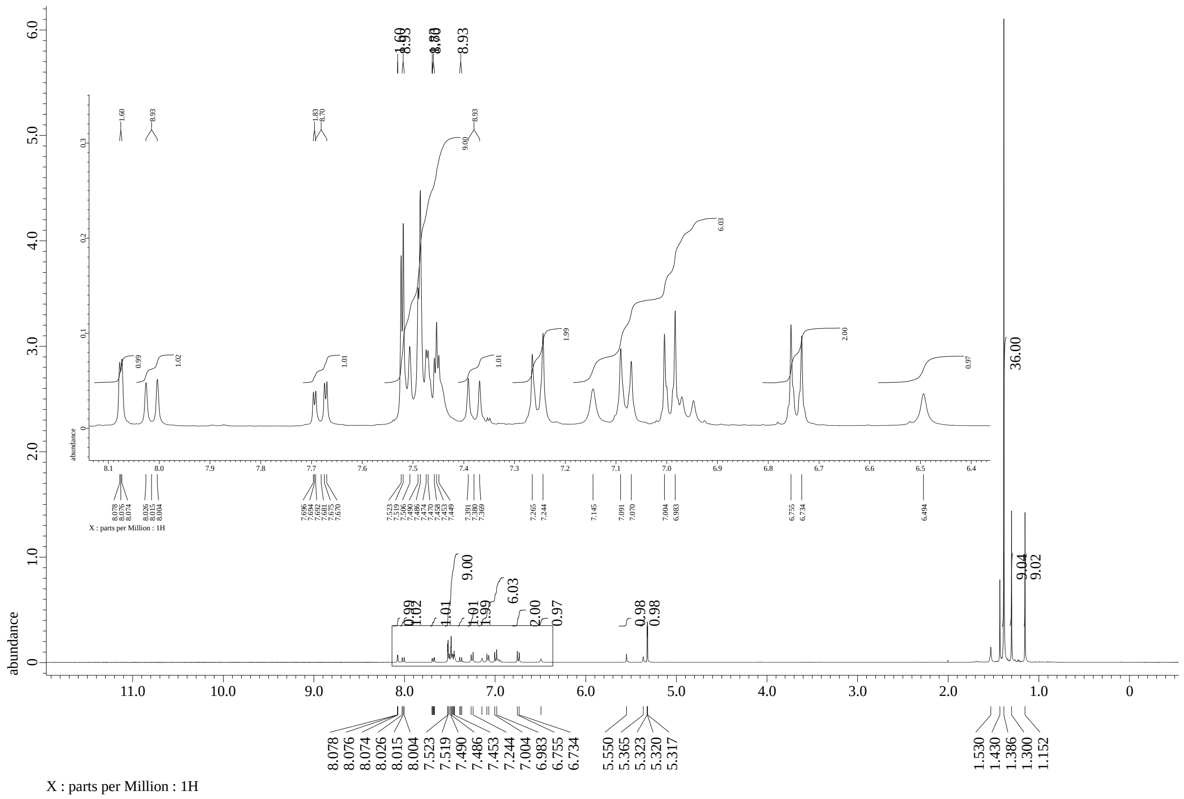

Figure A 35: ^{1}H-NMR (400 MHz, CD_2Cl_2)

6',6'',7',7''-Tetrakis(4-(*tert*-butyl)phenyl)-6,6'''-bis(3,5-di-*tert*-butylphenyl)-2,2',2''',3''-tetramethoxy-[1,1':4',1'':4'',1'''-quaternaphthalene]-2'',3'-diol **18^tBuPh^**

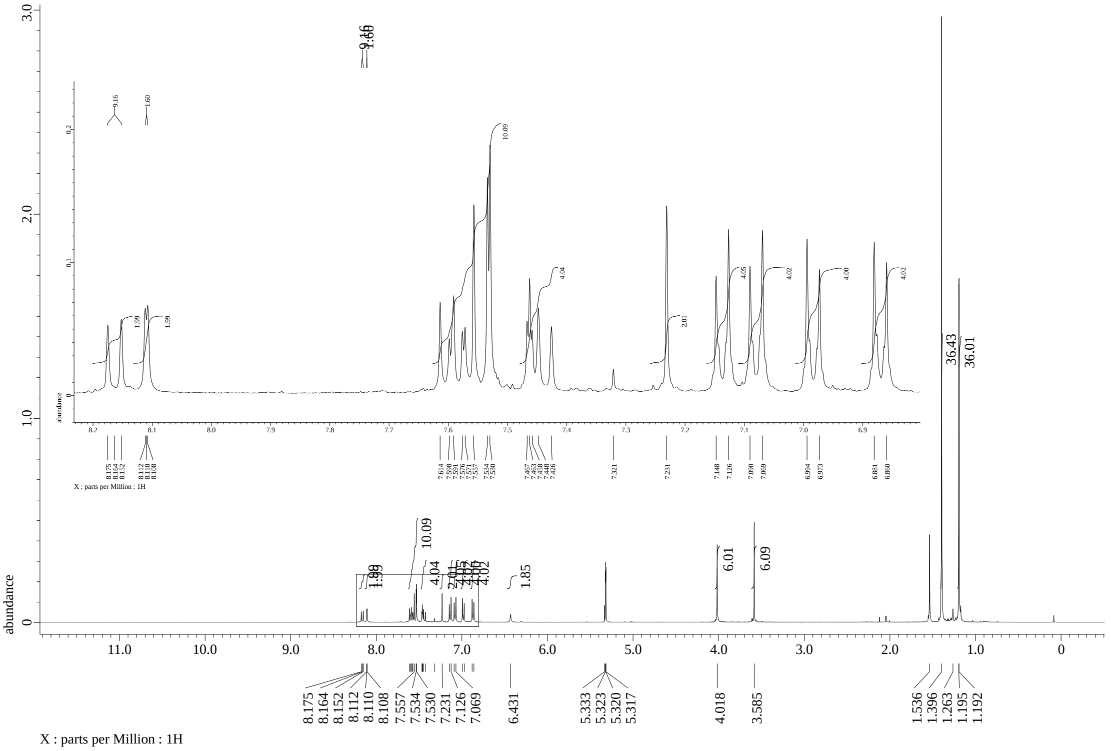

Figure A 36: ^{1}H-NMR (400 MHz, CD_2Cl_2)

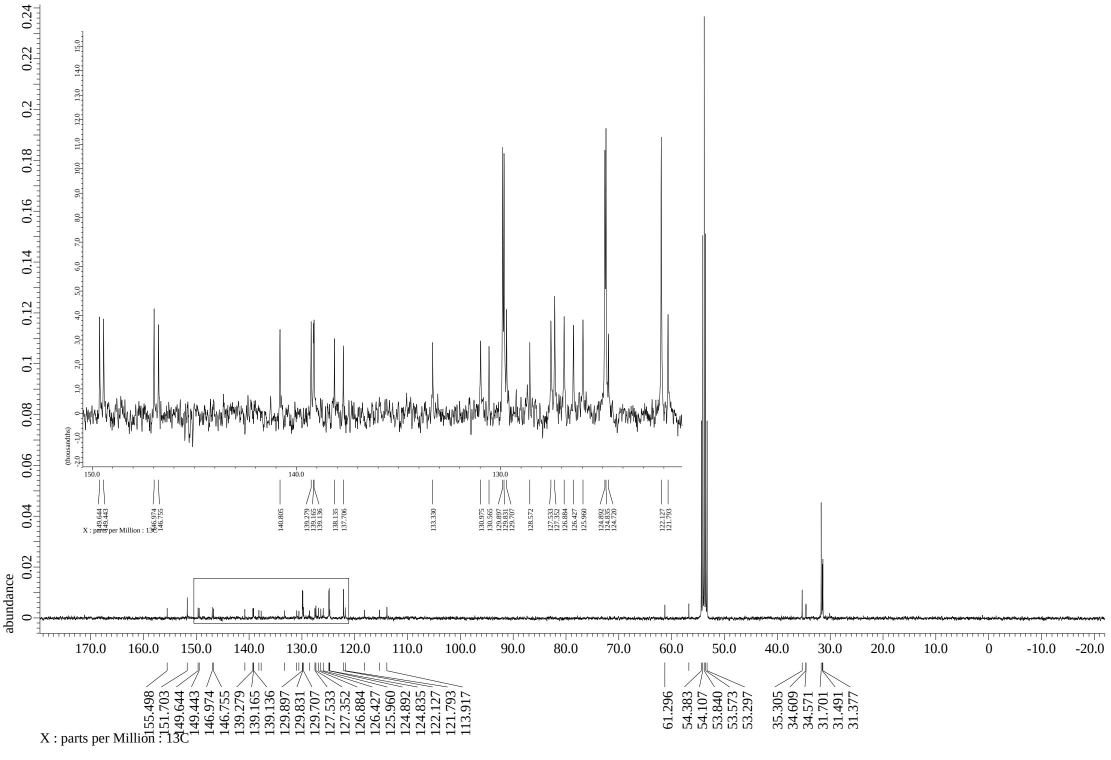

Figure A 37: ^{13}C-NMR (100.5 MHz, CD_2Cl_2)

1,2,7,8-Tetrakis(4-(*tert*-butyl)phenyl)-4,10-bis(6-(3,5-di-*tert*-butylphenyl)-2-methoxynaphthalen-1-yl)-5,11-dimethoxyxantheno[2,1,9,8-*klmna*]xanthene
(±) *trans*-like diastereoisomer **19^{tBuPh}**

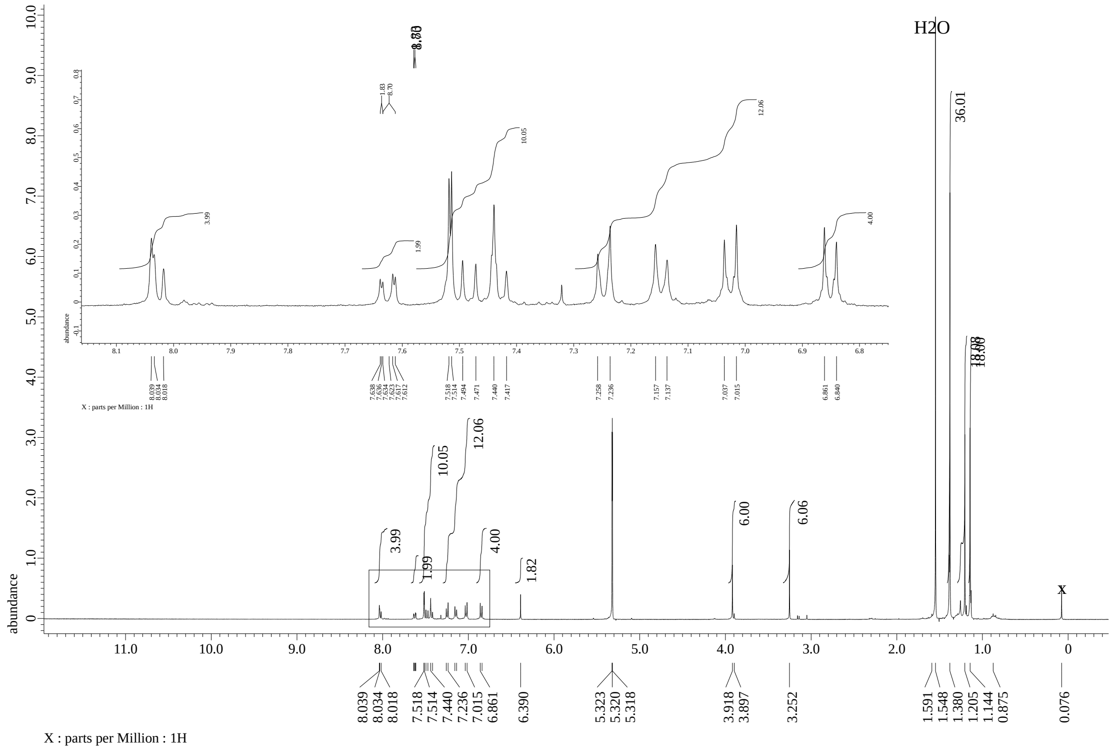

Figure A 38: ^1H-NMR (400 MHz, CD_2Cl_2)

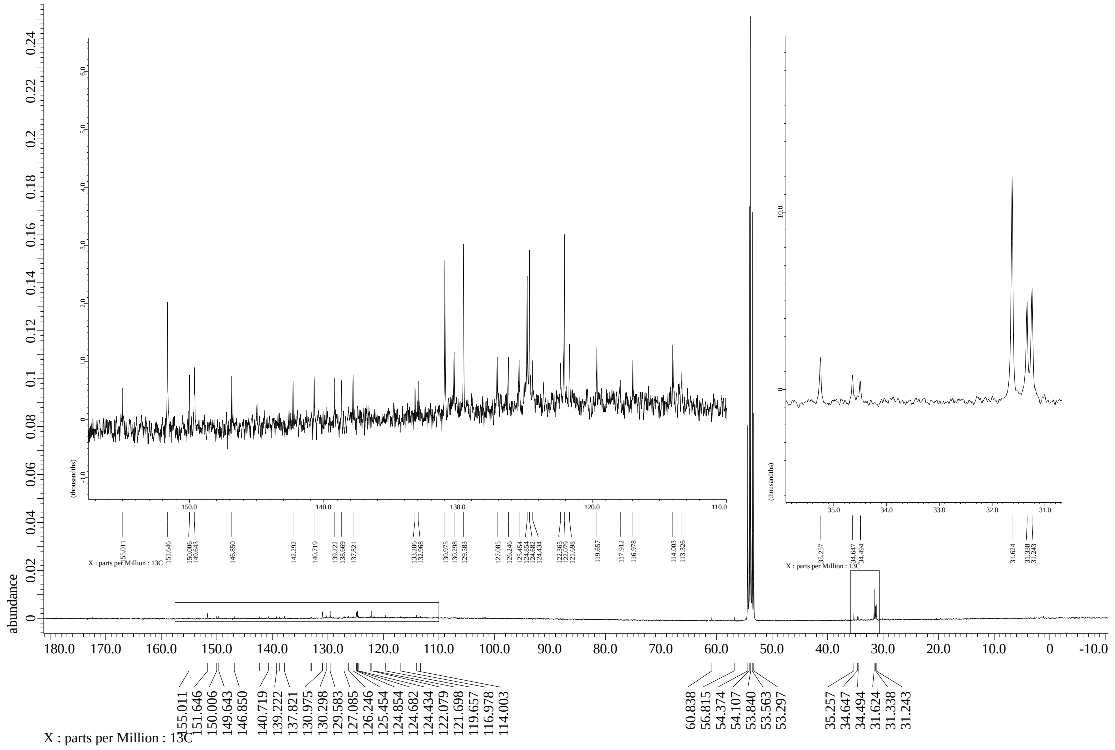

Figure A 39: ^{13}C-NMR (100.5 MHz, CD_2Cl_2)

(±) *Cis*-like diastereoisomer **19^{tBuPh}**

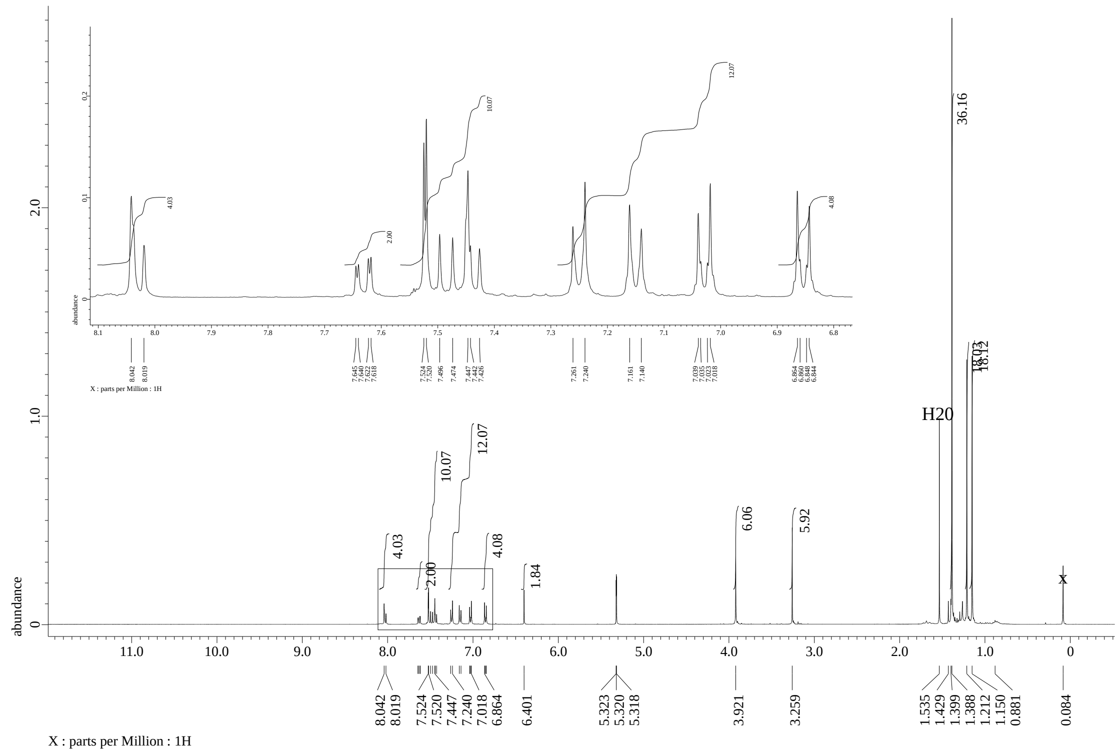

Figure A 40: ^{1}H-NMR (400 MHz, CD_2Cl_2)

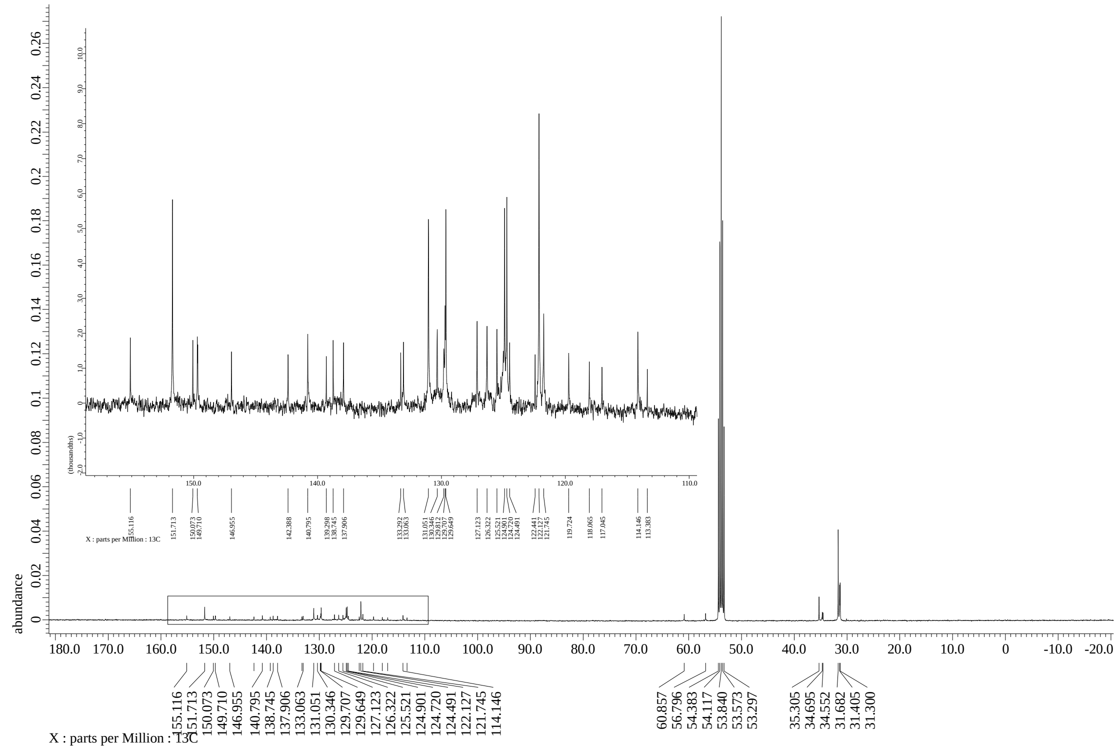

Figure A 41: ^{13}C-NMR (100.5 MHz, CD_2Cl_2)

1,2,7,8-Tetrakis(4-(*tert*-butyl)phenyl)-4,10-bis(6-(3,5-di-*tert*-butylphenyl)-2-hydroxynaphthalen-1-yl)xantheno[2,1,9,8-*klmna*]xanthene-5,11-diol **20^tBuPh^**

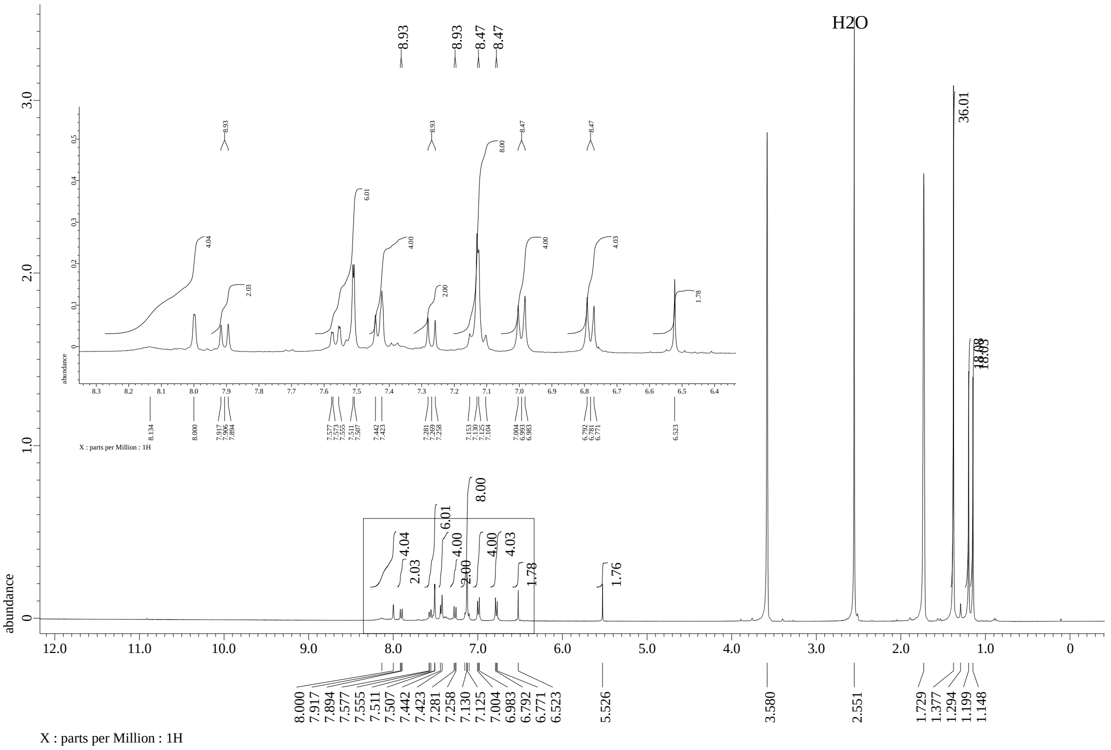

Figure A 42: ^{1}H-NMR (400 MHz, THF-d_6)

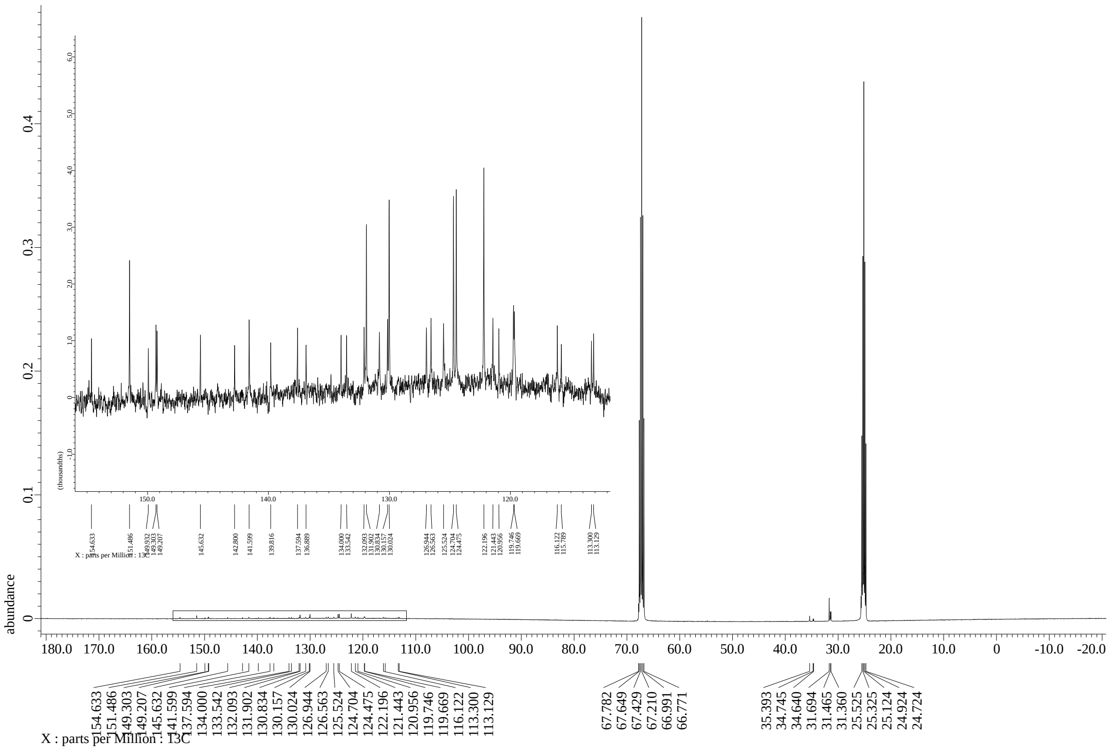

Figure A 43: ^{13}C-NMR (100.5 MHz, THF-d_6)

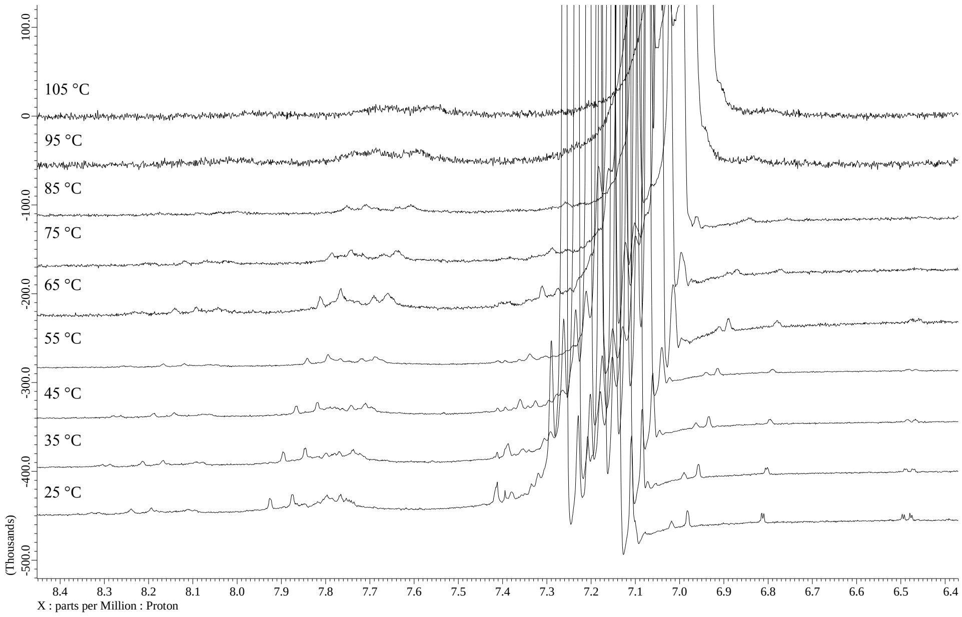

Fig. SI8. Variable temperature ^{1}H-NMR spectra (500 MHz, Toluene) of **21^{H}** (from r.t. to 105 °C).

References

[1S] P. T. Lynett, K. E. Maly, *Org. Lett.* **2009**, *11*, 3726–3729.

[2S] J. Ranta, T. Kumpulainen, H. Lemmetyinen, A. Efimov, *J. Org. Chem.* **2010**, *75*, 5178–5194.

[3S] W. Kabsch, *Acta Cryst. D* **2010**, *66*, 125–132.

[4S] M. C. Burla, R. Caliandro, B. Carrozzini, G. L. Cascarano, C. Cuocci, C. Giacovazzo, M. Mallamo, A. Mazzone, G. Polidori, *J. Appl. Cryst.* **2015**, *48*, 306-309.

[5S] G. M. Sheldrick, *Acta Cryst.* **2008**, *A64*, 112-122.

[6S] P. Emsley, K. Cowtan, *Acta Cryst. D* **2004**, *60*, 2126–2132.

[7S] A. L. Spek, *Acta Cryst. D* **2009**, *65*, 148–155.
